# Isolation, identification and bioactivities of abietane diterpenoids from *Premna szemaoensis*†

**DOI:** 10.1039/c7ra13309j

**Published:** 2018-02-09

**Authors:** De-Bing Pu, Ting Wang, Xing-Jie Zhang, Jun-Bo Gao, Rui-Han Zhang, Xiao-Nian Li, Yong-Mei Wang, Xiao-Li Li, He-Yao Wang, Wei-Lie Xiao

**Affiliations:** Key Laboratory of Medicinal Chemistry for Natural Resource of Ministry of Education, School of Chemical Science and Technology, State Key Laboratory for Conservation and Utilization of Bio-Resources in Yunnan, Yunnan University Kunming 650091 People's Republic of China lixiaoli@ynu.edu.cn xiaoweilie@ynu.edu.cn +86 871-665033214 +86 871-67357014 +86 871-665033214 +86 871-67357014; State Key Laboratory of Drug Research, Shanghai Institute of Materia Medica, Chinese Academy of Sciences Shanghai 201203 People's Republic of China hywang@simm.ac.cn +86 021-50807088 +86 021-50805785; State Key Laboratory of Phytochemistry and Plant Resources in West China, Kunming Institute of Botany, Chinese Academy of Sciences Kunming 650201 People's Republic of China; University of Chinese Academy of Sciences Beijing 100049 People's Republic of China

## Abstract

Investigation of the leaves and stems of *Premna szemaoensis* resulted in the isolation of twelve new abietane diterpenoids, szemaoenoids A–L (1–12), together with four known abietane diterpenoids (13–16). The structures involved two rearranged-abietane skeletons: 17(15 → 16)-*abeo*-abietane (7, 10–12, 14 and 15) and 17(15 → 16),18(4 → 3)-*diabeo*-abietane (1–6, 13 and 16). The structures of the new compounds were established mainly by analyzing NMR and HRESIMS data. The absolute configurations of 1, 3 and 10 were confirmed by single crystal X-ray diffraction analysis. In bioactivity assays, compounds 11, 12, 14 and 15 were active against two human colon cancer cell lines (HCT-116 and HT-29) with IC_50_ values ranging from 8.8 to 34.3 μM, and compounds 10, 13 and 14 exhibited effective free radical scavenging activity with IC_50_ values ranging from 35.6 to 41.5 μM by DPPH experiment.

## Abstract 2

Active abietane diterpenoids with two rearranged-abietane skeletons: 17(15 → 16)-abeo-abietane and 17(15 → 16),18(4 → 3)-diabeo-abietane were isolated from *Premna szemaoensis*.

## Graphical abstract

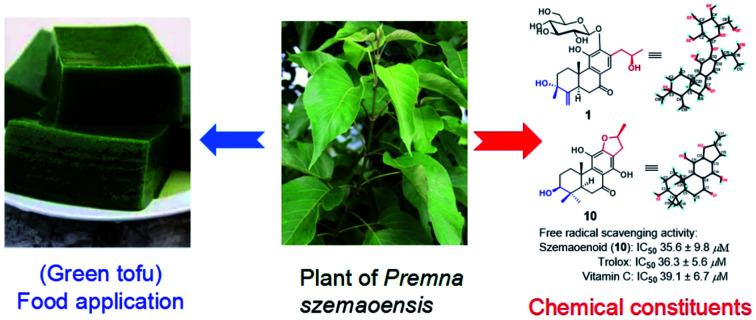

## Introduction

The genus *Premna* (family: Verbenaceae) comprises approximately 200 species, which are mainly distributed in the tropical zone of Asia and Africa.^1^ There are about 44 species and 5 varieties grown in the south of China, especially in Southwest China. The dried aerial parts of some *Premna* species have been used in traditional folk medicine for the treatment of pyogenic infections, trauma, fracture, dysentery, haemorrhoids, and rheumatic arthritis.^2^ Previous phytochemical investigations of *Premna* have indicated the presence of diterpenoids,^3^ flavonoids,^3–5^ iridoid glycosides,^4–7^ xanthones,^8^ phenylethanoid glycosides,^9^ triterpenoids,^10,11^ and lignins.^12^ Their pharmacological effects, including neuroprotective,^13^ analgesic,^14,15^ antioxidative, cytotoxic,^16,17^ anti-inflammatory,^18^ and α-glucosidase inhibition,^19^ have been reported for crude extracts and pure compounds from *Premna* plants.
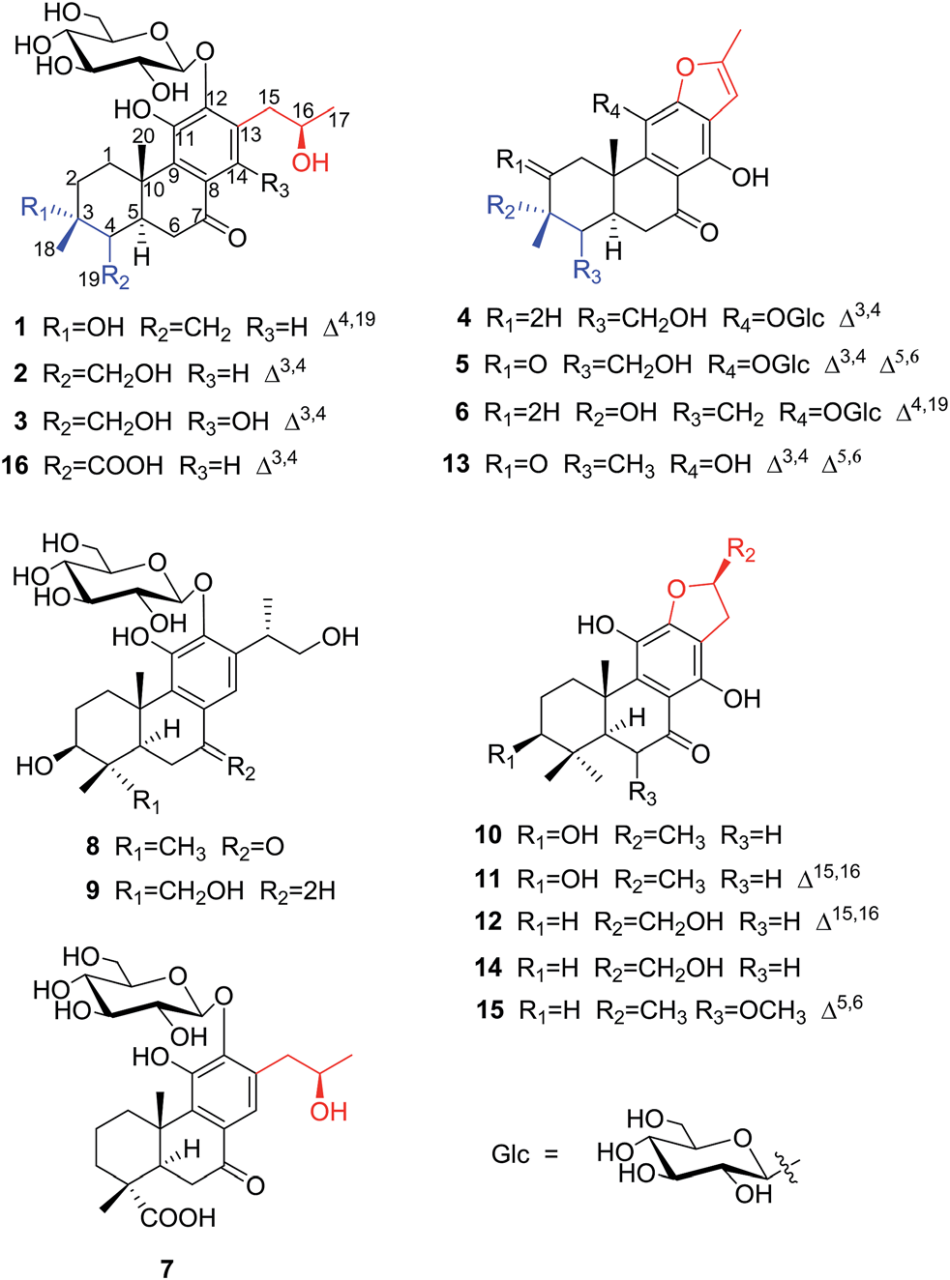


*Premna szemaoensis* Pei, locally called “simao dofu chai”, is mainly distributed in the south of Yunnan province of China.^20^ This plant has drawn the attention of local farmers and has been cultivated to be an important commercial crop due to its various applications. Its fresh leaves can be rubbed and squeezed in water to yield a mucilaginous juice, which was used to prepare a food named “green tofu” by local people through addition of materials containing Ca^2+^. In addition, the local villagers also used the leaves of this plant to cure injuries and fracture.^20^ However, the phytochemical investigation of this species was extremely rare, except a small number of flavonoids. In this investigation, we firstly afforded 12 new abietane diterpenoids (1–12) and four known abietane diterpenoids (13–16) from the aerial parts of *P. szemaoensis*. Most of the diterpenoids were with rearranged-abietane skeleton: 17(15 → 16)-*abeo*-abietane framework or 17(15 → 16),18(4 → 3)-*diabeo*-abietane framework, which were mainly isolated from the plants of genus *Clerodendron* and reported bioactivities including cytotoxic, angiotensin converting enzyme (ACE) inhibitory, antiviral actives.^21–24^ Herein, we describe the isolation and structural elucidation of these diterpenoids and the biological activities of selected compounds.

## Results and discussion

The aerial parts of *P. szemaoensis* were extracted three times with 70% acetone aqueous. After recycling acetone, the rest of portion was partitioned by liquid–liquid extraction between *n*-butanol and H_2_O. The *n*-butanol-soluble portion was repeatedly subjected to silica gel, Sephadex LH-20, and RP-C18 gel column chromatography (CC) and semi-preparative HPLC to afford 16 abietane diterpenoids, including 12 new compounds. The structures and stereochemistry of these isolates were elucidated mainly using spectroscopic analysis, X-ray diffraction analysis, and compared to data in the literature. Ultimately, the new compounds were named as szemaoenoids A–L (1–12), and known compounds were identified as teuvincenone F (13),^25^, (16*R*)-12,16-epoxy-11,14,17-trihydroxy-17(15 → 16)-*abeo*-8,11,13-abieta-triene-7-one (villosin B) (14),^26,27^ 12,16-epoxy-11,14,17-trihydroxy-6-methoxy-17(15 → 16)-*abeo*-5,8,11,13-abietatetraene-7-one (15),^28^ 11,16-dihydroxy-12-*O*-β-d-glucopyranosyl-17(15 → 16),18(4 → 3)-*diabeo*-4-carboxy-3,8,11,13-abietatetraene-7-one (16),^21^ respectively.

Compound 1 was isolated as an optically active, white monoclinic crystals (MeOH); [*α*]^25^_D_ +36.3 (*c* 0.10, MeOH). Its HRESIMS data showed a sodium adduct ion [M + Na]^+^ at *m*/*z* 531.2205 (calcd. for C_26_H_36_NaO_10_, 531.2206), which together with ^13^C NMR (Table 2) and DEPT data were consistent with a molecular formula of C_26_H_36_O_10_, representing nine indices of hydrogen deficiency. The ^1^H NMR spectrum of 1 (Table 1) showed one doublet and two singlets for methyl groups at *δ*_H_ 1.12 (d, *J* = 6.2), 1.41 (s) and 1.20 (s), respectively; three olefinic proton signals at *δ*_H_ 7.46 (s), 5.20 (s), and 4.74 (s), and a double peak at *δ*_H_ 4.57 (d, *J* = 7.9). The ^13^C NMR and DEPT spectra of 1 (Table 2) showed signals of 20 carbons of an aglycon, attributable to a ketone group at *δ*_C_ 200.7, three methyls (two tertiary), five methylenes (one olefinic), three methines (one olefinic, one oxygenated), and eight quaternary carbons (six olefinic, one oxygenated), along with signals for a hexose unit. These data suggested that 1 is a diterpene glycoside and in accordance with the characteristics of a 17(15 → 16),18(4 → 3)-*diabeo*-8,11,13-abietatriene.

**Table 1:** ^1^H NMR data of compounds 1–12 measured at 600 MHz (*δ* in ppm, *J* in Hz)

Position	1^a^	2^a^	3^a^	4^a^	5^a^	6^a^	7^a^	8^a^	9^a^	10^b^	11^b^	12^b^
1α	1.77 (overlap)	1.51 (td, 12.7, 6.5)	1.53 (td, 12.6, 6.0)	1.68 (td, 12.6, 6.1)	2.54 (d, 16.9)	1.90 (td, 13.7, 4.1)	1.18 (overlap)	1.43 (overlap)	1.75 (m)	1.47 (br t, 14.2)	1.55 (br t, 14.0)	1.43 (overlap)
1β	3.16 (br d, 13.1)	3.52 (overlap)	3.52 (overlap)	3.56 (overlap)	4.29 (d, 16.9)	3.22 (overlap)	3.38 (d, 12.9)	3.45 (overlap)	1.87 (overlap)	3.46 (br d, 13.7)	3.55 (br d, 13.6)	2.57 (br d, 17.0)
2α	1.74 (overlap)	2.10 (dd, 18.5, 6.1)	2.09 (dd, 18.5, 6.0)	2.08 (dd, 18.2, 5.5)		1.74 (overlap)	1.46 (overlap)	1.75 (overlap)	1.55 (qd, 12.4, 5.1)	1.74 (overlap)	1.73 (m)	1.55 (m)
2β	1.74 (overlap)	2.27 (m)	2.28 (m)	2.32 (m)		1.74 (overlap)	2.12 (m)	1.81 (dd, 26.0, 12.8)	1.90 (overlap)	1.74 (overlap)	1.79 (overlap)	1.79 (overlap)
3α							1.00 (td, 13.1, 3.4)	3.28 (overlap)	3.37 (overlap)	3.27 (overlap)	3.30 (dd, 11.5, 5.3)	1.33 (td, 13.5, 3.6)
3β							2.25 (br d, 13.1)					1.50 (overap)
5	3.32 (overlap)	2.94 (br d, 15.4)	2.90 (overlap)	2.88 (br d, 14.5)		3.28 (overlap)	1.81 (br d, 14.2)	1.73 (overlap)	1.29 (overlap)	1.71 (overlap)	1.80 (dd, 14.5, 1.7)	1.84 (dd, 14.7, 2.1)
6α	2.37 (dd, 16.0, 2.4)	2.99 (dd, 16.8, 2.9)	2.69 (br t, 12.0)	3.04 (dd, 17.3, 3.2)	6.80 (s)	2.41 (dd, 16.6, 2.4)	2.89 (br d, 16.9)	2.66 (br t, 15.5)	1.23 (overlap)	2.49 (d, 17.0)	2.57 (br d, 17.0)	2.57 (d, 17.0)
6β	2.69 (overlap)	2.57 (t, 15.9)	3.01 (dd, 17.0, 2.7)	2.70 (dd, 17.3, 15.2)		2.85 (t, 15.7)	3.27 (overlap)	2.58 (d, 16.7)	3.40 (overlap)	2.70 (overlap)	2.78 (overlap)	2.76 (t, 15.9)
7α									2.71 (overlap)			
7β									2.76 (overlap)			
14	7.46 (s)	7.46 (s)					7.39 (s)	7.44 (s)	6.35 (s)			
15α	2.71 (overlap)	2.70 (dd, 13.3, 6.6)	2.87 (overlap)	6.55 (s)	6.62 (d, 0.9)	6.55 (s)	2.66 (dd, 13.3, 6.9)	3.77 (m)	3.68 (overlap)	2.73 (overlap)	6.55 (s)	6.77 (s)
15β	3.20 (dd, 13.4, 6.6)	3.18 (dd, 13.3, 6.6)	3.17 (dd, 13.0, 6.8)				3.20 (m)			3.26 (overlap)		
16α	4.10 (ddd, 6.2, 6.4, 6.6)	4.11 (ddd, 6.2, 6.6, 6.7)	4.15 (dd, 12.8, 6.4)				4.11 (ddd, 6.6, 6.4, 6.2)	3.50 (overlap)	3.42 (overlap)	5.09 (m)		
16β								3.61 (dd, 10.5, 6.6)	3.58 (dd, 10.5, 6.3)			
17	1.12 (d, 6.2)	1.12 (d, 6.2)	1.12 (d, 6.3)	2.46 (s)	2.49 (s)	2.46 (s)	1.11 (d, 6.2)	1.15 (d, 6.9)	1.11 (d, 6.9)	1.43 (d, 6.2)	2.40 (s)	4.64 (d, 5.9)
18α	1.41 (s)	1.26 (s)	1.24 (s)	1.68 (s)	2.06 (s)	1.40 (s)		1.03 (s)	3.45 (overlap)	1.05 (s)	1.07 (s)	0.99 (s)
18β									4.18 (d, 11.2)			
19α	4.74 (s)	4.07 (d, 11.7)	4.07 (d, 11.9)	4.09 (d, 12.0)	4.61 (overlap)	4.76 (s)	1.18 (s)	0.93 (s)	1.26 (s)	0.92 (s)	0.94 (s)	1.01 (s)
19β	5.20 (s)	4.27 (d, 11.7)	4.27 (d, 11.9)	4.26 (d, 12.0)	4.71 (overlap)	5.20 (s)						
20	1.20 (s)	1.78 (s)	1.77 (s)	1.36 (s)	1.73 (s)	1.32 (s)	1.43 (s)	1.40 (s)	1.28 (s)	1.39 (s)	1.44 (s)	1.47 (s)
1′	4.57 (d, 7.9)	4.57 (d, 7.9)	4.65 (d, 8.0)	5.41 (d, 7.2)	5.90 (d, 7.3)	5.59 (d, 7.4)	4.55 (d, 7.9)	4.46 (d, 7.9)	4.37 (d, 7.8)			
2′	3.51 (t, 8.5)	3.51 (t, 8.5)	3.52 (m)	3.49 (overlap)	3.53 (overlap)	3.47 (overlap)	3.50 (t, 8.4)	3.50 (overlap)	3.45 (overlap)			
3′	3.29 (overlap)	3.29 (overlap)	3.27 (overlap)	3.20 (m)	3.34 (overlap)	3.22 (m)	3.29 (overlap)	3.28 (overlap)	3.25 (overlap)			
4′	3.44 (overlap)	3.45 (overlap)	3.44 (t, 9.1)	3.33 (overlap)	3.34 (overlap)	3.29 (overlap)	3.44 (overlap)	3.27 (overlap)	3.27 (overlap)			
5′	3.43 (overlap)	3.42(overlap)	3.42 (t, 9.1)	3.49 (overlap)	3.53 (overlap)	3.47 (overlap)	3.43 (overlap)	3.41 (m)	3.39 (overlap)			
6′α												
6′β	3.75 (dd, 12.0, 4.8)	3.75 (dd, 12.0, 4.7)	3.75 (dd, 12.0, 4.7)	3.57 (overlap)	3.70 (br, 11.9)	3.54 (dd, 12.0, 5.7)	3.75 (dd, 10.3, 4.7)	3.66 (br d, 11.7)	3.65 (overlap)			
	3.84 (dd, 12.0, 1.8)	3.85 (dd, 12.0, 1.9)	3.84 (dd, 12.0, 1.6)	3.73 (dd, 11.8, 2.0)	3.54 (overlap)	3.70 (dd, 12.0, 2.0)	3.83 (br d, 10.3)	3.89 (br d, 11.7)	3.89 (d, 12.0)			
OH-3										3.60 (br s)	3.63 (s)	
OH-11										7.27 (s)	8.16 (s)	8.28 (s)
OH-14										13.39 (s)	13.8 (s)	13.8 (s)
OH-17												4.50 (s)

a In CD_3_OD solution.

b In acetone-*d*_6_ solution.

**Table 2:** ^13^C NMR data of compounds 1–12 measured at 150 MHz (*δ* in ppm)

Position	1^a^	2^a^	3^a^	4^a^	5^a^	6^a^	7^a^	8^a^	9^a^	10^b^	11^b^	12^b^
1	31.9, CH_2_	32.6, CH_2_	32.7, CH_2_	33.7, CH_2_	47.9, CH_2_	33.4, CH_2_	37.4, CH_2_	35.8, CH_2_	29.2, CH_2_	35.6, CH_2_	36.1, CH_2_	37.6, CH_2_
2	37.8, CH_2_	31.1, CH_2_	31.2, CH_2_	31.2, CH_2_	201.1, C	38.1, CH_2_	21.2, CH_2_	28.4, CH_2_	20.5, CH_2_	28.7, CH_2_	18.8, CH_2_	19.7, CH_2_
3	71.5, C	133.8, C	134.0, C	133.9, C	138.4, C	71.3, C	39.9, CH_2_	78.7, CH	80.9, CH	77.5, CH	77.5, CH	41.8, CH_2_
4	153.7, C	129.6, C	129.3, C	129.4, C	149.6, C	153.6, C	45.7, C	40.2, C	44.3, C	39.8, C	39.9, C	34.0, C
5	44.0, CH	44.5, CH	44.0, CH	43.9, CH	161.9, C	43.7, CH	53.6, CH	51.4, CH	55.0, CH	50.4, CH	50.4, CH	50.9, CH
6	38.6, CH_2_	38.0, CH_2_	38.3, CH_2_	38.5, CH_2_	124.9, CH	38.7, CH_2_	39.2, CH_2_	36.2, CH_2_	34.4, CH_2_	35.6, CH_2_	36.0, CH_2_	36.2, CH_2_
7	200.7, C	201.1, C	207.1, C	207.5, C	191.8, C	207.0, C	203.2, C	201.3, C	35.6, CH_2_	205.2, C	207.0, C	207.0, C
8	130.0, C	130.2, C	114.2, C	112.1, C	110.1, C	111.9, C	130.1, C	130.2, C	135.6, C	110.7, C	111.2, C	111.5, C
9	139.5, C	139.7, C	137.3, C	139.9, C	133.7, C	139.1, C	141.1, C	140.3, C	134.1, C	140.6, C	135.1, C	135.6, C
10	42.0, C	39.1, C	39.2, C	39.9, C	44.2, C	42.7, C	42.3, C	41.4, C	40.1, C	41.2, C	41.2, C	41.6, C
11	149.9, C	149.7, C	141.7, C	133.7, C	133.1, C	134.0, C	150.4, C	149.3, C	149.1, C	132.7, C	133.5, C	133.4, C
12	150.5, C	150.5, C	153.3, C	153.4, C	151.3, C	153.0, C	149.7, C	150.0, C	143.2, C	156.3, C	148.3, C	152.1, C
13	132.8, C	132.7, C	121.0, C	119.8, C	120.6, C	119.9, C	132.2, C	138.2, C	136.6, C	111.7, C	117.9, C	117.3, C
14	121.7, CH	122.1, CH	157.4, C	156.8, C	154.1, C	156.7, C	121.3, CH	117.2, CH	118.2, CH	155.9, C	153.6, C	154.1, C
15	40.9, CH_2_	40.9, CH_2_	33.6, CH_2_	101.5, CH	101.2, CH	101.4, CH	41.0, CH_2_	35.1, CH	34.9, CH	34.5, CH_2_	101.6, CH	102.5, CH
16	68.3, CH	68.3, CH	68.3, CH	156.5, C	157.4, C	155.7, C	68.3, CH	68.8, CH_2_	69.1, CH_2_	83.3, CH	155.9, C	158.8, C
17	22.8, CH_3_	22.9, CH_3_	22.9, CH_3_	13.7, CH_3_	13.8, CH_3_	13.7, CH_3_	22.8, CH_3_	18.2, CH_3_	18.3, CH_3_	22.0, CH_3_	13.7, CH_3_	57.4, CH_2_
18	27.8, CH_3_	19.0, CH_3_	18.9, CH_3_	18.9, CH_3_	11.4, CH_3_	27.8, CH_3_	185.1, C	28.5, CH_3_	65.3, CH_2_	28.7, CH_3_	28.4, CH_3_	21.9, CH_2_
19	108.2, CH_2_	59.2, CH_2_	59.2, CH_2_	59.4, CH_2_	59.7, CH_2_	108.4, CH_2_	30.1, CH_3_	16.0, CH_3_	23.6, CH_3_	16.7, CH_3_	16.0, CH_3_	33.4, CH_2_
20	14.2, CH_3_	15.6, CH_3_	15.4, CH_3_	18.3, CH_3_	25.9, CH_3_	15.8, CH_3_	15.6, CH_3_	17.4, CH_3_	20.4, CH_3_	17.9, CH_3_	18.4, CH_3_	18.4, CH_2_
1′	107.6, CH	107.6, CH	107.1, CH	101.5, CH	101.9, CH	102.4, CH	107.6, CH	107.4, CH	107.8, CH			
2′	75.4, CH	75.4, CH	75.4, CH	75.9, CH	75.7, CH	75.9, CH	75.4, CH	75.5, CH	75.6, CH			
3′	78.6, CH	78.6, CH	78.7, CH	78.3, CH	78.6, CH	78.4, CH	78.6, CH	79.1, CH	79.0, CH			
4′	70.8, CH	70.8, CH	70.7, CH	71.8, CH	71.4, CH	71.7, CH	70.8, CH	71.4, CH	71.5, CH			
5′	77.9, CH	77.9, CH	78.0, CH	78.1, CH	78.4, CH	78.2, CH	77.9, CH	77.9, CH	77.9, CH			
6′	62.1, CH_2_	62.1, CH_2_	62.1, CH_2_	62.6, CH_2_	62.3, CH_2_	62.6, CH_2_	62.1, CH_2_	63.0, CH_2_	63.0, CH_2_			

a In CD_3_OD solution.

b In acetone-*d*_6_ solution.

**Fig. 1:**
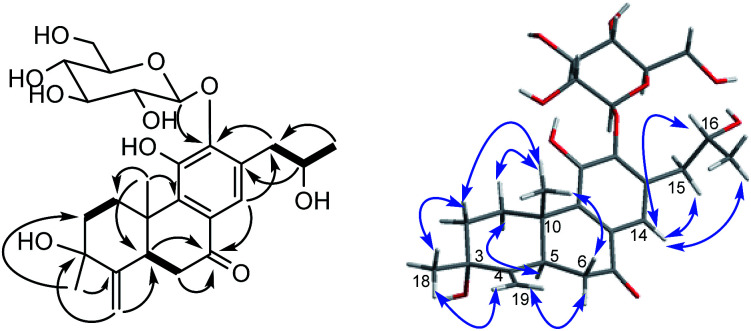
^1^H–^1^H COSY (bold), selected HMBC (arrow), and key ROESY (double arrow) correlations of 1.

The proton and protonated carbon NMR signals of 1 were assigned unambiguously by the HSQC experiment. Partial structures and the whole connection were deduced from correlations observed in the ^1^H–^1^H COSY and HMBC spectra (Fig. 1). The HMBC correlations of CH_3_-18 (*δ*_H_ 1.41) to C-2 (*δ*_C_ 37.8), C-3 (*δ*_C_ 71.5, an oxygenated quaternary carbon), and C-4 (*δ*_C_ 153.7, an olefinic quaternary carbon) and of H_2_-19 (*δ*_H_ 4.74, 5.20) to C-3/C-4/C-5 (*δ*_C_ 44.0) suggested the presence of a 18(4 → 3)-*abeo*-abietane structural unit, established the location of an OH group at C-3 and an exocyclic double bond at C-4 and C-19. The ketone group was placed at C-7 from correlations of H-5 (*δ*_H_ 3.32), H_2_-6 (*δ*_H_ 2.37, 2.69), H-14 (*δ*_H_ 7.46, one aromatic proton) to a ketone-group carbon (*δ*_C_ 200.7). ^1^H–^1^H COSY correlations from H_2_-15 (*δ*_H_ 2.71, 3.20) through H-16 (*δ*_H_ 4.10, an oxygenated proton) to CH_3_-17 (*δ*_H_ 1.12), in combination with HMBC correlations from H_2_-15 to C-12 (*δ*_C_ 150.5) and C-14 (*δ*_C_ 121.7), from CH_3_-17 to C-16 (*δ*_C_ 68.3) and C-15 (*δ*_C_ 40.9), were suggestive of 17(15 → 16)-*abeo*-abietane moiety in this structure, and an hydroxyl group at C-16. The location of the sugar moieties was determined by the HMBC correlation of the anomeric proton H-1′ (*δ*_H_ 4.57) to C-12. In addition, ^1^H–^1^H COSY correlations: H_2_-1/H_2_-2, H-5/H_2_-6, and HMBC correlations of CH_3_-20 to C-1, C-9 and C-10, of H-5 to C-4 and C-10 were also key interactions to support this gross structure.

**Fig. 2:**
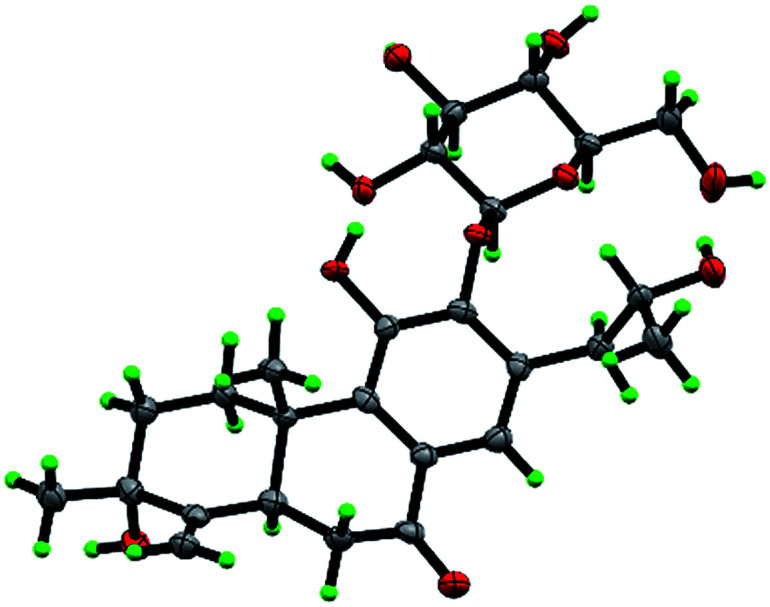
ORTEP plot for the molecular structure of 1 drawn with 30% probability displacement ellipsoids.

The β-d-configurational glucose unit was confirmed through coupling constant of H-1′ (d, *J* = 7.9), acid hydrolysis and comparison with reference standard. In the case of abietane diterpenoid derivatives, relative configuration of OH at C-3 could be assigned for the α- and β-epimers, by NOE effect between H-3/H-5 or H-3/CH_3_-20 respectively. However, the interaction of CH_3_-18/H-5 or CH_3_-18/CH_3_-20 was not observed in 1. Its ROESY spectrum provided interactions of CH_3_-18/H-2 and CH_3_-20/H-2 (Fig. 1), but α- and β-H connected to C-2 displayed an overlapped signal at 1.74 in the ^1^H NMR, which was not enough evidence to assign the C-3 configuration. Fortunately, appropriate crystals have been obtained, and the absolute configuration was assigned as 3*R*, 5*R*, 10*S*, 16*R* on the basis of the Flack parameter [0.17(14)] and Hooft parameter [0.10(6)] for 1225 Bijvoet pairs obtained by low-temperature [100(2) K] Cu Kα radiation X-ray crystallography (Fig. 2).^29,30^ Therefore, the structure of 1 was elucidated as (3*R*,16*R*)-12-*O*-β-d-glucopyranosyl-3,11,16-trihydroxy-17(15 → 16),18(4 → 3)-*diabeo*-4(19),8(9),11(12),13(14)-abietatetraene-7-one, named szemaoenoid A.

Compound 2 was isolated as a white amorphous powder with [*α*]^25^_D_ −8.8 (*c* 0.14, MeOH). The HRESIMS gave a quasi-molecular ion at *m*/*z* 531.2203 [M + Na]^+^ (calcd. for C_26_H_36_NaO_10_, 531.2206). Thus, in conjunction with ^13^C NMR and DEPT data, the molecular formula was established as C_26_H_36_O_10_, representing nine indices of hydrogen deficiency. The ^1^H and ^13^C NMR data of 2 (Tables 1 and 2) were similar to those of compound 1, with the differences being the presence of an oxygenated methylene [*δ*_H_ 4.07 (d, *J* = 11.7), 4.27 (d, *J* = 11.7), *δ*_C_ 59.2] and two olefinic quaternary carbons (*δ*_C_ 133.8, *δ*_C_ 129.6) in 2*vs.* an oxygenated quaternary carbon and an exocyclic double bond group in 1. Through HMBC experiment, observed correlations from H_2_-19 (*δ*_H_ 4.07, 4.27) to C-3/C-4/C-5, from CH_3_-18 (*δ*_H_ 1.26) to C-2/C-3/C-4 indicated that an OH group at C-19 and a double bond at C-3 and C-4. There was another hydroxyl group at C-16 (assigned the configuration as *R*) *via* comparing chemical shift and coupling constant of 2 with 1 [*δ*_H-16_ 4.11 (ddd, *J* = 6.2, 6.6, 6.7), *δ*_C-16_ 68.3 for 2; *δ*_H-16_ 4.10 (ddd, *J* = 6.2, 6.4, 6.6), *δ*_C-16_ 68.3 for 1], and the connecting correlations of ^1^H–^1^H COSY and HMBC data.

So far, the vast majority of natural abietane-type diterpenes from plants share the same carbon skeleton, with a *trans*-fused system of two six-membered rings A and B, a β-oriented methyl at C-10 and an α-oriented proton at C-5.^31^ From biogenetic considerations, 2 was inferred as possessing an identical absolute configuration to 1. Thus, the structure of 2 was established as (16*R*)-12-*O*-β-d-glucopyranosyl-11,16,19-trihydroxy-17(15 → 16),18(4 → 3)-*diabeo*-3(4),8(9),11(12),13(14)-abietatetraene-7-one, named szemaoenoid B.

**Fig. 3:**
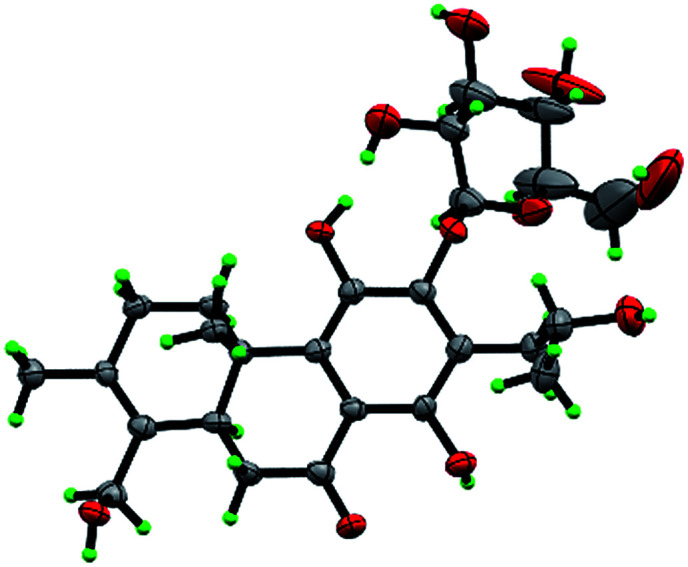
ORTEP plot for the molecular structure of 3 drawn with 30% probability displacement ellipsoids.

Compound 3 was obtained as white monoclinic crystals (MeOH). Its molecular formula assigned was determined to be C_26_H_36_O_11_ based on the negative HRESIMS (*m*/*z* 523.2177 [M − H]^−^). The NMR data for this compound were highly close to those of 2 (Tables 1 and 2), except for presence of one olefinic quaternary carbon (*δ*_C_ 157.4) in 3, correspondingly absence of an olefinic methine (*δ*_H_ 7.46, *δ*_C_ 122.1) *vs.*2. In the HMBC spectrum, the correlations of H_2_-15 (*δ*_H_ 2.87, 3.17) to C-12 (*δ*_C_ 153.3), C-13 (*δ*_C_ 121.0), C-14 (*δ*_C_ 157.4) indicated that an OH group was at C-14, which made the chemical shifts of aromatic fields and ketone group (*δ*_C_ 207.1) obviously change in ^13^C NMR spectrum *vs.*2. Ultimately, the absolute configuration was confirmed by single crystal X-ray diffraction analysis, which assigned C-16 as *R* configuration (Fig. 3). Therefore, the structure of 3 was determined as (16*R*)-12-*O*-β-d-glucopyranosyl-11,14,16,19-tetrahydroxy-17(15 → 16),18(4 → 3)-*diabeo*-3(4),8(9),11(12),13(14)-abietatetraene-7-one, named szemaoenoid C.

Compound 4, a yellowish amorphous powder, had a molecular formula of C_26_H_32_O_10_ according to HRESIMS (*m*/*z* 527.1888 [M + Na]^+^). The ^1^H NMR and ^13^C NMR data were closely related to those of 3 (Tables 1 and 2). The differences were emergence of an olefinic double bond: one methine at C-15 (*δ*_H_ 6.55, s; *δ*_C_ 101.5), one quaternary carbon at C-16 (*δ*_C_ 156.5). The HMBC correlations from CH_3_-17 (*δ*_H_ 2.46, s) to C-15/C-16 and from H-15 to C-12 (*δ*_C_ 153.4)/C-13 (*δ*_C_ 119.8)/C-14 (*δ*_C_ 156.8) powerfully confirmed the locations of the double bond between C-15 and C-16, and an OH group at C-16. In consideration of its molecular formula as C_26_H_32_O_10_, and chemical stability, this structure must be absence of one H_2_O, and linked from C-12 to C-16 through a vinyl ether bond, likewise the known compound 13 with a furan ring. The sugar moieties were determined to be located at C-11, by the HMBC correlation of the anomeric proton H-1′ (*δ*_H_ 5.41, d, 7.2) to C-11 (*δ*_C_ 133.7). Thus, compound 4 was established as 11-*O*-β-d-glucopyranosyl-12,16-epoxy-14,19-dihydroxy-17(15 → 16),18(4 → 3)-*diabeo*-3(4),8(9),11(12),13(14),15(16)-abietapentaene-7-one, named szemaoenoid D.

Compound 5 was obtained as a brown amorphous powder. Its molecular formula was determined to be C_26_H_28_O_11_ by HRESIMS data, indicating 12 degrees of unsaturation (two more than that of compound 4). The ^1^H and ^13^C NMR resonances of 5 closely resembled those of 4 (Tables 1 and 2), except for appearance of a ketone group at C-2 and a double bond at C-5/C-6. The HMBC correlations from H_2_-1 (*δ*_H_ 2.54, d, *J* = 16.9 and *δ*_H_ 4.29, d, *J* = 16.9) and CH_3_-18 (*δ*_H_ 2.06, s) to C-2 (*δ*_C_ 201.1) and from H-6 (*δ*_H_ 6.80, s) to C-4 (*δ*_C_ 149.6)/C-8 (*δ*_C_ 110.1)/C-10 (*δ*_C_ 44.2) demonstrated above inference. Therefore, the structure of 5 was elucidated as 11-*O*-β-d-glucopyranosyl-12,16-epoxy-14,19-dihydroxy-17(15 → 16),18(4 → 3)-*diabeo*-3(4),5(6),8(9),11(12),13(14),15(16)-abietahexaene-2,7-dione, named szemaoenoid E.

Compound 6 was isolated as a yellowish amorphous powder. The HRESIMS gave its molecular formula as C_26_H_32_O_10_ (being identical with 4). The NMR data of 6 (Tables 1 and 2) were similar to those of 4, except for an oxygenated quaternary carbon (*δ*_C_ 71.3) and an exocyclic double bond [*δ*_C_ 153.6, *δ*_C_ 108.4; *δ*_H_ 4.76, 5.20] in 6 (in accord with compound 1) *vs.* an oxygenated methylene and two olefinic quaternary carbons in 4. In the HMBC spectrum, the cross-connection signals of CH_3_-18 (*δ*_H_ 1.40) to C-2 (*δ*_C_ 38.1), C-3 (*δ*_C_ 71.3) and C-4 (*δ*_C_ 153.6), and of H_2_-19 [*δ*_H_ 4.76 (s), 5.20 (s)] to C-3, C-4 and C-5 (*δ*_C_ 43.7), supported the presence of an OH group at C-3 and a double bond at C-4/C-19. In consideration of identical NMR data and biogenesis, absolute configuration of C-3 in 6 was identical with 1 (assigned as *R* configuration). Consequently, compound 6 was established as (3*R*)-11-*O*-β-d-glucopyranosyl-12,16-epoxy-3,14-dihydroxy-17(15 → 16),18(4 → 3)-*diabeo*-4(19),8(9),11(12), 13(14),15(16)-abietapentaene-7-one, named szemaoenoid F.

Compound 7, a white amorphous powder, had a molecular formula of C_26_H_36_O_11_ on the basis of the HRESIMS. From the NMR data, it was similar to those of 2 (Tables 1 and 2), except for absence of two olefinic quaternary carbons (*δ*_C_ 133.8, 129.6) and an oxygenated methylene (*δ*_C_ 59.2) *vs.*2, correspondingly presence of an obvious carboxyl (*δ*_C_ 185.1), a quaternary carbon (*δ*_C_ 45.7) and a methylene (*δ*_C_ 39.9) in 7. The HMBC correlations of CH_3_-19 (*δ*_H_ 1.18, s) to C-3 (*δ*_C_ 39.9)/C-4 (*δ*_C_ 45.7)/C-5 (*δ*_C_ 53.6) and the carboxyl C-18 (*δ*_C_ 185.1), of H-5(*δ*_H_ 1.81, br d, *J* = 14.2) to C-4/C-18/CH_3_-19 (*δ*_C_ 30.1), and the ^1^H–^1^H COSY correlations of H_2_-1/H_2_-2/H_2_-3, all indicated that CH_3_-19 and the carboxyl C-18 were both linked to C-4. CH_3_-19 was assigned as β-orientation from the NOE effect of CH_3_-19/CH_3_-20. In view of identical NMR data and biogenesis, absolute configuration of C-16 in 7 was also identical with 1 (assigned as *R* configuration). Therefore, the structure of 7 was determined as (16*R*)-12-*O*-β-d-glucopyranosyl-11,16-dihydroxy-17(15 → 16)-*abeo*-8(9),11(12),13(14)-abietatriene-7-one-18-acid, named szemaoenoid G.

Compound 8, a white amorphous powder, exhibited a molecular formula of C_26_H_38_O_10_ according to HRESIMS. The ^1^H and ^13^C NMR spectra of 8 (Tables 1 and 2) were comparable with those of a known compound 12-*O*-d-glucopyranosyl-3,11,16-trihydroxy-8,11,13-abietatriene.^21^ The evident difference was appearance of carbonyl group (*δ*_C_ 201.3) in 8, but the known compound was absent of this group. The HMBC correlations of H-5 (*δ*_H_ 1.73)/H_2_-6 (*δ*_H_ 2.58, 2.66)/H-14 (*δ*_H_ 7.44) with C-7 (*δ*_C_ 201.3), of CH_3_-18 (*δ*_H_ 1.03)/CH_3_-19 (*δ*_H_ 0.93) with C-3 (*δ*_C_ 78.7), and ^1^H–^1^H COSY connections of H_2_-1/H_2_-2/H-3, suggested the carbonyl group at C-7 and an OH group at C-3, respectively. ^1^H–^1^H COSY correlations from CH_3_-17 (*δ*_H_ 1.15, d, *J* = 6.3) through H-15 (*δ*_H_ 3.77, m) to CH_2_-16 (*δ*_H_ 3.50, 3.61, an oxygenated methylene), and HMBC correlations from CH_3_-17 to C-13 (*δ*_C_ 138.2) and C-15 (*δ*_C_ 35.1), from H-15 to C-16 (*δ*_C_ 68.8), C-17 (*δ*_C_ 18.2), C-12 and C-14, were suggestive of an 1-hydroxy-isopropyl moiety linked to C-13 in this structure. Configurations of C-15 was established as *S*, by comparing the chemical shifts at C-13, C-15, C-16 with that of two known compounds (Table 3): (15*S*)-12-*O*-d-glucopy-ranosyl-3,11,16-trihydroxy-8,11,13-abie-tatriene^21^ and (15*R*)-cyrtophyllone B,^32^ whose structures were undoubtedly established by Mosher method and X-ray crystallography respectively. The β orientation of OH-3 was confirmed by the NOE effect of H-3/H-5. Therefore, this structure was established as (15*S*)-12-*O*-β-d-glucopyranosyl-3β,11,16-trihydroxy-8(9),11(12),13(14)-abietatriene-7-one, named szemaoenoid H.

**Table 3:** Comparison of partial NMR data of 8 and 9 with known compounds^a^

Position	*δ*_H_				Position	*δ*_C_			
	a	b	c	d		a	b	c	d
H-15	3.69	3.15	3.77	3.68	C-13	135.7	130	138.2	136.6
H-16a	3.42	3.78	3.50	3.42	C-15	35.1	39.1	35.1	34.9
H-16b	3.58	3.94	3.61	3.58					

a The a was (15*S*)-12-*O*-d-glucopyranosyl-3,11,16-trihydroxy-8,11,13-abietatriene;^21^b was (15*R*)-cyrtophyllone B;^32^c was szemaoenoid H (8); d was szemaoenoid I (9).

The HREIMS and NMR data of compound 9 were consistent with the molecular formula of C_26_H_40_O_10_. Its ^1^H and ^13^C NMR spectra (Tables 1 and 2) were almost identical with those of the known compound: 12-*O*-d-glucopyranosyl-3,11,16-trihydroxy-8,11,13-abietatriene.^21^ In fact, the only difference was an oxygenated methylene (*δ*_C_ 65.3) in 9 instead of a methyl group (*δ*_C_ 17.0) in the known structure. Moreover, the oxygenated methylene signals at *δ*_H_ 3.45 and 4.18 showed the HMBC correlations with the signals at C-3 (*δ*_C_ 80.9), C-4 (*δ*_C_ 44.3) and C-5 (*δ*_C_ 55.0), and the NOE effect with H-5/H-3, which evidently inferred that the OH was located at C-18, in combination with NOE effect of CH_3_-19/CH_3_-20. The β orientation of OH-3 and *S* configuration of C-15 were confirmed like that of compound 8. Consequently, compound 9 was assigned as (15*S*)-12-*O*-β-d-glucopyranosyl-3β,11,16,18-tetrahydroxy-8(9),11(12),13(14)-abietatriene, named szemaoenoid I.

**Fig. 4:**
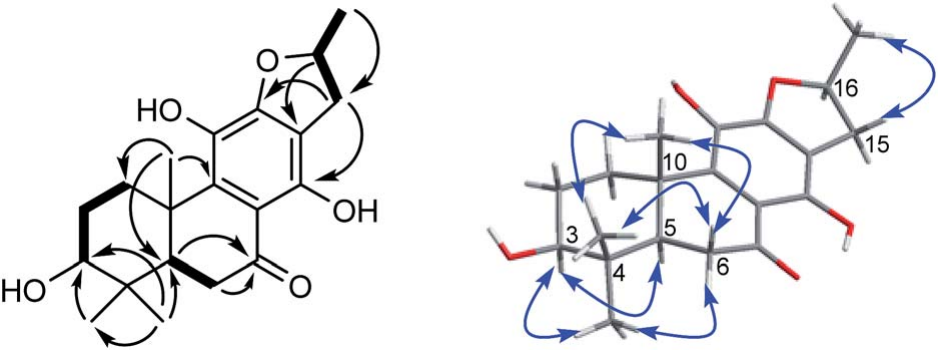
^1^H–^1^H COSY (bold), selected HMBC (arrow), and key ROESY (double arrow) correlations of 10.

**Fig. 5:**
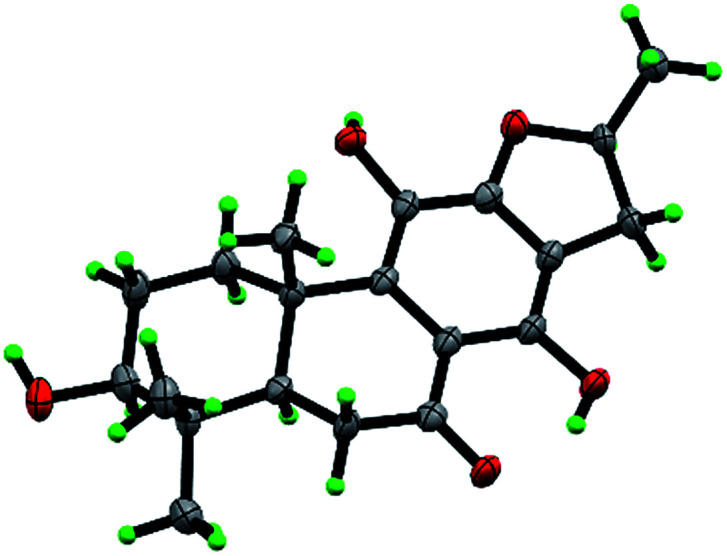
ORTEP plot for the molecular structure of 10 drawn with 30% probability displacement ellipsoids.

Compound 10, yellowish monoclinic crystals (MeOH), its chemical formula as C_20_H_26_O_5_ was determined by HRESIMS, indicating 8 degrees of unsaturation in the structure. From the ^1^H and ^13^C NMR spectrum (Tables 1 and 2), its data was extremely similar to known compound 14, with a 17(15 → 16)-*abeo*-abietane framework. Detailed HMBC and ^1^H–^1^H COSY NMR spectroscopic analyses (Fig. 4) suggested the appearance of an OH at C-3 [*δ*_H_ 3.27; *δ*_C_ 77.5] and the absence of a hydroxy group at C-17 in 10 by comparing with 14. A correlation observed in the ROESY spectrum (Fig. 4) between H-3 and H-5 (*δ*_H_ 1.71) indicated the β-orientation of OH-3. The absolute configuration of this compound was established as 3*S*, 5*R*, 10*S*, 16*S* by single crystal X-ray diffraction analysis (Fig. 5). Accordingly, the structure of 10 was assigned as (3*S*,16*S*)-12,16-epoxy-3,11,14-trihydroxy-17(15 → 16)-*abeo*-8(9),11(12),13(14)-abietatriene-7-one, named szemaoenoid J.

Compound 11 and 12 were assigned to an identical molecular formula of C_20_H_24_O_5_ by HRESIMS data, with one more degree of unsaturation then 10. As seen from ^1^H NMR and ^13^C NMR spectrum (Tables 1 and 2), the structures of 11 and 12 were highly analogous to compound 10, except for the appearance of a double bond between C-15 and C-16 in 11 and 12, besides the presence of oxygenated methylene at C-17 [*δ*_H_ 4.64, d, *J* = 5.9; *δ*_C_ 57.4] and the absence of OH-3 in 12. The positions of all functional groups in their structures were assigned by correlations of HMBC and ^1^H–^1^H COSY spectrum. The β-orientation of OH-3 in 11 was confirmed by ROESY correlation of H-3/H-5 and comparing with NMR data with those of 10. Therefore, their structures were established as 12,16-epoxy-3β,11,14-trihydroxyl-17(15 → 16)-*abeo*-8(9),11(12),13(14),15(16)-abietatetraene-7-one (11), named szemaoenoid K; and 12,16-epoxy-11,14,17-trihydroxy-17(15 → 16)-*abeo*-8(9),11(12), 13(14),15(16)-abietatetraene-7-one (12), named szemaoenoid L.

In our cognition, many diterpenoides had been reported from plants of *Premna* genus,^33–36^ but this was also the first to report diterpenoids isolated from the *Premna* plants distributed in China, and the described rearranged-abietane skeletons were firstly isolated from *Premna* genus. Abietane diterpenoids represented a large group of secondary metabolites that have shown interesting biological activities.^31^ But the rearranged abietane diterpenoids with 17(15 → 16)-*abeo*-abietane or 17(15 → 16),18(4 → 3)-*diabeo*-abietane were not common in nature. Structurally, the rearranged diterpenoids contain abundant hydroxyl groups and aromatic carbons, as well as *trans*-fused rings A and B according to biosynthetic pathway. However, the stereocenter of C-16 replaced with OH had never been established previously. Within this work, we firstly employed X-ray crystallography to assign absolute configuration of C-16 for a small series of 16-hydroxy-17(15 → 16)-*abeo*-abietane diterpenoids, which might assist future unambiguous identification of structurally related compounds.

All the above compounds, except 3, 4 and 10, were evaluated for cytotoxicity *in vitro* against two human colon carcinoma cell lines (HCT-116 and HT-29). As a result (Table 4), compounds 11–15 showed antiproliferative activity against HCT-116 cell line, and compounds 11, 12 and 15 also exhibited potent cytotoxicity on HT-29 colon carcinoma cell line. Impressively, the antiproliferation activity of compound 14 was comparable (IC_50_ 8.7 ± 1.4 μM) with positive control (sorafenib, IC_50_ 8.4 ± 1.3 μM) (Table 4). Almost all the diterpene aglycones showed effective cytotoxicity, but none of the diterpene glucosides exhibited remarkable activity. The reason might be that the glucosides with strong chemical polarity failed to penetrate the liposoluble cell membrane.

**Table 4:** IC_50_ values (μM ± SD) obtained for the compounds against HCT-116 and HT-29 cell lines^a^

Compound	IC_50_ (mean ± SD, μM)	
	HCT-116	HT-29
11	30.5 ± 4.7	21.3 ± 2.9
12	24.1 ± 4.5	34.6 ± 3.4
13	34.3 ± 2.8	NA
14	17.7 ± 4.6	8.7 ± 1.4
15	20.1 ± 3.0	14.2 ± 2.6
Sorafenib	8.5 ± 1.1	8.4 ± 1.3

a NA means that compounds exhibited indistinctive activity against tumor cells, and IC_50_ values were not evaluated.

As most of natural products possessing phenolic hydroxy exhibited antioxidant activity,^37–39^ some selective compounds of the diterpenosides were executed free radical scavenging activity assay in the DPPH experiment (Table 5). Among the tested compounds, diterpene aglycones 13, 14 showed strong free radical scavenging activity with IC_50_ values of 41.5 ± 17.0 and 39.9 ± 12.9 μM respectively, and compound 10 was especially the strongest activity with IC_50_ 35.6 ± 9.8 μM (more potent than the positive control trolox and vitamin C). Compound 12 exhibited slightly weaken antioxidant activity with IC_50_ values of 74.9 ± 6.9 μM. None of the tested diterpene glucosides (1, 2, 3, 5, 9 and 16) showed potent free radical scavenging activity. Concerning the structure–activity relationship, a reasonable conclusion was reasoned out that the more the structure possessed phenolic hydroxyl groups, the more its scavenging activity was strong.

**Table 5:** DPPH free radical scavenging activity for the compounds^a^

Compound	IC_50_ (mean ± SD, μM)
1	>200
2	>200
3	>200
5	149.5 ± 51.1
9	>200
10	35.6 ± 9.8
12	74.9 ± 6.9
13	41.5 ± 17.0
14	39.9 ± 12.9
16	>200
Trolox	36.3 ± 5.6
Ascorbic acid (vitamin C)	39.1 ± 6.7

a IC_50_, 50% inhibitory concentration. Mean activity of IC_50_ was exhibited by mean ± standard deviation, *n* ≥ 3.

## Experimental section

### General experimental procedures

X-ray data were collected using a Bruker APEX DUO instrument. Optical rotations were measured with Horiba SEPA-300 and JASCO P-1020 polarimeters. UV spectra were recorded on a Shimadzu UV-2401A spectrophotometer. IR spectra were obtained on a Tenor 27 spectrophotometer with KBr pellets. One-dimensional (1D) and two-dimensional (2D) NMR spectra were recorded on Bruker DRX-600 spectrometers with TMS as the internal standard. Chemical shifts (*δ*) were expressed in parts per million with reference to the solvent signals. HRESIMS was performed on an Agilent G6230 TOF MS. Semi-preparative HPLC was performed on an Agilent 1260 liquid chromatograph with a Zorbax SB-C18 (9.4 mm × 25 cm) column. Column chromatography (CC) was performed on silica gel (100–200 mesh and 200–300 mesh; Qingdao Marine Chemical Inc., Qingdao, People's Republic of China), Lichroprep RP-18 gel (40–63 μm, Merck, Darmstadt, Germany), MCI gel (75–150 μm, Mitsubishi Chemical Corporation, Tokyo, Japan), and Sephadex LH-20 (Pharmacia). Fractions were monitored by TLC, and spots were visualized by UV light (254 nm) and sprayed with 8% H_2_SO_4_ in ethanol, followed by heating.

### Plant materials

Aerial parts of *Premna szemaoensis* were collected in February 2012 from Puer city, Yunnan Province, People's Republic of China, and identified by Researcher Xi-Wen Li, Kunming Institute of Botany. A voucher specimen (XWL20140403) has been deposited in the Herbarium of the Kunming Institute of Botany, Chinese Academy of Sciences.

### Extraction and isolation

The air-dried and powdered aerial parts of *P. szemaoensis* (10 kg) were extracted with 70% aqueous acetone (40 L) four times (two days each time) at room temperature and then filtered. The filtrate was evaporated under reduced pressure at 40 °C and then partitioned between *n*-butanol and H_2_O. The *n*-butyl alcohol soluble portion (600 g) was subjected to silica gel CC (2.5 kg, 100–200 mesh), eluted with a CHCl_3_–Me_2_CO gradient system (1:0–0:1) that afforded fractions A–E. The fractions were then decolorized using MCI gel and eluted with 95% MeOH–H_2_O.

Fraction B (33 g) was subjected to silica gel CC (200–300 mesh), eluted with a CHCl_3_–MeOH gradient (150:1–1:1), to yield fractions B1–B5. Fraction B1 was purified by repeated silica gel CC (petroleum ether–Me_2_CO gradient, 12:1–0:1) to yield compound 14 (10.2 mg). Fraction B3 was purified by Sephadex LH-20 (MeOH) to yield fractions B31–B34. Then compound 13 (75.0 mg) was crystallized from fraction B34, and compound 15 (8.4 mg) was isolated by HPLC (78% MeOH–H_2_O, *R*_t_ = 15.2 min). B4 was purified by RP-18 CC (MeOH–H_2_O gradient, 30–100%) to yield fractions B41–B45, then B43 was isolated by semi-preparative HPLC (72% MeOH–H_2_O, *R*_t_ = 13.5 min) to obtain compound 10 (10.1 mg). B5 was subjected to Sephadex LH-20 CC (MeOH) to give B51–B55, then compounds 11 (8.7 mg) and 12 (15.4 mg) were isolated from fraction B52 by semi-preparative HPLC (75% MeOH–H_2_O, *R*_t_ = 13.1 and 14.8 min, respectively).

Fraction C (120 g) was separated by Sephadex LH-20 (MeOH) to give fractions C1–C5. Fraction C2 was subjected to repeated silica gel CC (200–300 mesh), eluted with CHCl_3_–MeOH (gradient system: 120:1–1:1) to yield fractions C21–C27. C23 was isolated by semi-preparative HPLC (42% MeOH–H_2_O, *R*_t_ = 15.1 and 13.2 min, respectively) to afford compounds 1 (8.5 mg) and 2 (11.7 mg). C25 was purified by HPLC (44% MeOH–H_2_O, *R*_t_ = 10.3, 15.3 and 14.5 min, respectively) to yield compounds 4 (5.2 mg), 5 (4.6 mg), and 6 (2.3 mg).

Fraction D (100 g) was subjected to RP-18 CC (MeOH–H_2_O, 10–100%) to give fractions D1–D6. Compound 16 (20.1 mg) was crystallized from fractions D2. Fraction D4 was separated to by Sephadex LH-20 eluted with MeOH to give D41–D47, then D43 was isolated by semi-preparative HPLC (41% MeOH–H_2_O, *R*_t_ = 14.5 and 7.8 min, respectively) to yield compounds 3 (6.1 mg) and 7 (4.3 mg). Fraction D5 was separated to silica gel CC (200–300 mesh) eluted with CHCl_3_–MeOH (50:1–1:2) to yield D51–D56, then D54 was followed by HPLC (44% MeOH–H_2_O, *R*_t_ = 15.5 and 14.2 min, respectively) to afford compounds 8 (4.6 mg) and 9 (2.3 mg).

#### Szemaoenoid A (1)

White monoclinic crystals (MeOH); mp 257–259 °C (MeOH); [*α*]^22^_D_ +36.3 (*c* 0.10, MeOH); UV (MeOH) *λ*_max_ (log *ε*) 213 (4.23), 269 (3.91), 318 (3.49) nm; IR (KBr) *ν*_max_ 3441, 1632, 1049 cm^−1^; ^1^H and ^13^C NMR data, see Tables 1 and 2; positive-ion ESIMS *m*/*z* 531 [M + Na]^+^; positive-ion HRESIMS [M + Na]^+^*m*/*z* 531.2205 (calcd for 531.2206).

#### Szemaoenoid B (2)

White amorphous powder; [*α*]^22^_D_ +28.8 (*c* 0.14, MeOH); UV (MeOH) *λ*_max_ (log *ε*) 204 (4.17), 270 (3.96), 318 (3.32) nm; IR (KBr) *ν*_max_ 3441, 1632, 1071 cm^−1^; ^1^H and ^13^C NMR data, see Tables 1 and 2; positive-ion ESIMS *m*/*z* 531 [M + Na]^+^; positive-ion HRESIMS [M + Na]^+^*m*/*z* 531.2203 (calcd for 531.2206).

#### Szemaoenoid C (3)

White monoclinic crystals (MeOH); mp 275–278 °C (MeOH); [*α*]^22^_D_ +17.8 (*c* 0.12, MeOH); UV (MeOH) *λ*_max_ (log *ε*) 203 (4.55), 239 (1.55), 283 (1.51) nm; IR (KBr) *ν*_max_ 3414, 1616, 1424, 1072 cm^−1^; ^1^H and ^13^C NMR data, see Tables 1 and 2; negative-ion ESIMS *m*/*z* 523 [M − H]^−^; negative-ion HRESIMS [M − H]^−^*m*/*z* 523.2177 (calcd for 523.2179).

#### Szemaoenoid D (4)

Yellowish amorphous powder; [*α*]^22^_D_ −54.1 (*c* 0.04, MeOH); UV (MeOH) *λ*_max_ (log *ε*) 204 (4.47), 239(4.41), 254 (4.33), 355 (3.61) nm; IR (KBr) *ν*_max_ 3425, 2924, 1631, 1384, 1069, 586 cm^−1^; ^1^H and ^13^C NMR data, see Tables 1 and 2; positive-ion ESIMS *m*/*z* 527 [M + Na]^+^; positive-ion HRESIMS [M + Na]^+^*m*/*z* 527.1888 (calcd for 527.1893).

#### Szemaoenoid E (5)

Brown amorphous powder; [*α*]^22^_D_ −53.0 (*c* 0.10, MeOH); UV (MeOH) *λ*_max_ (log *ε*) 202 (4.13), 295 (4.06) nm; IR (KBr) *ν*_max_ 3426, 1629, 1466, 1216, 580 cm^−1^; ^1^H and ^13^C NMR data, see Tables 1 and 2; positive-ion ESIMS *m*/*z* 539 [M + Na]^+^; positive-ion HRESIMS [M + Na]^+^*m*/*z* 539.1519 (calcd for 539.1529).

#### Szemaoenoid F (6)

Yellowish amorphous powder; [*α*]^22^_D_ +78.8 (*c* 0.06, MeOH); UV (MeOH) *λ*_max_ (log *ε*) 202 (4.27), 240 (4.34), 254 (4.27), 356 (3.52) nm; IR (KBr) *ν*_max_ 3422, 2924, 1635, 1357, 1201, 1077 cm^−1^; ^1^H and ^13^C NMR data, see Tables 1 and 2; negative-ion ESIMS *m*/*z* 503 [M − H]^−^; negative-ion HREIMS [M − H]^−^*m*/*z* 503.1912 (calcd for 503.1917).

#### Szemaoenoid G (7)

White amorphous powder; [*α*]^22^_D_ +15.9 (*c* 0.13, MeOH); UV (MeOH) *λ*_max_ (log *ε*) 215 (4.25), 269 (3.84), 316 (3.44) nm; IR (KBr) *ν*_max_ 3441, 1632, 1068 cm^−1^; ^1^H and ^13^C NMR data, see Tables 1 and 2; negative-ion ESIMS *m*/*z* 523 [M − H]^−^; negative-ion HRESIMS [M − H]^−^*m*/*z* 523.2176 (calcd for 523.2179).

#### Szemaoenoid H (8)

White amorphous powder; [*α*]^22^_D_ −8.57 (*c* 0.07, MeOH); UV (MeOH) *λ*_max_ (log *ε*) 215 (4.11), 269 (3.76), 316 (3.33) nm; IR (KBr) *ν*_max_ 3428, 1632, 1069 cm^−1^; ^1^H and ^13^C NMR data, see Tables 1 and 2; negative-ion ESIMS *m*/*z* 509 [M − H]^−^; negative-ion HRESIMS [M − H]^−^*m*/*z* 509.2388 (calcd for 509.2387).

#### Szemaoenoid I (9)

White amorphous powder; [*α*]^22^_D_ +9.25 (*c* 0.09, MeOH); UV (MeOH) *λ*_max_ (log *ε*) 204 (4.65), 275 (3.27) nm; IR (KBr) *ν*_max_ 3424, 1632, 1422, 1063, 596 cm^−1^; ^1^H and ^13^C NMR data, see Tables 1 and 2; negative-ion ESIMS *m*/*z* 511 [M − H]^−^; negative-ion HRESIMS [M − H]^−^*m*/*z* 511.2540 (calcd for 511.2543).

#### Szemaoenoid J (10)

Yellowish monoclinic crystals (MeOH); mp 213–215 °C (MeOH); [*α*]^22^_D_ +30.56 (*c* 0.12, MeOH); UV (MeOH) *λ*_max_ (log *ε*) 203 (4.84), 212 (4.70), 298 (3.10), 353 (1.67) nm; IR (KBr) *ν*_max_ 3427, 1632, 1462, 1351, 1015 cm^−1^; ^1^H and ^13^C NMR data, see Tables 1 and 2; negative-ion ESIMS *m*/*z* 345 [M − H]^−^; negative-ion HRESIMS [M − H]^−^*m*/*z* 345.1708 (calcd for 345.1702).

#### Szemaoenoid K (11)

Yellowish amorphous powder; [*α*]^22^_D_ +61.3 (*c* 0.10, MeOH); UV (MeOH) *λ*_max_ (log *ε*) 202 (4.23), 237 (4.25), 258 (4.18), 369 (3.52) nm; IR (KBr) *ν*_max_ 3441, 1631, 1458, 1372, 1277, 1179, 1069, 602 cm^−1^; ^1^H and ^13^C NMR data, see Tables 1 and 2; negative-ion ESIMS *m*/*z* 343 [M − H]^−^; negative-ion HRESIMS [M − H]^−^*m*/*z* 343.1551 (calcd for 343.1546).

#### Szemaoenoid L (12)

Yellowish amorphous powder; [*α*]^22^_D_ +43.3 (*c* 0.11, MeOH); UV (MeOH) *λ*_max_ (log *ε*) 202 (3.99), 237 (4.06), 260 (4.02), 366 (3.36) nm; IR (KBr) *ν*_max_ 3429, 2926, 1631, 1456, 1370 cm^−1^; ^1^H and ^13^C NMR data, see Tables 1 and 2; negative-ion ESIMS *m*/*z* 343 [M − H]^−^; negative-ion HRESIMS [M − H]^−^*m*/*z* 343.1548 (calcd for 343.1546).

### X-ray crystal structure analysis

Crystals of 1, 3 and 10 were obtained in MeOH, respectively. Intensity data were collected at 100 K on a Bruker APEX DUO diffractometer equipped with an APEX II CCD using Cu K*α* radiation. Cell refinement and data reduction were performed with Bruker SAINT. The structures were solved by direct methods using SHELXS-97.^40^ Refinements were performed with SHELXL-97 and SHELXL-2014 using full-matrix least-squares, with anisotropic displacement parameters for all the nonhydrogen atoms. The H-atoms were placed in calculated positions and refined using a riding model. Molecular graphics were computed with PLATON.^41^ Crystallographic data (excluding structure factor tables) for the structures reported have been deposited with the Cambridge Crystallographic Data Center as supplementary publications no. CCDC 1554050 for 1, CCDC 1554052 for 3, and CCDC 1554051 for 10.†

#### Crystal data for szemaoenoid A (1)

C_26_H_36_O_10_·H_2_O, *M* = 526.56, monoclinic, *a* = 5.70480(10) Å, *b* = 23.8602(5) Å, *c* = 9.3419(2) Å, *α* = 90.00°, *β* = 90.6040(10)°, *γ* = 90.00°, *V* = 1271.53(4) Å^3^, *T* = 100(2) K, space group *P*2_1_, *Z* = 2, *μ*(CuKα) = 0.898 mm^−1^, 10 232 reflections measured, 3499 independent reflections (*R*_int_ = 0.0328). The final *R*_1_ values were 0.0300 (*I* > 2*σ*(*I*)). The final w*R*(*F*^2^) values were 0.0884 (*I* > 2*σ*(*I*)). The final *R*_1_ values were 0.0300 (all data). The final w*R*(*F*^2^) values were 0.0885 (all data). The goodness of fit on *F*^2^ was 1.113. Flack parameter = 0.17(14).^29^ The Hooft parameter is 0.10(6) for 1225 Bijvoet pairs.^30^

#### Crystal data for szemaoenoid C (3)

4(C_26_H_36_O_11_)·H_2_O, *M* = 2116.20, *a* = 17.5477(6) Å, *b* = 21.7199(7) Å, *c* = 33.3592(12) Å, *α* = 90°, *β* = 90°, *γ* = 90°, *V* = 12 714.3(8) Å^3^, *T* = 100(2) K, space group *P*2_1_2_1_2_1_, *Z* = 4, *μ*(CuKα) = 0.728 mm^−1^, 112641 reflections measured, 23 429 independent reflections (*R*_int_ = 0.0484). The final *R*_1_ values were 0.0744 (*I* > 2*σ*(*I*)). The final w*R*(*F*^2^) values were 0.2065 (*I* > 2*σ*(*I*)). The final *R*_1_ values were 0.0759 (all data). The final w*R*(*F*^2^) values were 0.2083 (all data). The goodness of fit on *F*^2^ was 1.044. Flack parameter = 0.11(3).^42^

#### Crystal data for szemaoenoid J (10)

C_20_H_26_O_5_, *M* = 346.41, monoclinic, *a* = 11.5843(7) Å, *b* = 9.5501(6) Å, *c* = 15.2093(10) Å, *α* = 90.00°, *β* = 92.859(4)°, *γ* = 90.00°, *V* = 1680.53(18) Å^3^, *T* = 100(2) K, space group *P*2_1_, *Z* = 4, *μ*(CuKα) = 0.794 mm^−1^, 10 384 reflections measured, 4801 independent reflections (*R*_int_ = 0.0536). The final *R*_1_ values were 0.0627 (*I* > 2*σ*(*I*)). The final w*R*(*F*^2^) values were 0.1682 (*I* > 2*σ*(*I*)). The final *R*_1_ values were 0.0701 (all data). The final w*R*(*F*^2^) values were 0.1743 (all data). The goodness of fit on *F*^2^ was 1.058. Flack parameter = 0.0(2).^29^ The Hooft parameter is 0.01(14) for 1718 Bijvoet pairs.^30^

### Acid hydrolysis of szemaoenoid A

Compound 1 (4 mg) was hydrolyzed with 2 M HCl/dioxane (1 : 1, 4 mL) under reflux for 8 h, respectively. The reaction mixture was partitioned between H_2_O and CHCl_3_ (2 mL × 3). The aqueous layer was neutralized with 2 M NaOH and then dried to give a monosaccharide. A solution of the sugar in pyridine (2 mL) was added to l-cysteine methyl ester hydrochloride (about 1.0 mg) and kept at 60 °C for 1 h. Then trimethylsilylimidazole (about 1.0 mL) was added to the reaction mixture and kept at 60 °C for 30 min. The mixture was subjected to GC analysis, run on a Shimadzu GC-14C gas chromatograph equipped with an H_2_ flame ionization detector. The column was a 30 m × 0.32 mm i.d. 30QC2/AC-5 quartz capillary column with the following conditions: column temperature, 180–280 °C; programmed increase, 3 °C min^−1^; carrier gas, N_2_ (1 mL min^−1^); injector and detector temperature, 250 °C; injection volume, 4 μL; and split ratio, 1/50. The configuration of the sugar moiety was determined by comparing the retention time with the derivatives of the authentic samples. The retention times of d-/l-glucose were 21.115/21.565 min.^43^ The configuration of the sugar moiety from compound 1 was d-glucose (*R*_t_ = 21.117 min).

### Cytotoxicity assay

Human colon adenocarcinoma cell lines, HCT-116 and HT-29 were obtained from the American Type Culture Collection (ATCC). The cells were cultured in Dulbecco's modified Eagle's medium (DMEM) supplemented with 10% FBS in a 5% CO_2_ atmosphere. HCT-116 (3 × 10^3^ per well) and HT-29 (6 × 10^3^ per well) were seeded onto 96-well plates and allowed to grow for 24 h prior to treatment. Different concentrations of compounds were then added and further incubated for 3 days. Sorafenib (purity > 99%; Medchem Express) was used as positive control. The culture medium was replaced by fresh DMEM containing 0.5 mg mL^−1^ of MTT. After incubation for another 4 h, the medium was removed and the reduced formazan blue was solubilized by adding 100 μL DMSO to each well. The absorbance at 492 nm was measured using a microplate reader (Multiskan MK3, Thermo). The IC_50_ values were calculated from concentration–response curves using Graphpad Prism software.

### Antioxidant activity assay

Trolox (purity > 98%; Sigma) and vitamin C (Ascorbic acid, purity > 98%; Sigma) were used as positive control. A 0.1 mM solution of DPPH radical in ethanol was prepared, and 100 μL of this solution was mixed with 100 μL of sample solution. The mixture was incubated for 5 min in a dark room at room temperature. Scavenging capacity was read spectropho-tometrically by monitoring the decrease in absorbance at 517 nm. DPPH scavenging activity (%) = [1 − (*S* − B)/(*C* − B)] × 100%, where *S*, *B* and *C* are the absorbencies of the sample, the blank and the control, respectively.^44^

## Conclusions

In summary, we have firstly reported twelve new abietane diterpenoids (1–12) isolated from *P. szemaoensis*, together with four known compounds (13–16). Structurally, these compounds involved two rearranged-abietane skeletons: 17(15 → 16)-*abeo*-abietane and 17(15 → 16),18(4 → 3)-*diabeo*-abietane. Their structures with absolute configurations were characterized by a series of spectroscopic methods and X-ray diffraction. In bioactivity assays, compounds 11, 12, 14 and 15 were active against two human colon cancer cell lines (HCT-116 and HT-29) with IC_50_ values ranging from 8.8 to 34.3 μM, and compounds 10, 13 and 14 exhibited effective free radical scavenging activity with IC_50_ values ranging from 35.6 to 41.5 μM by DPPH experiment. In short, the current study adds to understanding of the chemical composition and biological effects of this plant prepared for green food and ethnodrugs.

## Conflicts of interest

There are no conflicts to declare.

## Supplementary Material

RA-008-C7RA13309J-s001

RA-008-C7RA13309J-s002
