# Supplementary material for: Isolation, identification and bioactivities of abietane diterpenoids from Premna szemaoensis
Source: RSC Adv. 2018 Feb 9;8(12):6425–35. doi: 10.1039/c7ra13309j (PMC9078283; doi:10.1039/c7ra13309j)
Supplement: RA-008-C7RA13309J-s001 [file RA-008-C7RA13309J-s001.pdf]

Electronic Supplementary Material (ESI) for RSC Advances.
This journal is © The Royal Society of Chemistry 2018

Isolation, Identification and Bioactivities of Abietane Diterpenoids from Premna szemaoensis

De-Bing Pu[†,§,#,⊥], Ting Wang[‡,⊥], Xing-Jie Zhang[†], Jun-Bo Gao[§,#], Rui-Han Zhang[†], Xiao-Nian Li[§], Yong-Mei Wang[§,#], Xiao-Li Li[†,*], He-Yao Wang[‡,*], Wei-Lie Xiao[†,§,*]

[†]*Key Laboratory of Medicinal Chemistry for Natural Resource of Ministry of Education, School of Chemical Science and Technology, and State Key Laboratory for Conservation and Utilization of Bio-Resources in Yunnan, Yunnan University, Kunming 650091, People's Republic of China*

[‡]*State Key Laboratory of Drug Research, Shanghai Institute of Materia Medica, Chinese Academy of Sciences, Shanghai 201203, People's Republic of China*

[§]*State Key Laboratory of Phytochemistry and Plant Resources in West China, Kunming Institute of Botany, Chinese Academy of Sciences, Kunming 650201, People's Republic of China*

[#]*University of Chinese Academy of Sciences, Beijing 100049, People's Republic of China*

Corresponding author contact detail:

*E-mail: lixiaoli@ynu.edu.cn. Tel: (86) 871-67357014.

*E-mail: hywang@simm.ac.cn. Tel: (86) 021-50805785.

*E-mail: xiaoweilie@ynu.edu.cn. Tel: (86) 871-67357014.

Contents

Figure 1S-9S. NMR, MS, UV, and IR spectra of compound **1**.................................3

Figure 10S-18S. NMR, MS, UV, and IR spectra of compound **2**.............................8

Figure 19S-27S. NMR, MS, UV, and IR spectra of compound **3**............................13

Figure 28S-36S. NMR, MS, UV, and IR spectra of compound **4**............................18

Figure 37S-45S. NMR, MS, UV, and IR spectra of compound **5**............................23

Figure 46S-54S. NMR, MS, UV, and IR spectra of compound **6**............................28

Figure 55S-63S. NMR, MS, UV, and IR spectra of compound **7**............................33

Figure 64S-72S. NMR, MS, UV, and IR spectra of compound **8**............................38

Figure 73S-81S. NMR, MS, UV, and IR spectra of compound **9**............................43

Figure 82S-90S. NMR, MS, UV, and IR spectra of compound **10**..........................48

Figure 91S-99S. NMR, MS, UV, and IR spectra of compound **11**..........................53

Figure 100S-108S. NMR, MS, UV, and IR spectra of compound **12**......................58

Figure 109S. The pack drawing of compound **1** 63

Figure 110S. The pack drawing of compound **3** 64

Figure 111S. The pack drawing compound **10** 65

Table 1S. Crystal data and structure refinement for **1**..66

Table 2S. Crystal data and structure refinement for **3**..67

Table 3S. Crystal data and structure refinement for **10**..68

Figure 1S-9S. NMR, MS, UV, and IR spectra of compound 1

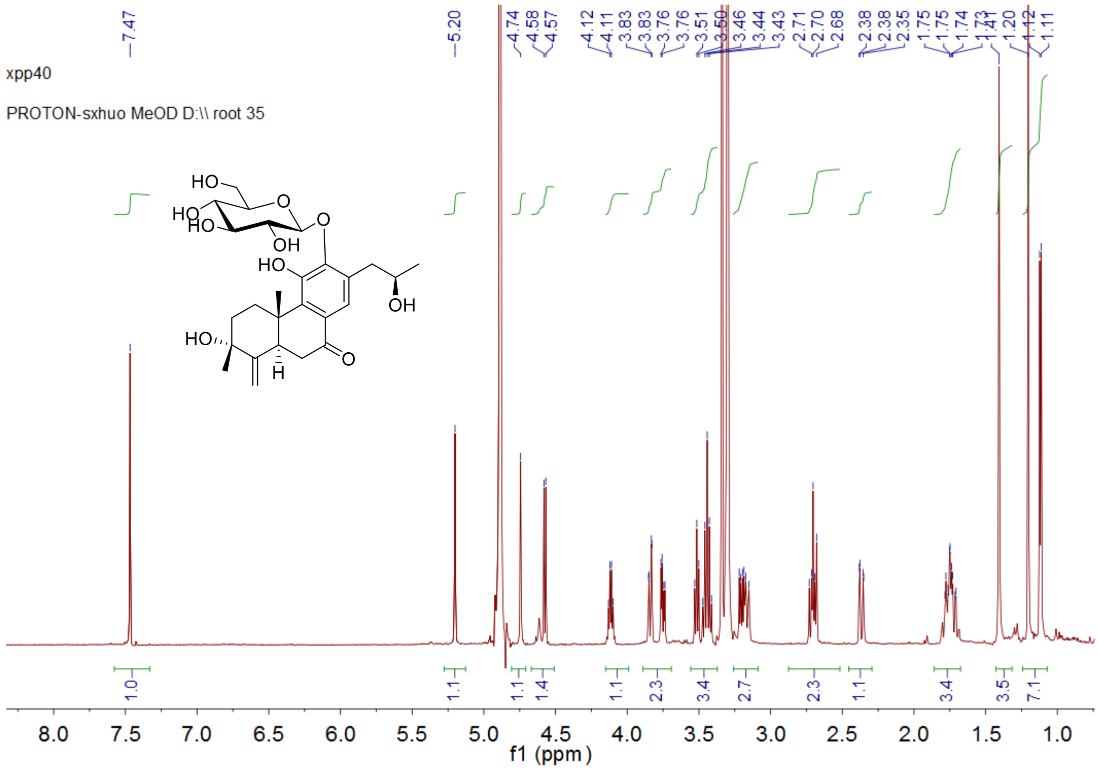

Figure 1S. ^{1}H NMR spectrum of (**1**) recorded in CD_3OD at 600 MHz

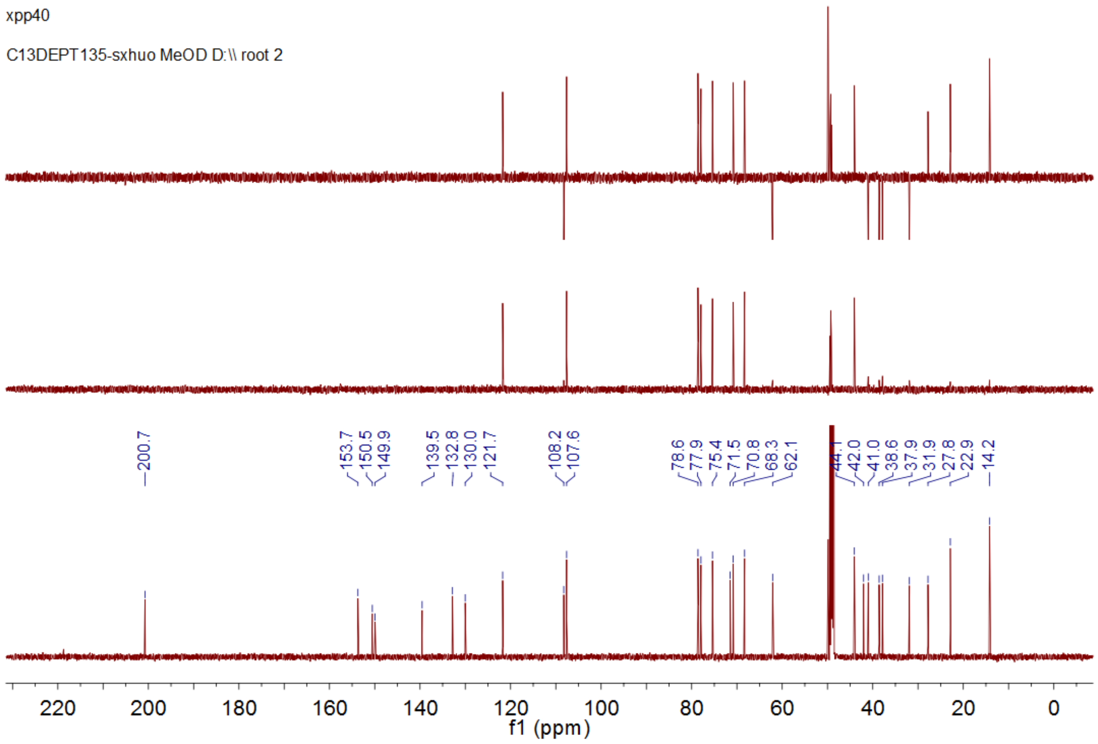

Figure 2S. ^{13}C NMR spectrum of (**1**) recorded in CD_3OD at 150 MHz

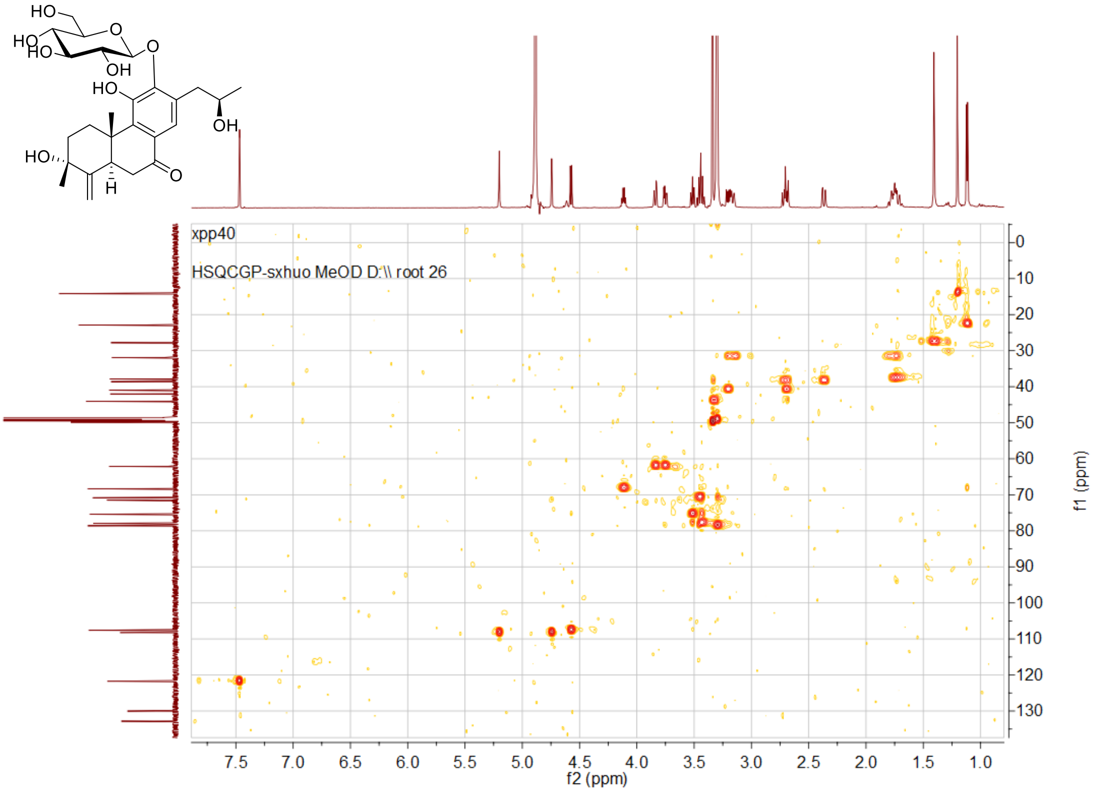

Figure 3S. HSQC spectrum of (**1**) recorded in CD_3OD

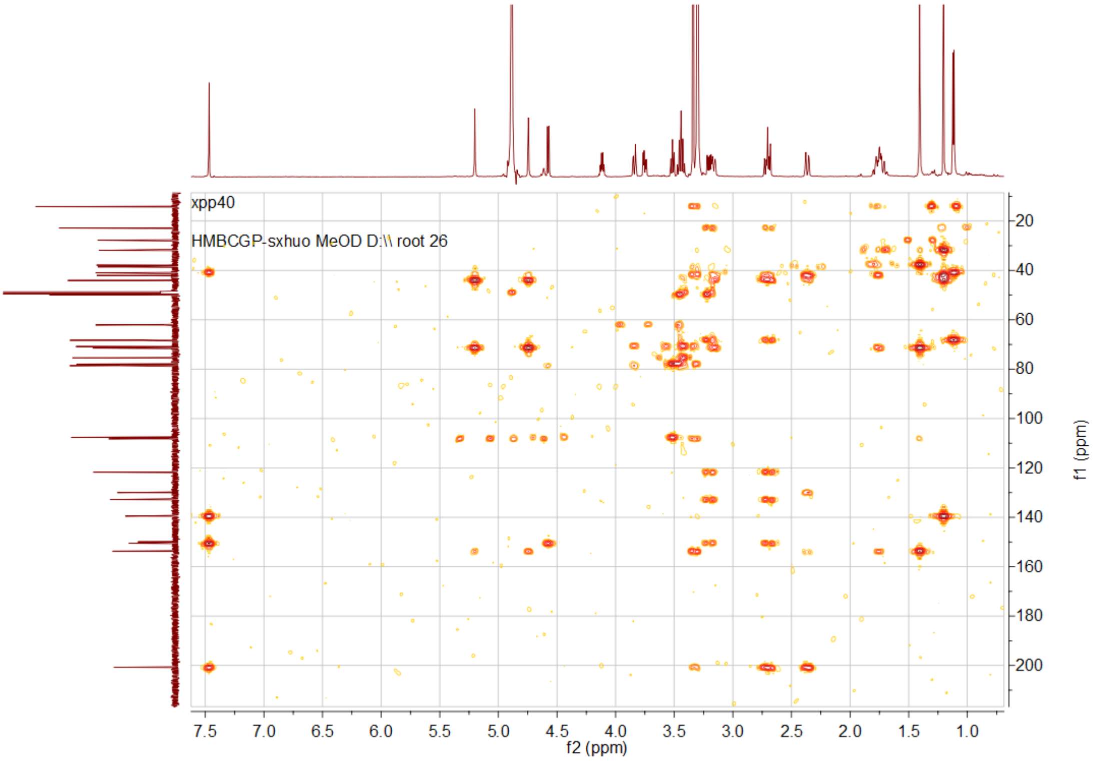

Figure 4S. HMBC spectrum of (**1**) recorded in CD_3OD

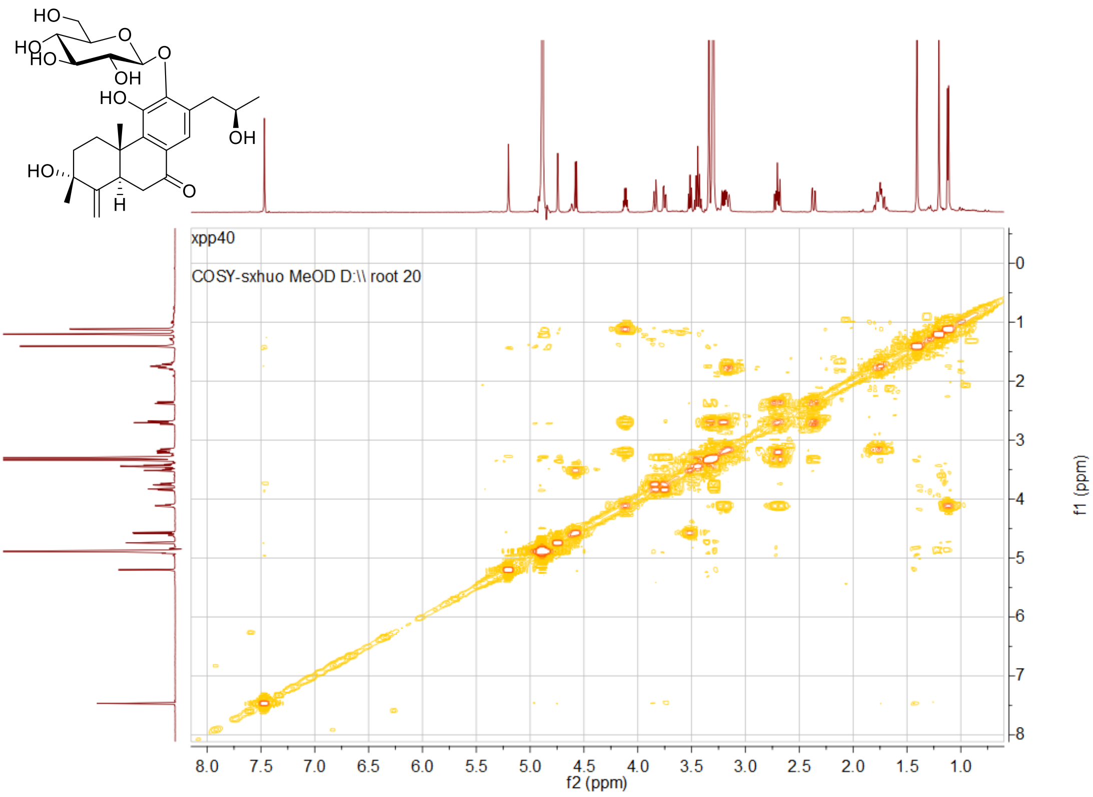

Figure 5S. ^1H-^1H COSY spectrum of (**1**) recorded in CD_3OD

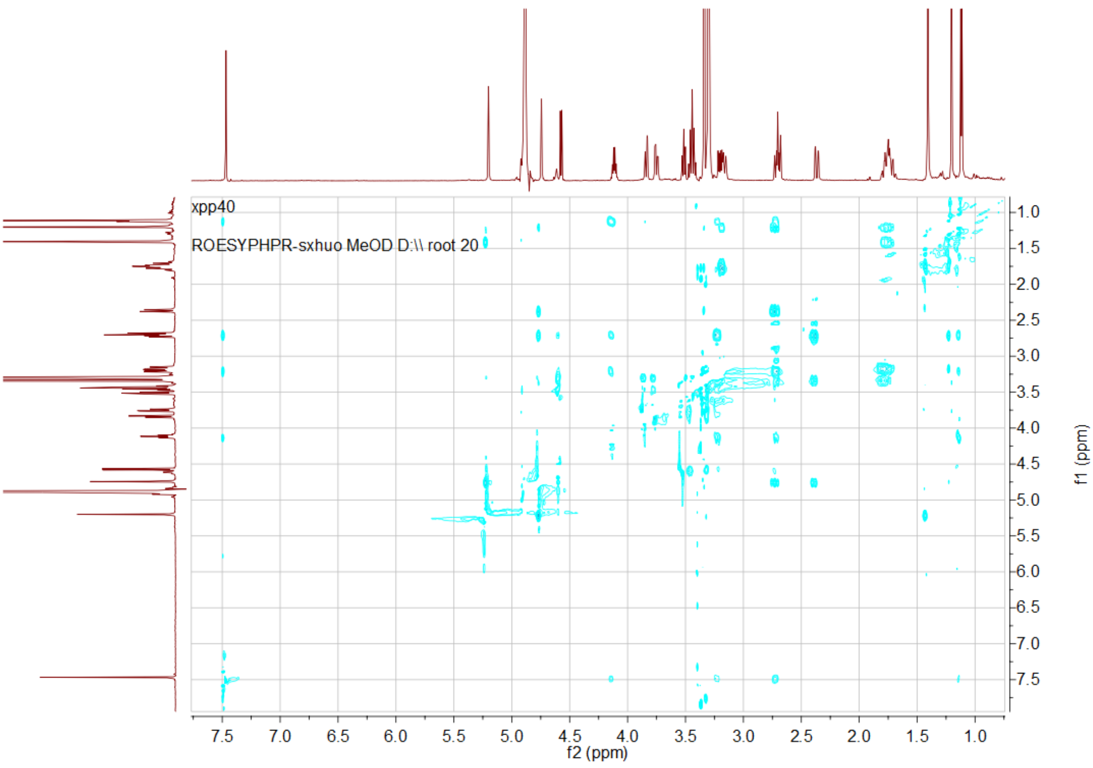

Figure 6S. ROESY spectrum of (**1**) recorded in CD_3OD

Qualitative Analysis Report

**Data Filename**	150619ESIA2.d	**Sample Name**	xpp40
**Sample Type**	Sample	**Position**	
**Instrument Name**	Agilent G6230 TOF MS	**User Name**	KIB
**Acq Method**	ESI.m	**Acquired Time**	6/19/2015 10:57:31 AM
**IRM Calibration Status**	Success	**DA Method**	ESI.m
**Comment**			

**Sample Group**		**Info.**
**Acquisition SW Version**	6200 series TOF/6500 series Q-TOF B.05.01 (B5125.2)	

User Spectra

Fragmentor Voltage	Collision Energy	Ionization Mode
200	0	ESI

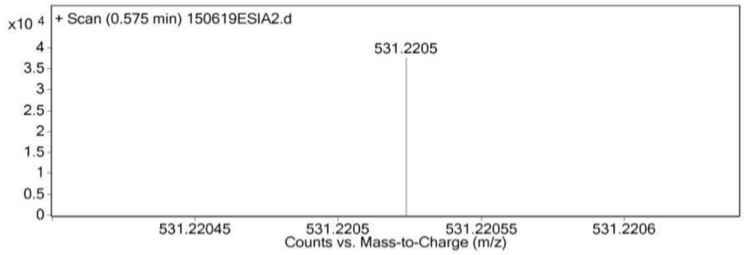

Peak List

m/z	z	Abund
232.1121	1	51983.9
274.2746	1	287136.88
302.3055	1	63239.65
318.3009	1	216545.81
340.2827	1	140159.63
362.3266	1	55996.97
384.3094	1	290803.72
385.3121	1	53854.75
428.3351	1	93969.27
437.1941	1	49979.02

Formula Calculator Element Limits

Element	Min	Max
C	0	200
H	0	400
O	5	14
Na	1	1

Formula Calculator Results

Formula	CalculatedMass	CalculatedMz	Mz	Diff. (mDa)	Diff. (ppm)	DBE
C26 H36 Na O10	531.2206	531.2201	531.2205	-0.5	-0.9	8.5000

--- End Of Report ---

Agilent Technologies Page 1 of 1 Printed at: 11:13 AM on: 6/19/2015

Figure 7S. HRESIMS spectrum of (**1**)

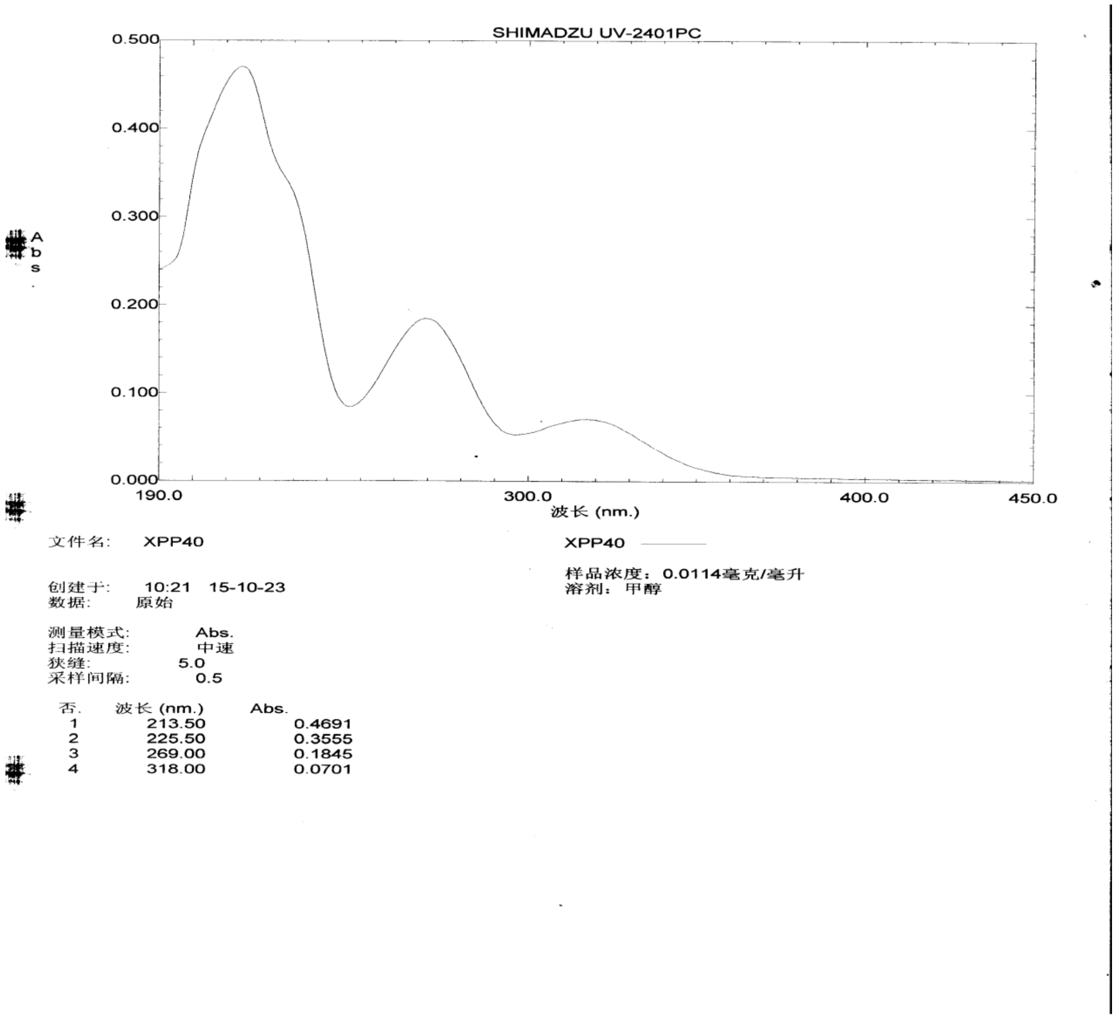

Figure 8S. UV spectrum of (**1**)

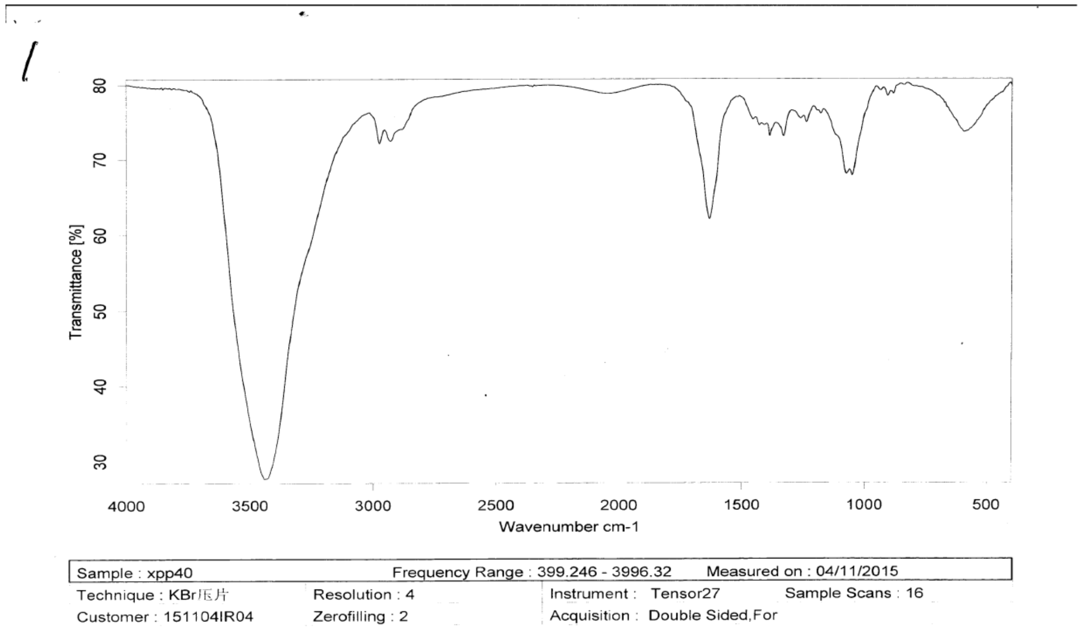

Figure 9S. IR spectrum of (**1**)

Figure 10S-18S. NMR, MS, UV, and IR spectra of compound 2

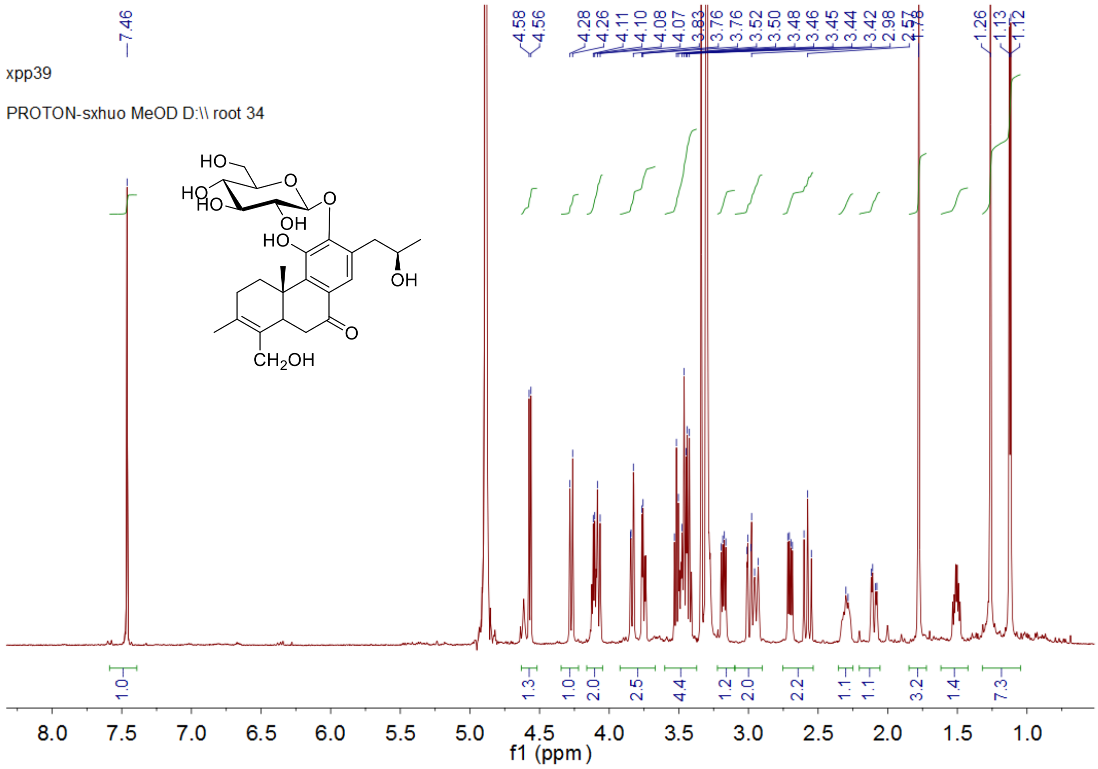

Figure 10S. ^{1}H NMR spectrum of (**2**) recorded in CD_3OD at 600 MHz

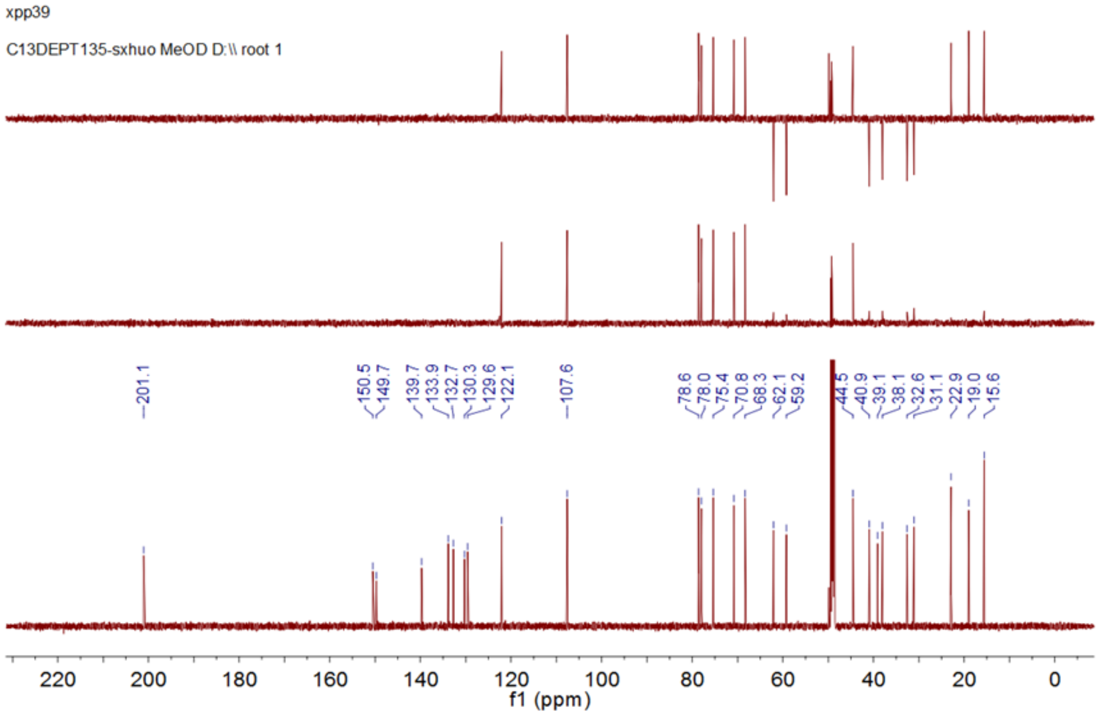

Figure 11S. ^{13}C NMR spectrum of (**2**) recorded in CD_3OD at 150 MHz

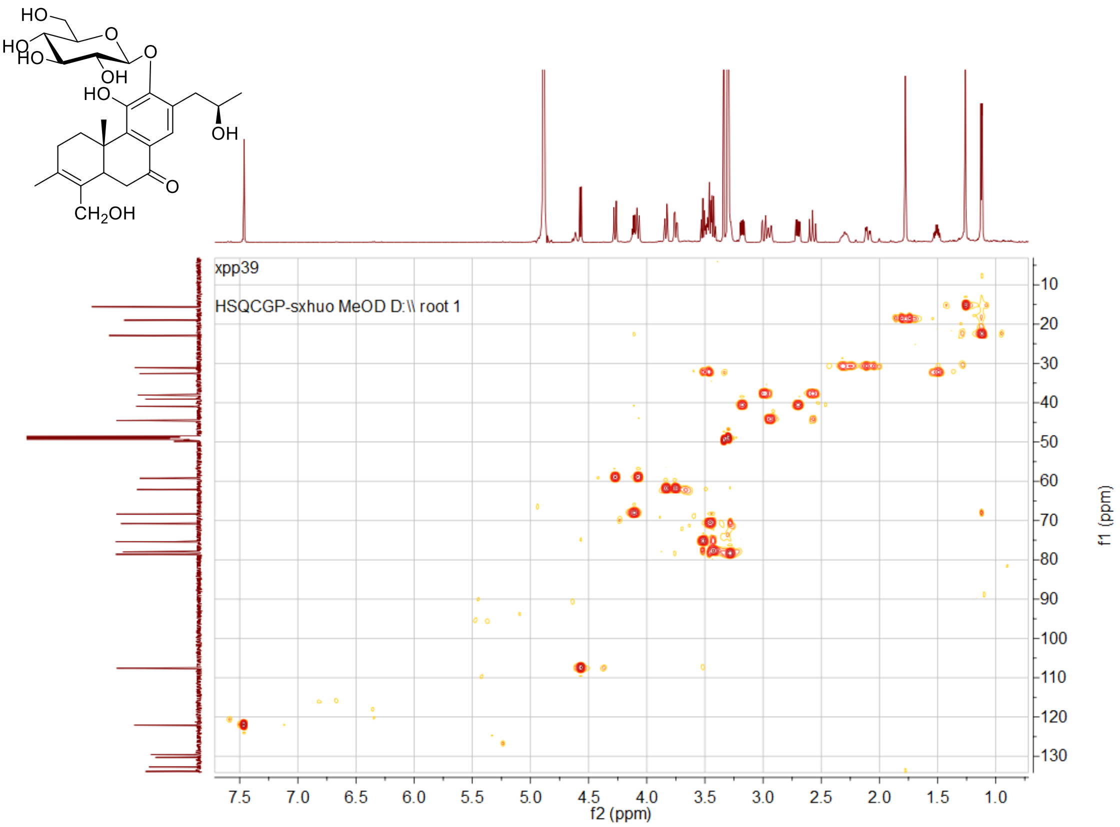

Figure 12S. HSQC spectrum of (**2**) recorded in CD_3OD

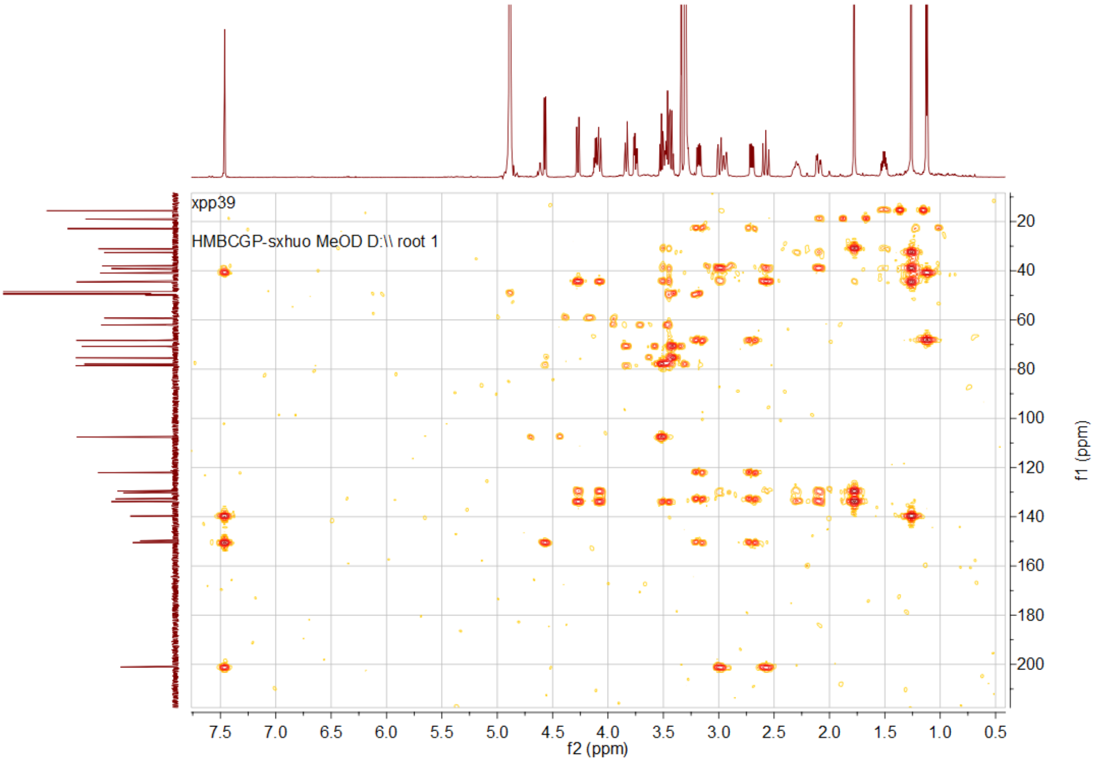

Figure 13S. HMBC spectrum of (**2**) recorded in CD_3OD

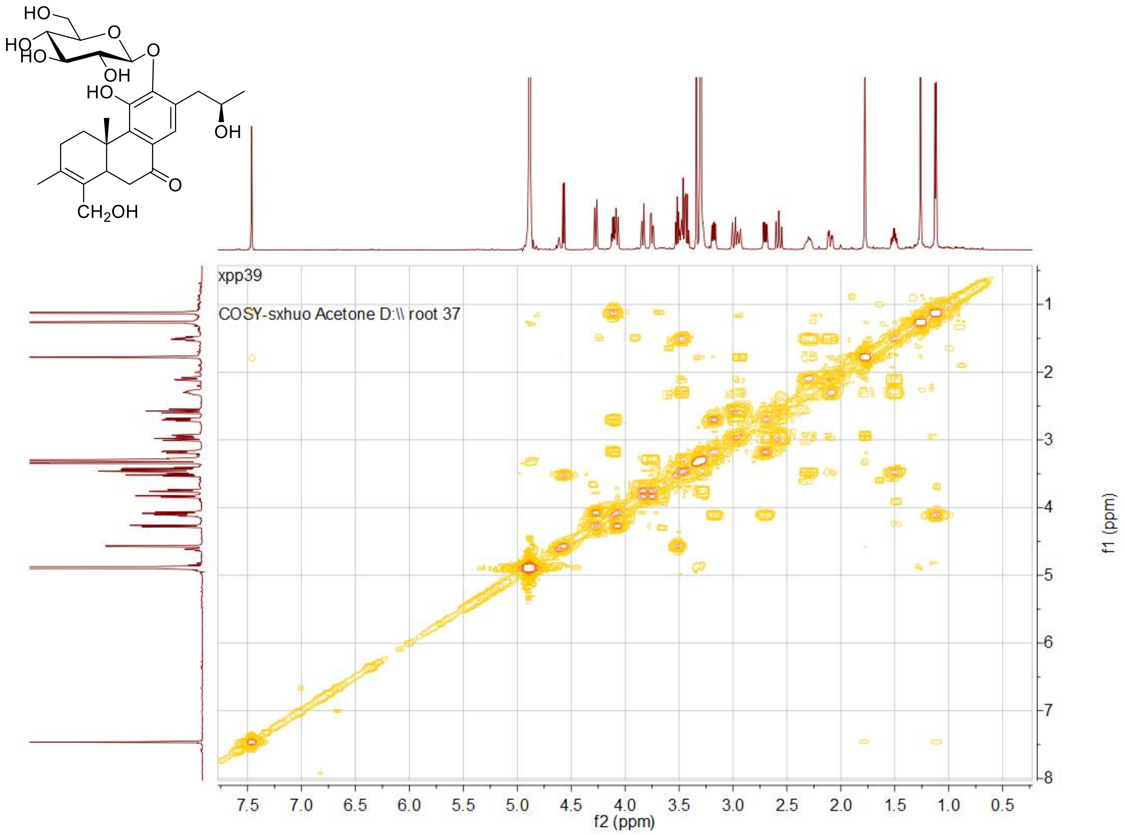

Figure 14S. ^{1}H-^{1}H COSY spectrum of (**2**) recorded in CD_3OD

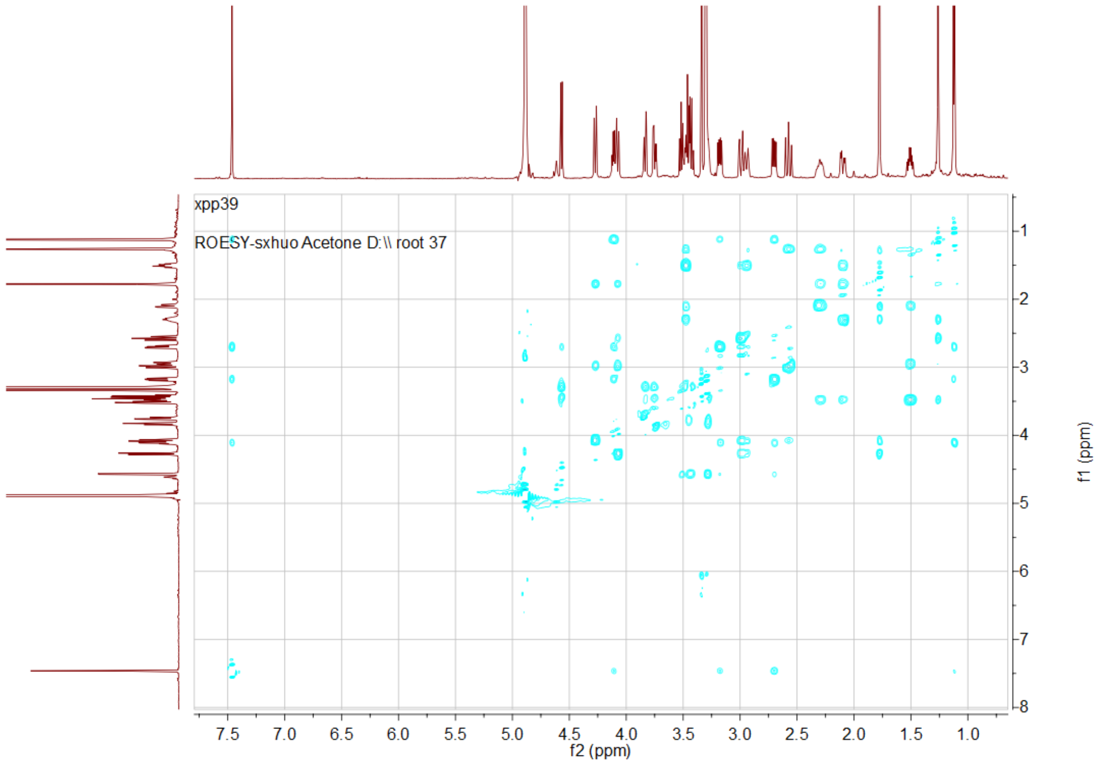

Figure 15S. ROESY spectrum of (**2**) recorded in CD_3OD

Qualitative Analysis Report

**Data Filename**	150619ESIA1.d	**Sample Name**	xpp39
**Sample Type**	Sample	**Position**	
**Instrument Name**	Agilent G6230 TOF MS	**User Name**	KIB
**Acq Method**	ESI.m	**Acquired Time**	6/19/2015 10:55:55 AM
**IRM Calibration Status**	Success	**DA Method**	ESI.m
**Comment**			

**Sample Group**		**Info.**
**Acquisition SW Version**	6200 series TOF/6500 series Q-TOF B.05.01 (B5125.2)	

User Spectra

Fragmentor Voltage	Collision Energy	Ionization Mode
200	0	ESI

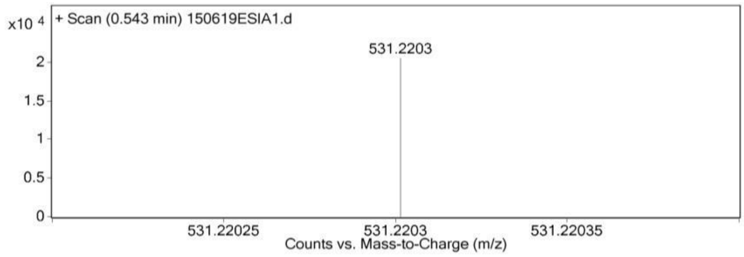

Peak List

m/z	z	Abund
121.0509	1	50208.17
232.1123	1	272387.31
274.2743	1	253471.84
302.3051	1	48710.32
318.3006	1	182545.94
340.2825	1	169587.23
362.3266	1	45929.96
384.3089	1	234708.81
428.3348	1	89506.63
922.0098	1	56326.68

Formula Calculator Element Limits

Element	Min	Max
C	0	200
H	0	400
O	5	14
Na	1	1

Formula Calculator Results

Formula	CalculatedMass	CalculatedMz	Mz	Diff. (mDa)	Diff. (ppm)	DBE
C26 H36 Na O10	531.2206	531.2201	531.2203	-0.3	-0.5	8.5000

--- End Of Report ---

Figure 16S. HRESIMS spectrum of (**2**)

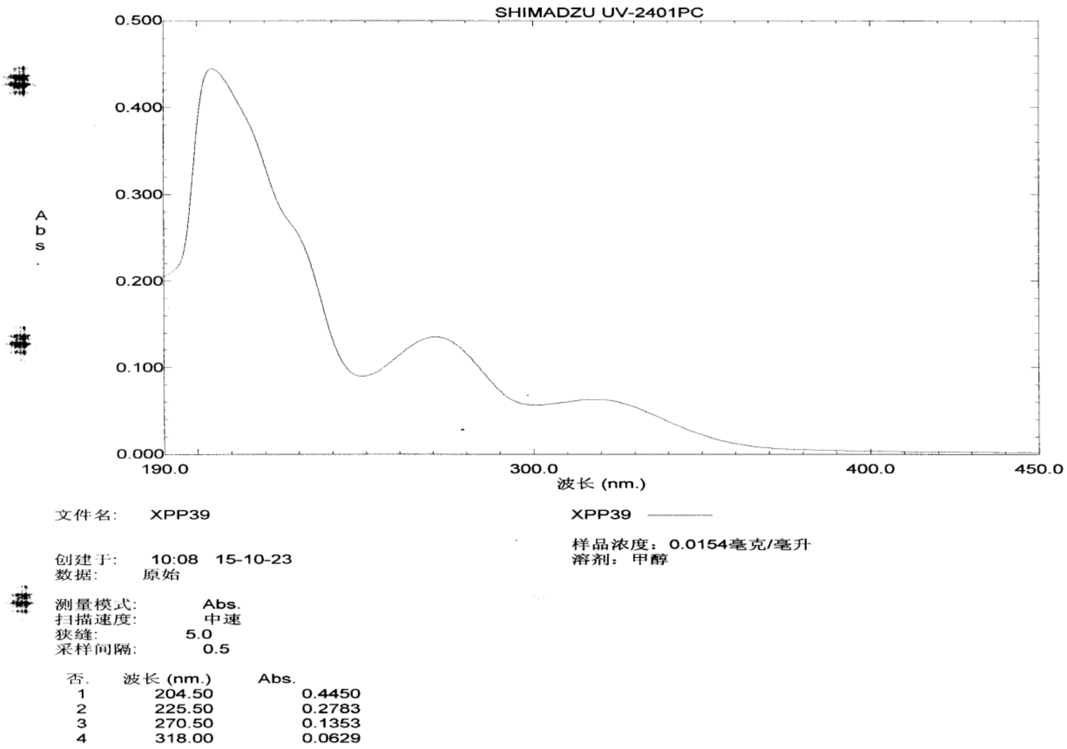

Figure 17S. UV spectrum of (**2**)

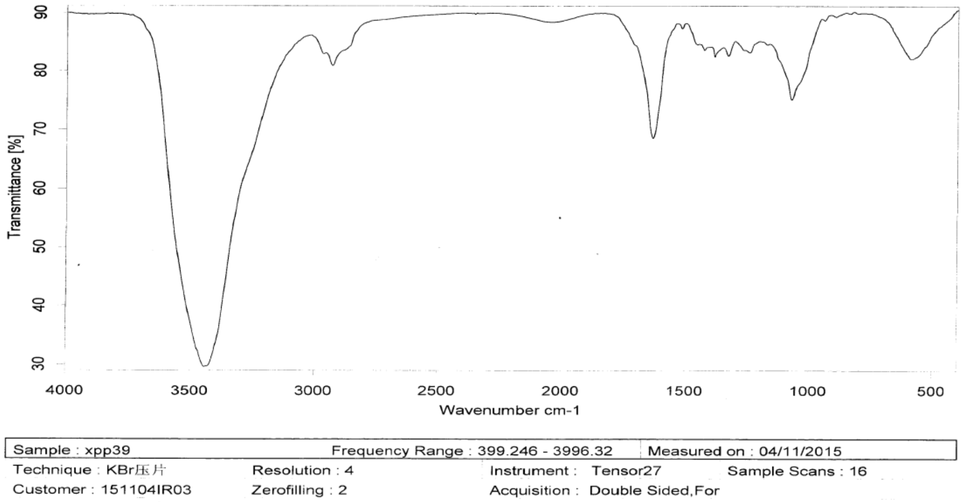

Figure 18S. IR spectrum of (**2**)

Figure 19S-27S. NMR, MS, UV, and IR spectra of compound 3

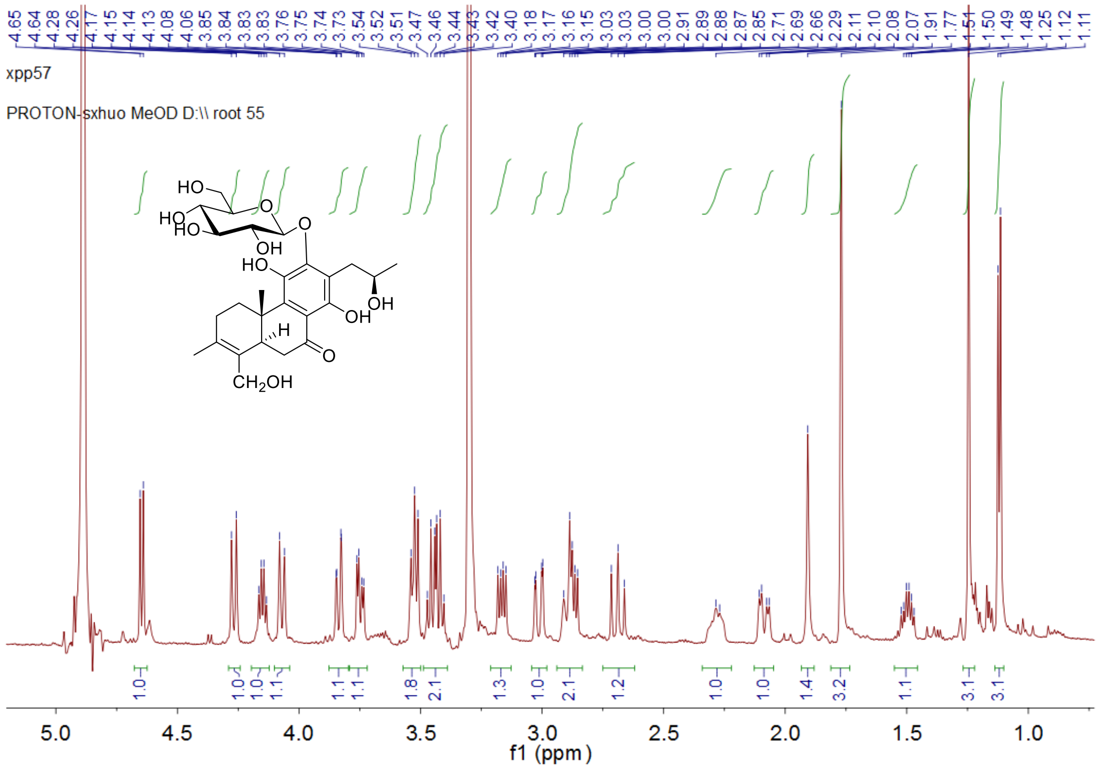

Figure 19S. ^{1}H NMR spectrum of (**3**) recorded in CD_3OD at 600 MHz

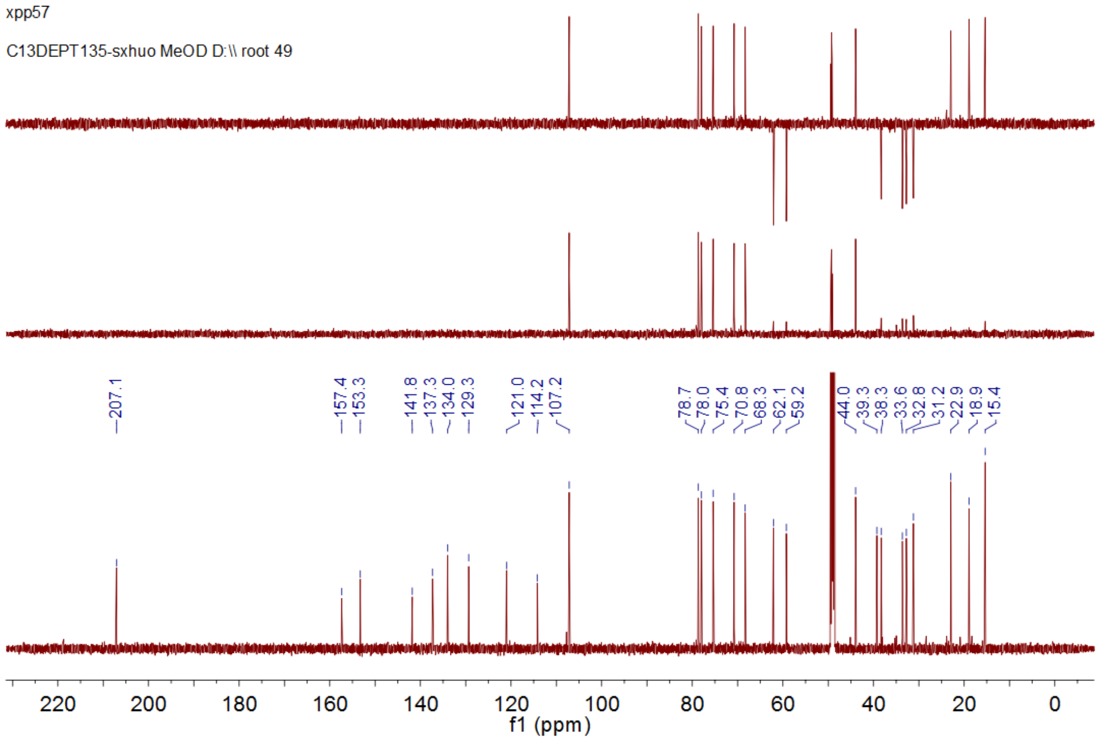

Figure 20S. ^{13}C NMR spectrum of (**3**) recorded in CD_3OD at 150 MHz

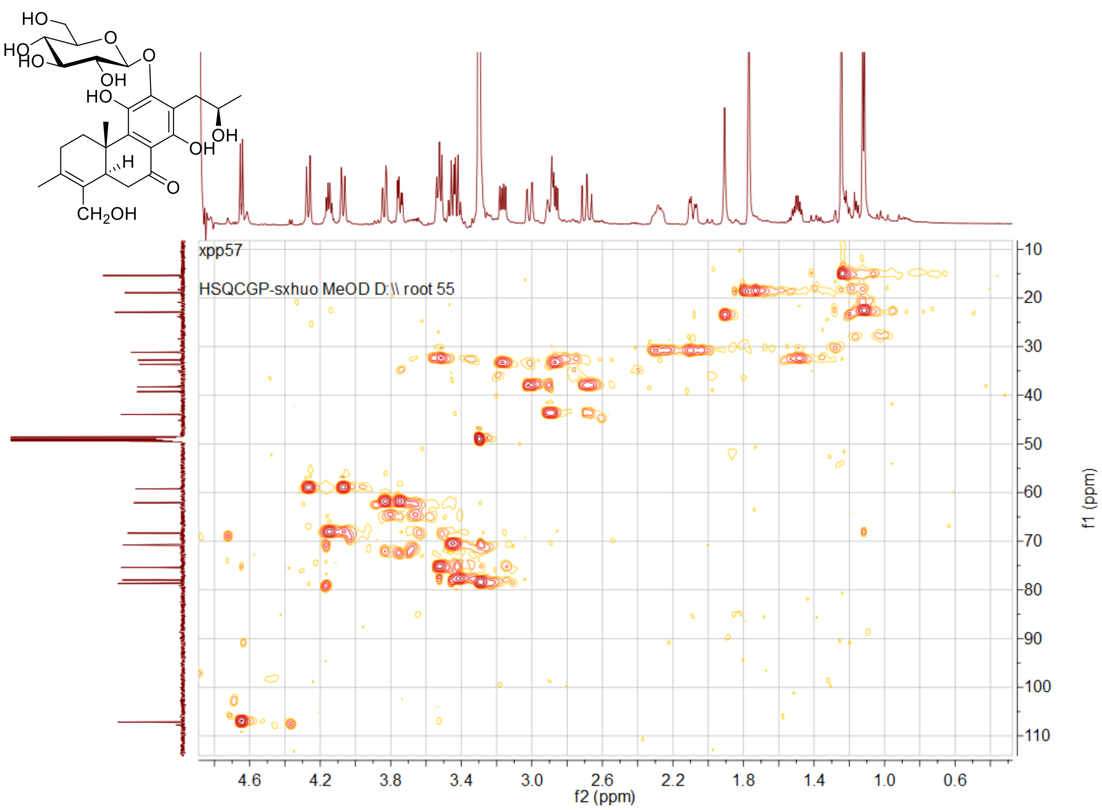

Figure 21S. HSQC spectrum of (**3**) recorded in CD_3OD

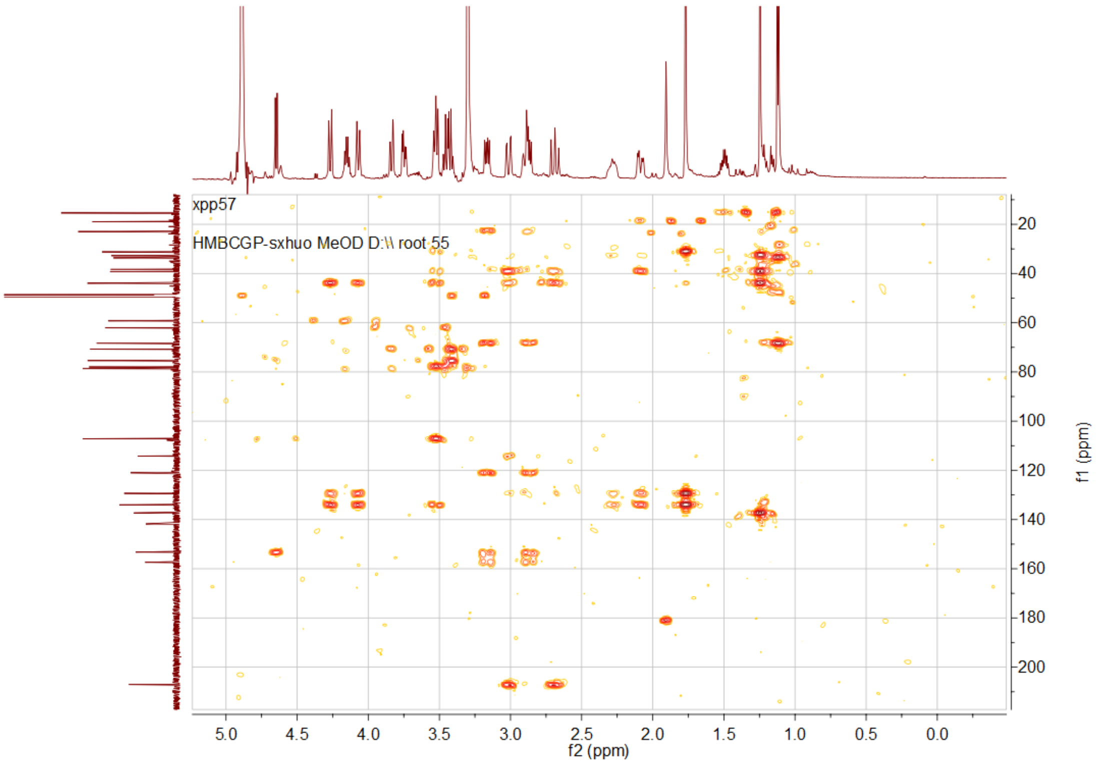

Figure 22S. HMBC spectrum of (**3**) recorded in CD_3OD

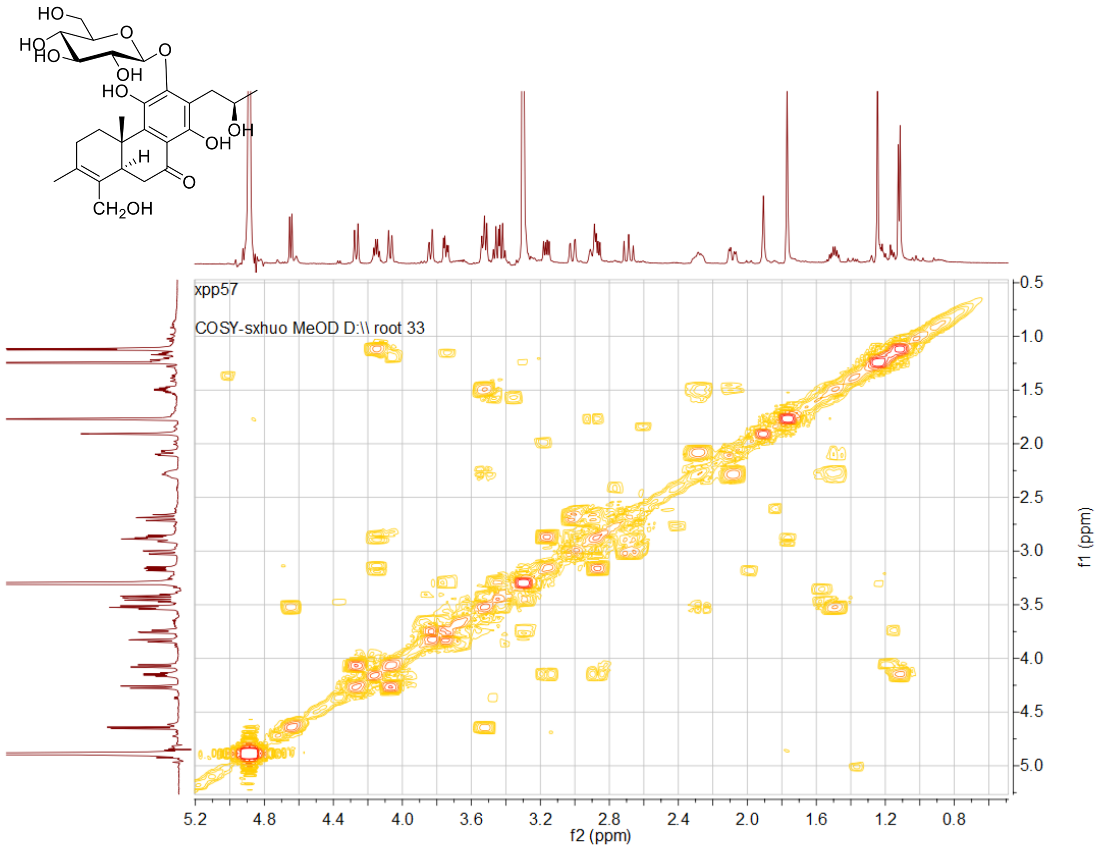

Figure 23S. ^{1}H-^{1}H COSY spectrum of (**3**) recorded in CD_3OD

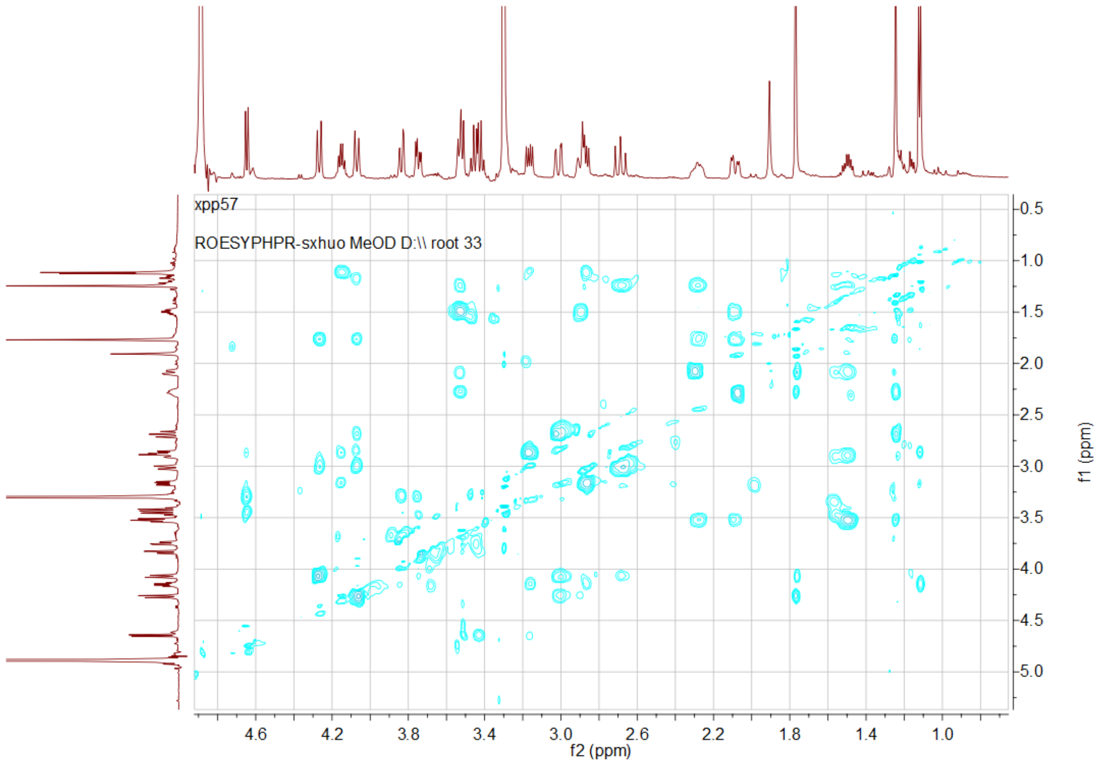

Figure 24S. ROESY spectrum of (**3**) recorded in CD_3OD

Qualitative Analysis Report

**Data Filename**	150630ESINA3.d	**Sample Name**	xpp57
**Sample Type**	Sample	**Position**	
**Instrument Name**	Agilent G6230 TOF MS	**User Name**	KIB
**Acq Method**	ESIN.m	**Acquired Time**	6/30/2015 2:53:52 PM
**IRM Calibration Status**	Success	**DA Method**	ESI.m
**Comment**			

**Sample Group**		**Info.**
**Acquisition SW Version**	6200 series TOF/6500 series Q-TOF B.05.01 (B5125.2)	

User Spectra

Fragmentor Voltage	Collision Energy	Ionization Mode
200	0	ESI

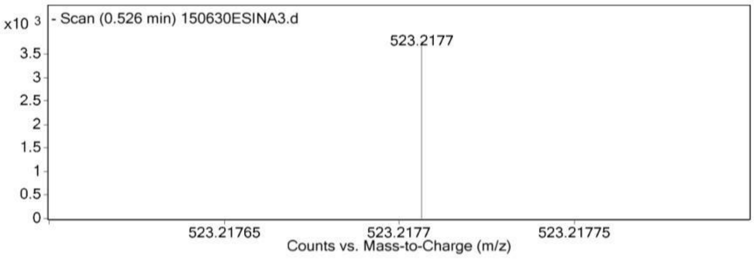

Peak List

m/z	z	Abund	Formula	Ion
112.9856		2859.45		
154.9735		872.07		
255.233		735.66		
523.2177	1	3768.33	C26 H35 O11	M-
524.2217	1	648.16	C26 H35 O11	M-
1033.9881	1	182550.59		
1034.9889	1	22363.53		
1035.9911	1	1034.86		
1933.9283	1	26144.18		
1934.9299	1	4145.85		

Formula Calculator Element Limits

Element	Min	Max
C	0	200
H	0	400
O	6	14

Formula Calculator Results

Formula	CalculatedMass	CalculatedMz	Mz	Diff. (mDa)	Diff. (ppm)	DBE
C26 H35 O11	523.2179	523.2185	523.2177	0.7	1.3	9.5000

--- End Of Report ---

Agilent Technologies   Page 1 of 1   Printed at: 3:03 PM on: 6/30/2015

Figure 25S. HRESIMS spectrum of (**3**)

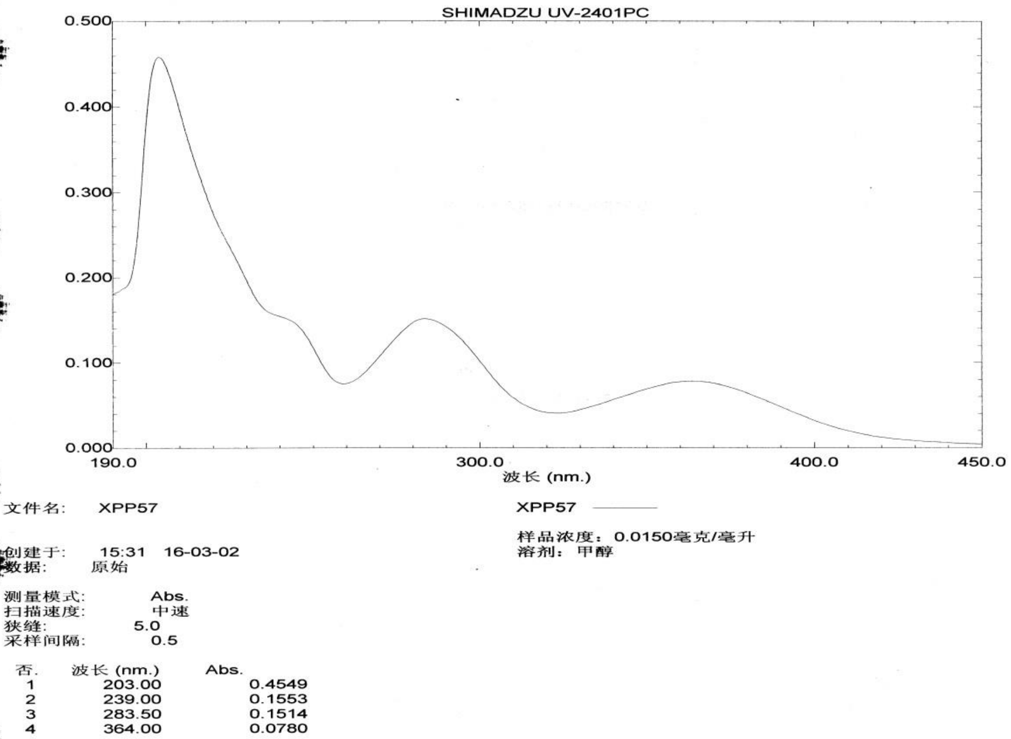

Figure 26S. UV spectrum of (**3**)

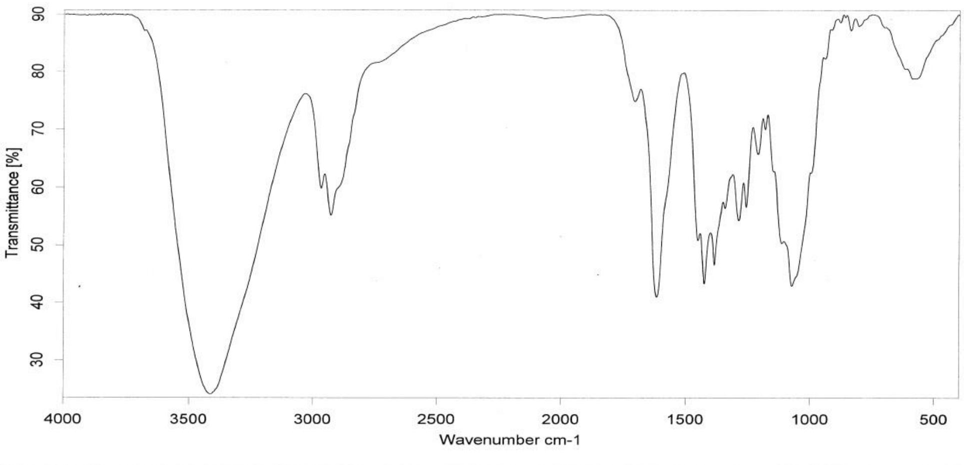

Sample : xpp57	Frequency Range : 399.246 - 3996.32	Measured on : 04/03/2016	
Technique : KBr压片	Resolution : 4	Instrument : Tensor27	Sample Scans : 16
Customer : 160304IR9	Zerofilling : 2	Acquisition : Double Sided,For	

Figure 27S. IR spectrum of (**3**)

Figure 28S-36S. NMR, MS, UV, and IR spectra of compound 4

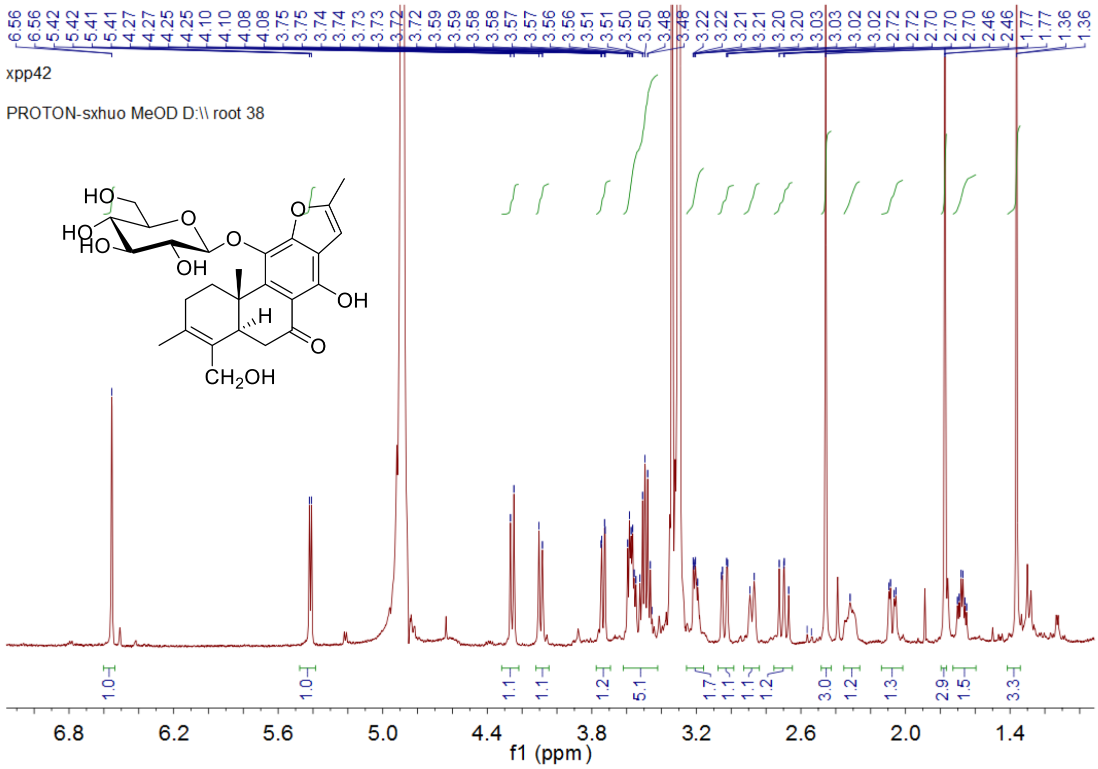

Figure 28S. ^{1}H NMR spectrum of (**4**) recorded in CD_3OD at 600 MHz

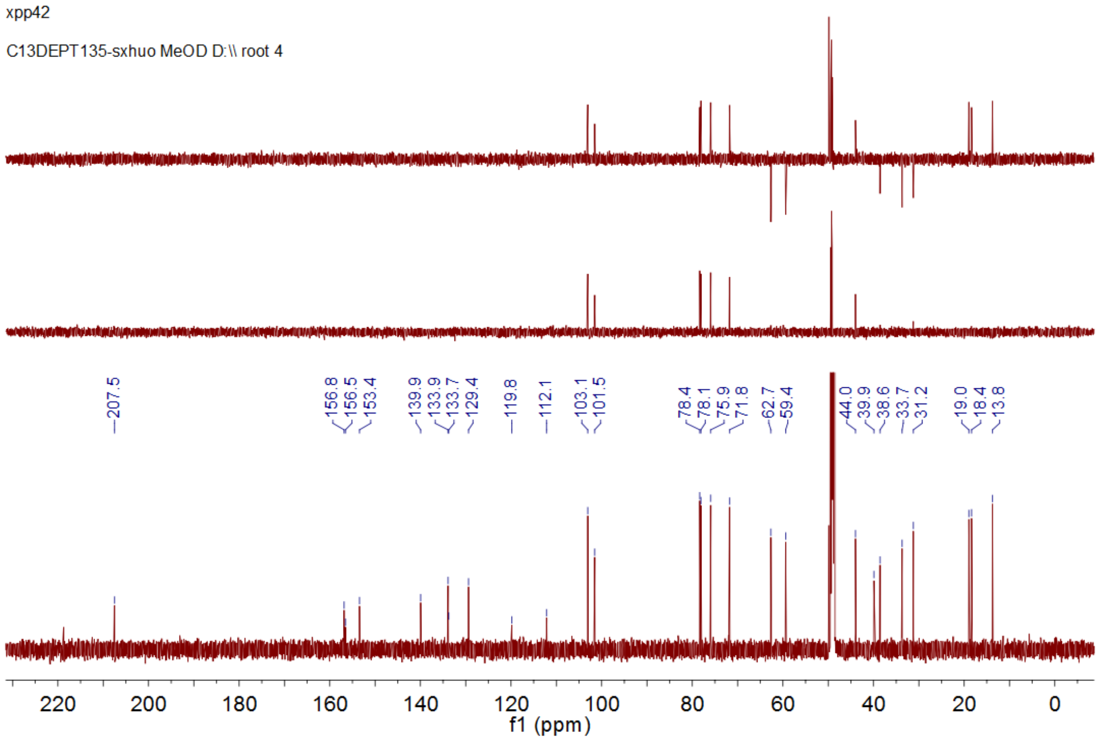

Figure 29S. ^{13}C NMR spectrum of (**4**) recorded in CD_3OD at 150 MHz

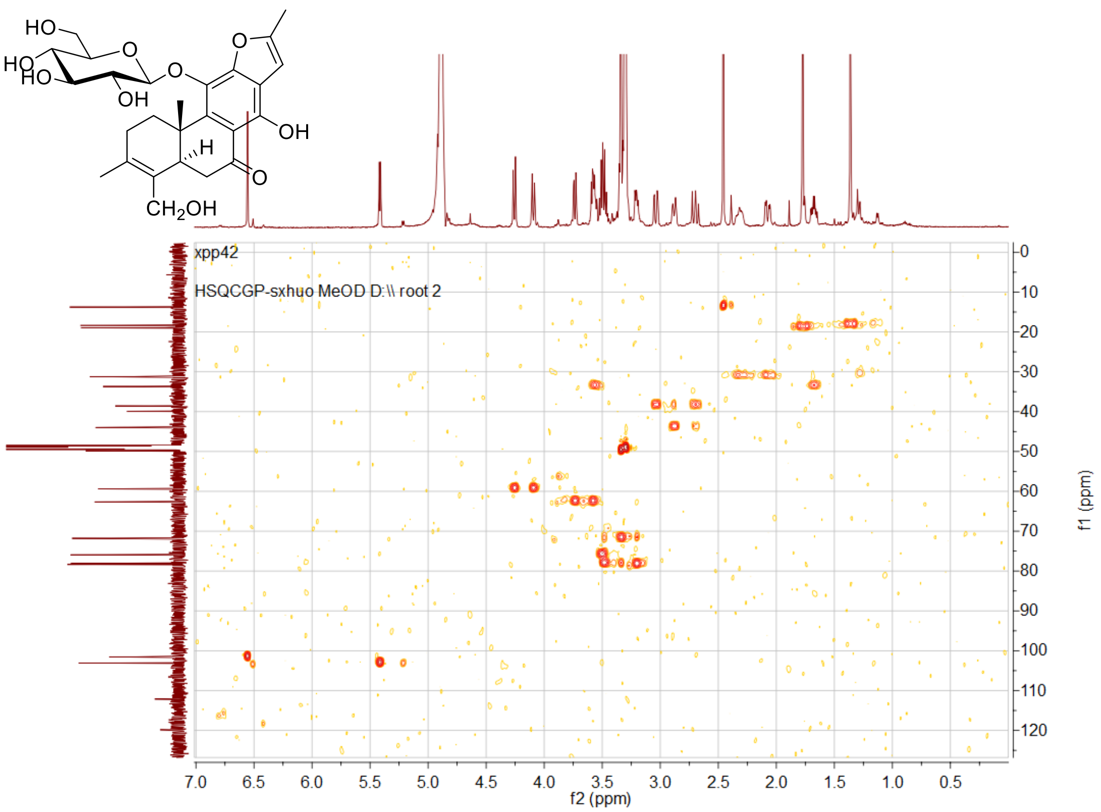

Figure 30S. HSQC spectrum of (**4**) recorded in CD_3OD

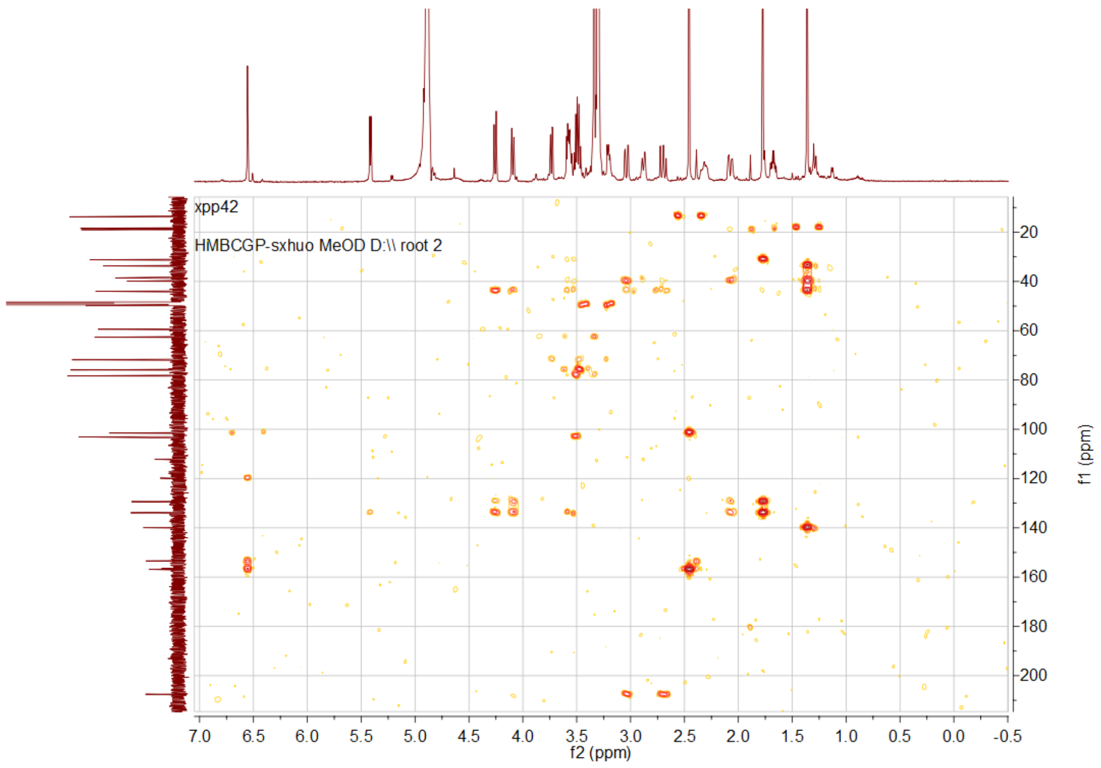

Figure 31S. HMBC spectrum of (**4**) recorded in CD_3OD

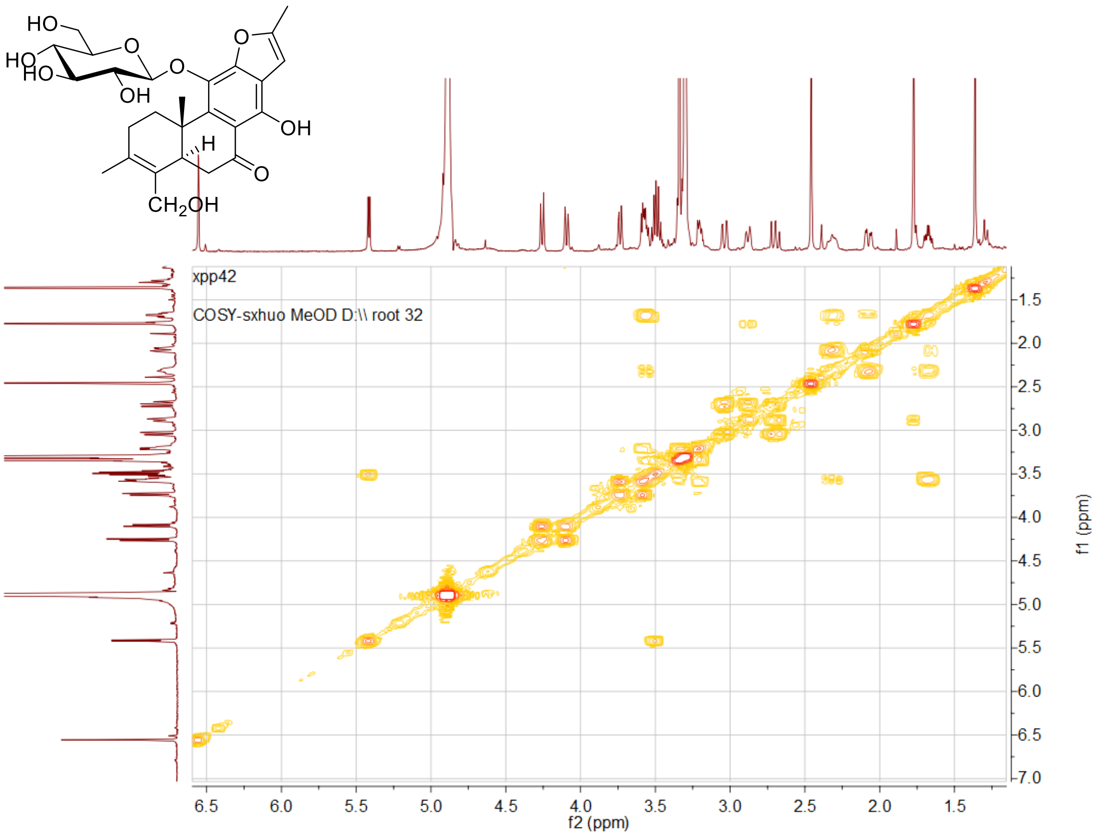

Figure 32S. ^{1}H-^{1}H COSY spectrum of (**4**) recorded in CD_3OD

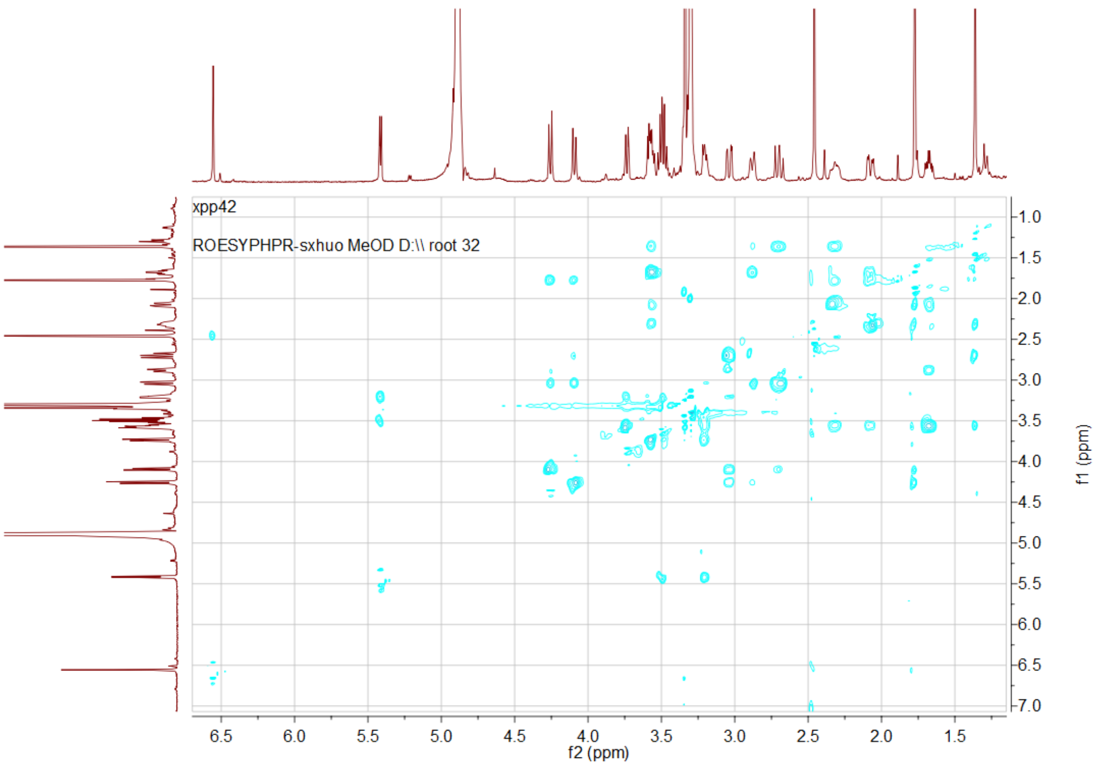

Figure 33S. ROESY spectrum of (**4**) recorded in CD_3OD

Qualitative Analysis Report

**Data Filename**	150619ESIA4.d	**Sample Name**	xpp42
**Sample Type**	Sample	**Position**	
**Instrument Name**	Agilent G6230 TOF MS	**User Name**	KIB
**Acq Method**	ESI.m	**Acquired Time**	6/19/2015 11:01:27 AM
**IRM Calibration Status**	Success	**DA Method**	ESI.m
**Comment**			

**Sample Group**		**Info.**
**Acquisition SW Version**	6200 series TOF/6500 series Q-TOF B.05.01 (B5125.2)	

User Spectra

Fragmentor Voltage	Collision Energy	Ionization Mode
200	0	ESI

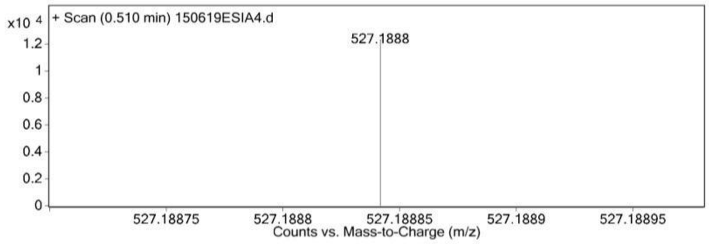

Peak List

m/z	z	Abund
274.2747	1	507096.06
302.3056	1	96918.77
318.301	1	443766.19
319.3039	1	78546.8
340.283	1	274464.25
362.327	1	133373.97
384.3095	1	557409.81
385.3123	1	108445.31
428.3355	1	247064.61
437.1944	1	102033.87

Formula Calculator Element Limits

Element	Min	Max
C	0	200
H	0	400
O	5	14
Na	1	1

Formula Calculator Results

Formula	CalculatedMass	CalculatedMz	Mz	Diff. (mDa)	Diff. (ppm)	DBE
C26 H32 Na O10	527.1893	527.1888	527.1888	-0.6	-1.1	10.5000

--- End Of Report ---

Agilent Technologies   Page 1 of 1   Printed at: 11:08 AM on: 6/19/2015

Figure 34S. HRESIMS spectrum of (**4**)

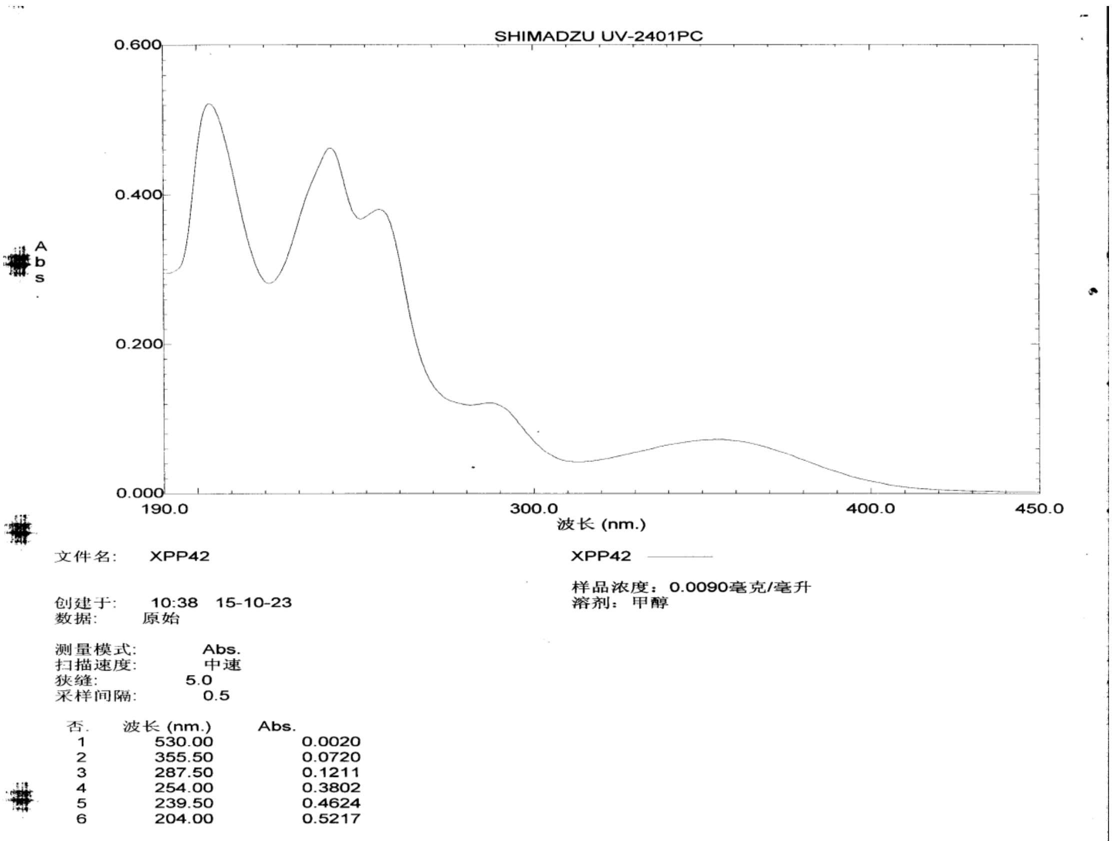

Figure 35S. UV spectrum of (**4**)

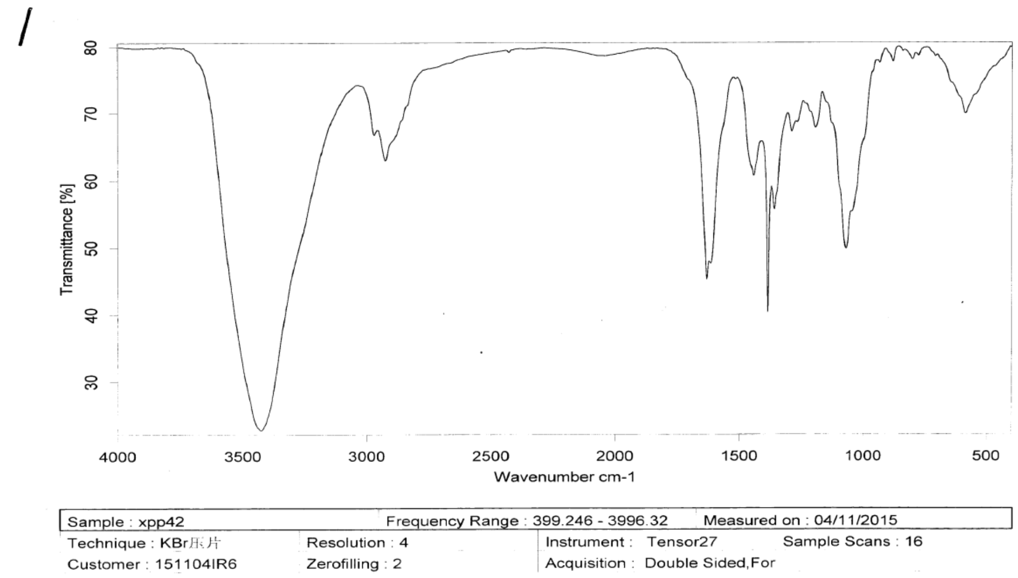

Figure 36S. IR spectrum of (**4**)

Figure 37S-45S. NMR, MS, UV, and IR spectra of compound 5

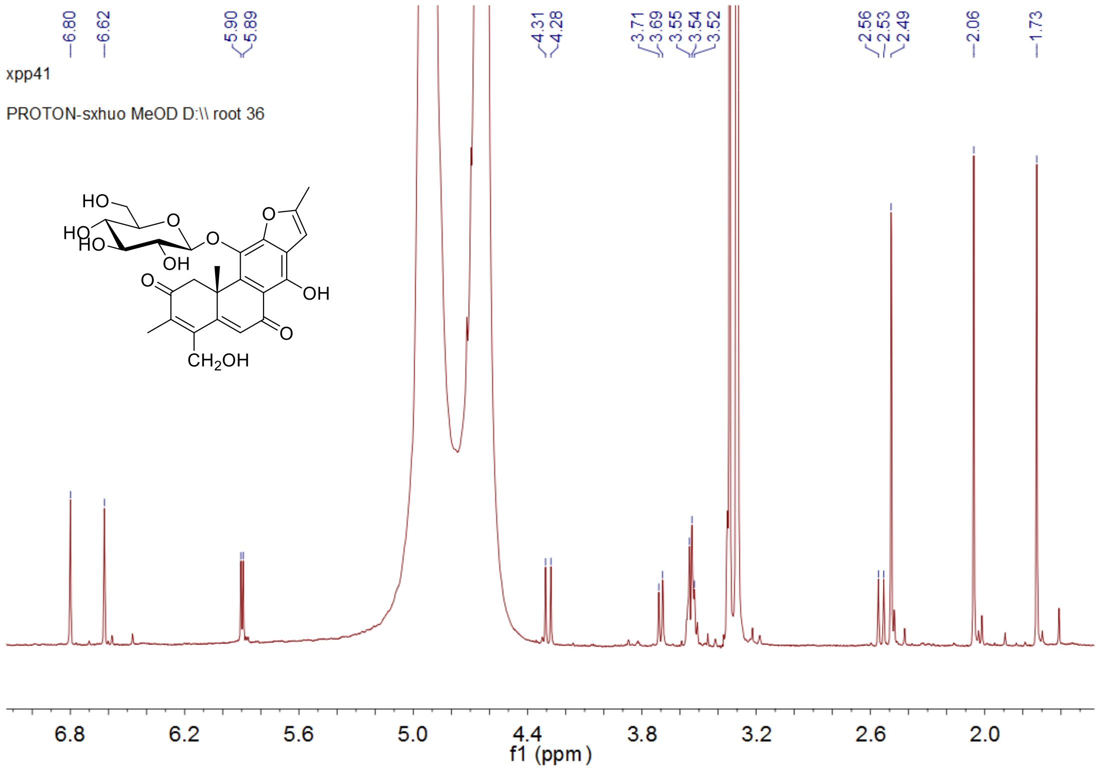

Figure 37S. ^{1}H NMR spectrum of (**5**) recorded in CD_3OD at 600 MHz

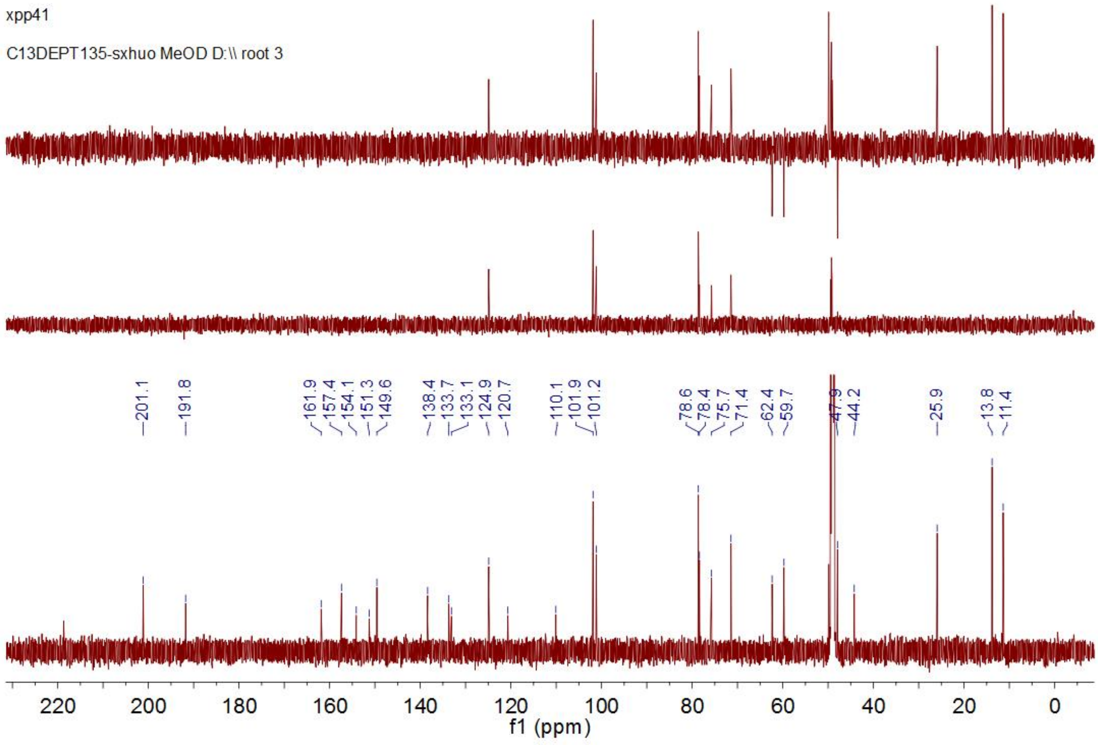

Figure 38S. ^{13}C NMR spectrum of (**5**) recorded in CD_3OD at 150 MHz

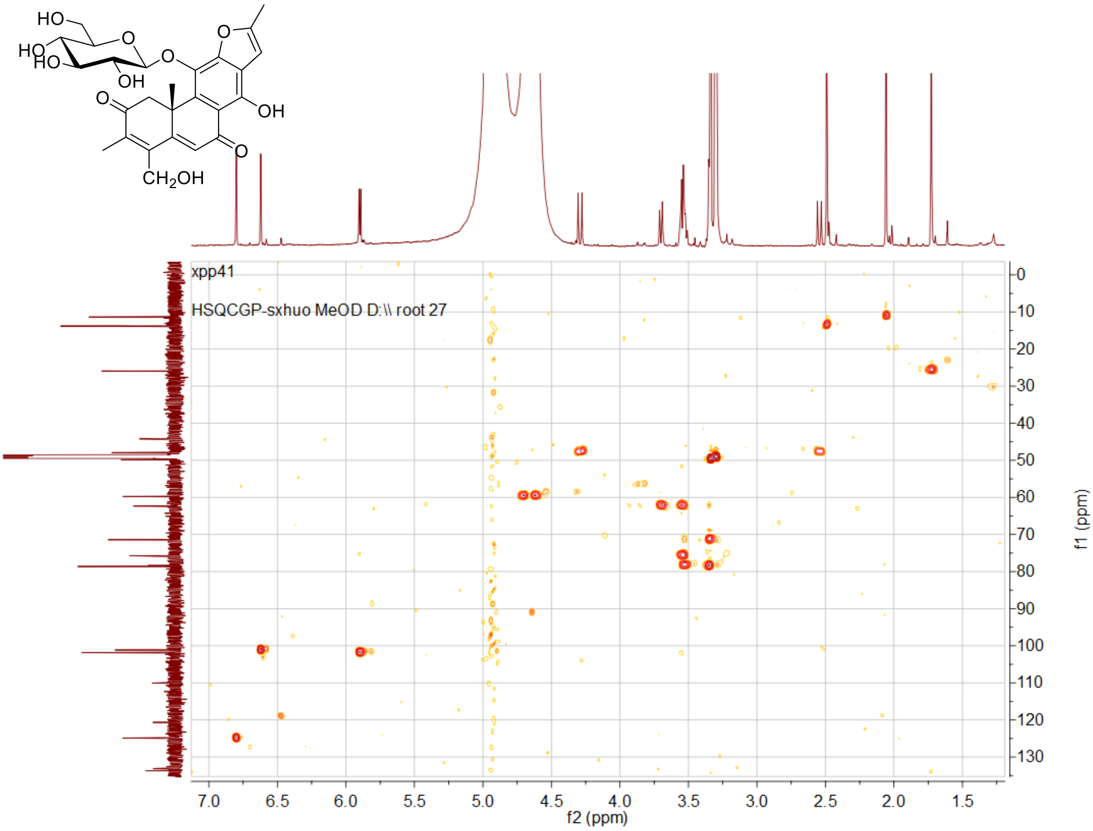

Figure 39S. HSQC spectrum of (**5**) recorded in CD_3OD

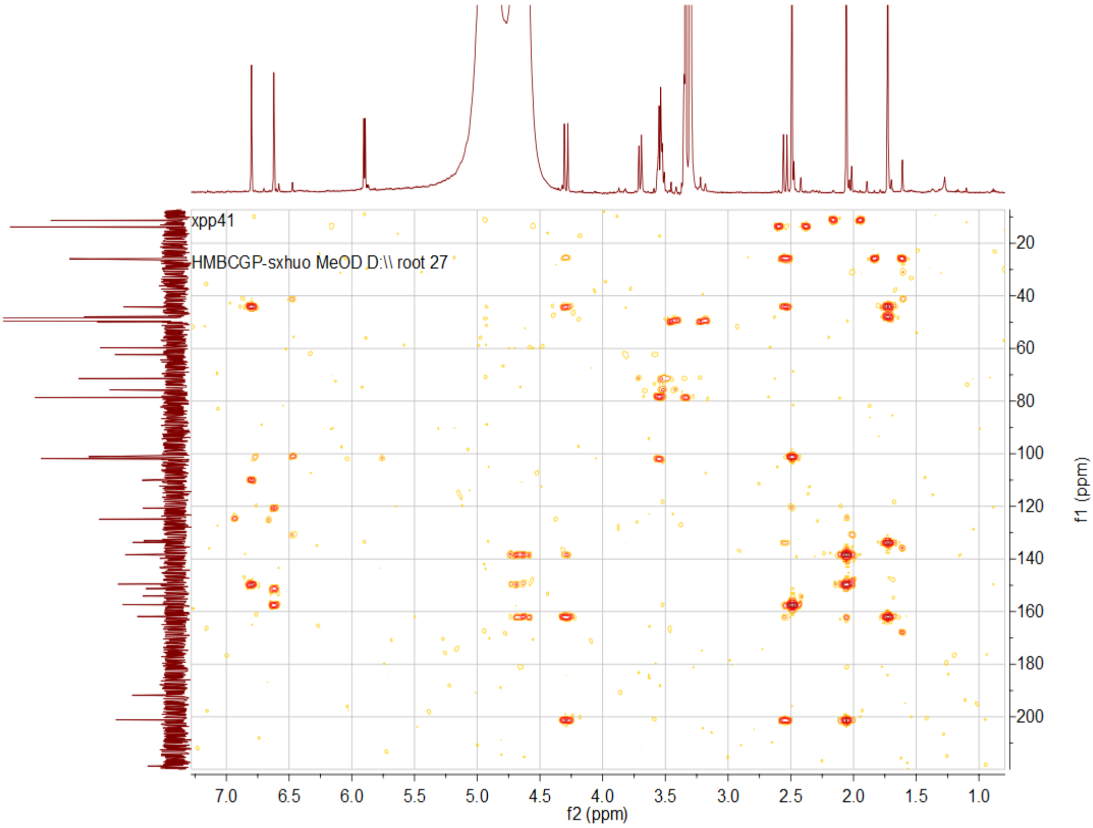

Figure 40S. HMBC spectrum of (**5**) recorded in CD_3OD

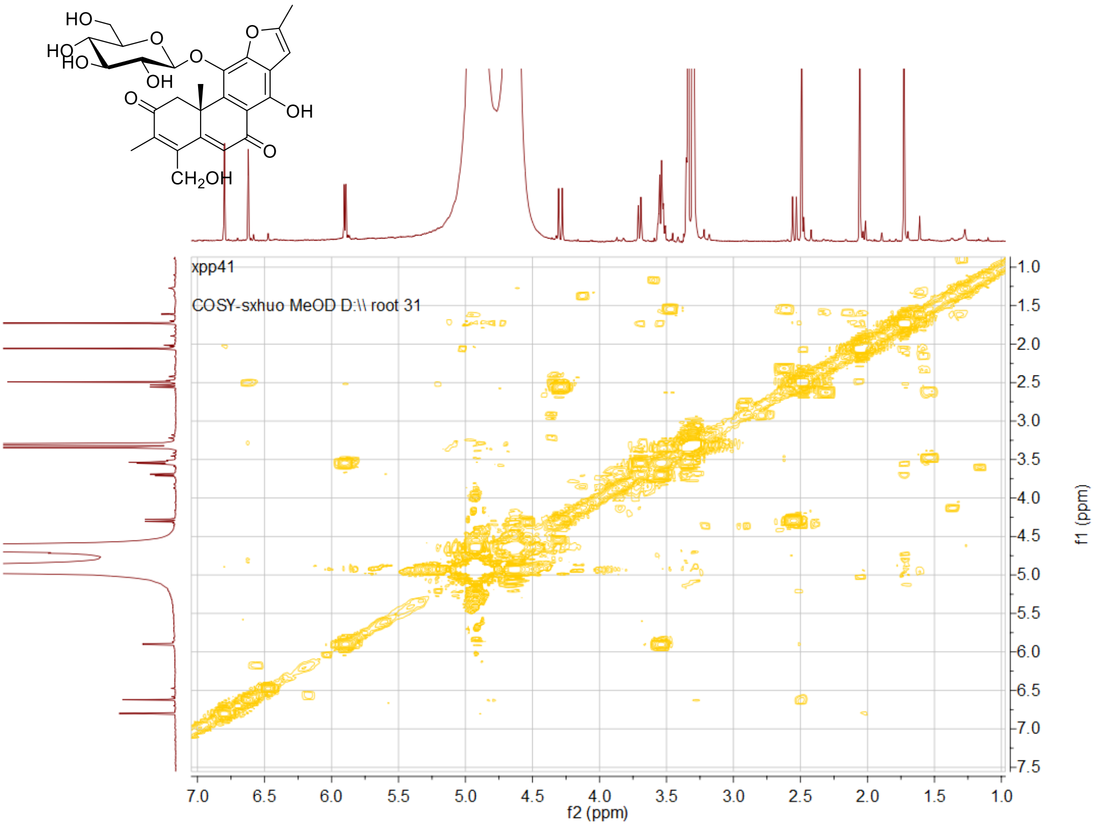

Figure 41S. ^{1}H-^{1}H COSY spectrum of (**5**) recorded in CD_3OD

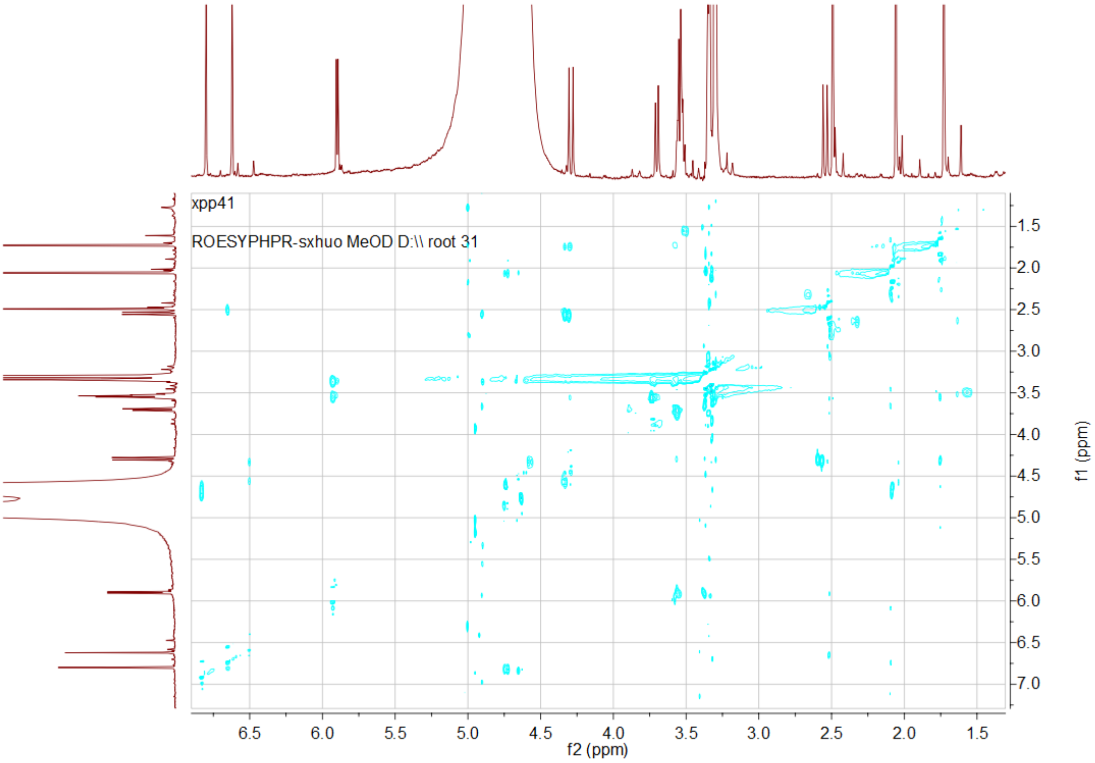

Figure 42S. ROESY spectrum of (**5**) recorded in CD_3OD

Qualitative Analysis Report

**Data Filename**	150619ESIA3.d	**Sample Name**	xpp41
**Sample Type**	Sample	**Position**	
**Instrument Name**	Agilent G6230 TOF MS	**User Name**	KIB
**Acq Method**	ESI.m	**Acquired Time**	6/19/2015 10:59:10 AM
**IRM Calibration Status**	Success	**DA Method**	ESI.m
**Comment**			

Sample Group **Info.**
Acquisition SW Version 6200 series TOF/6500 series Q-TOF B.05.01 (B5125.2)

User Spectra

Fragmentor Voltage	Collision Energy	Ionization Mode
200	0	ESI

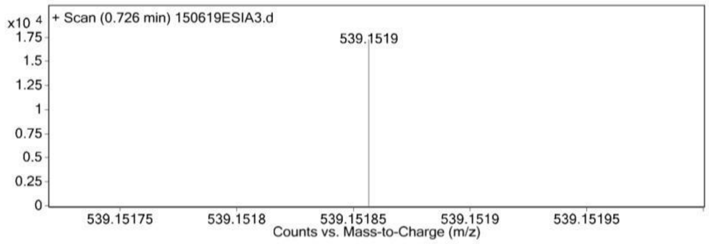

Peak List

m/z	z	Abund
274.2746	1	350092.31
302.3054	1	66818.2
318.3007	1	254742.45
340.2828	1	179022.48
362.3268	1	73820.24
384.3093	1	362414.47
385.3118	1	67499.24
428.3354	1	215189.44
437.1942	1	102722.02
472.3608	1	62139.93

Formula Calculator Element Limits

Element	Min	Max
C	0	200
H	0	400
O	5	14
Na	1	1

Formula Calculator Results

Formula	CalculatedMass	CalculatedMz	Mz	Diff. (mDa)	Diff. (ppm)	DBE
C26 H28 Na O11	539.1529	539.1524	539.1519	0.5	1.0	12.5000

--- End Of Report ---

Agilent Technologies Page 1 of 1 Printed at: 11:11 AM on: 6/19/2015

Figure 43S. HRESIMS spectrum of (**5**)

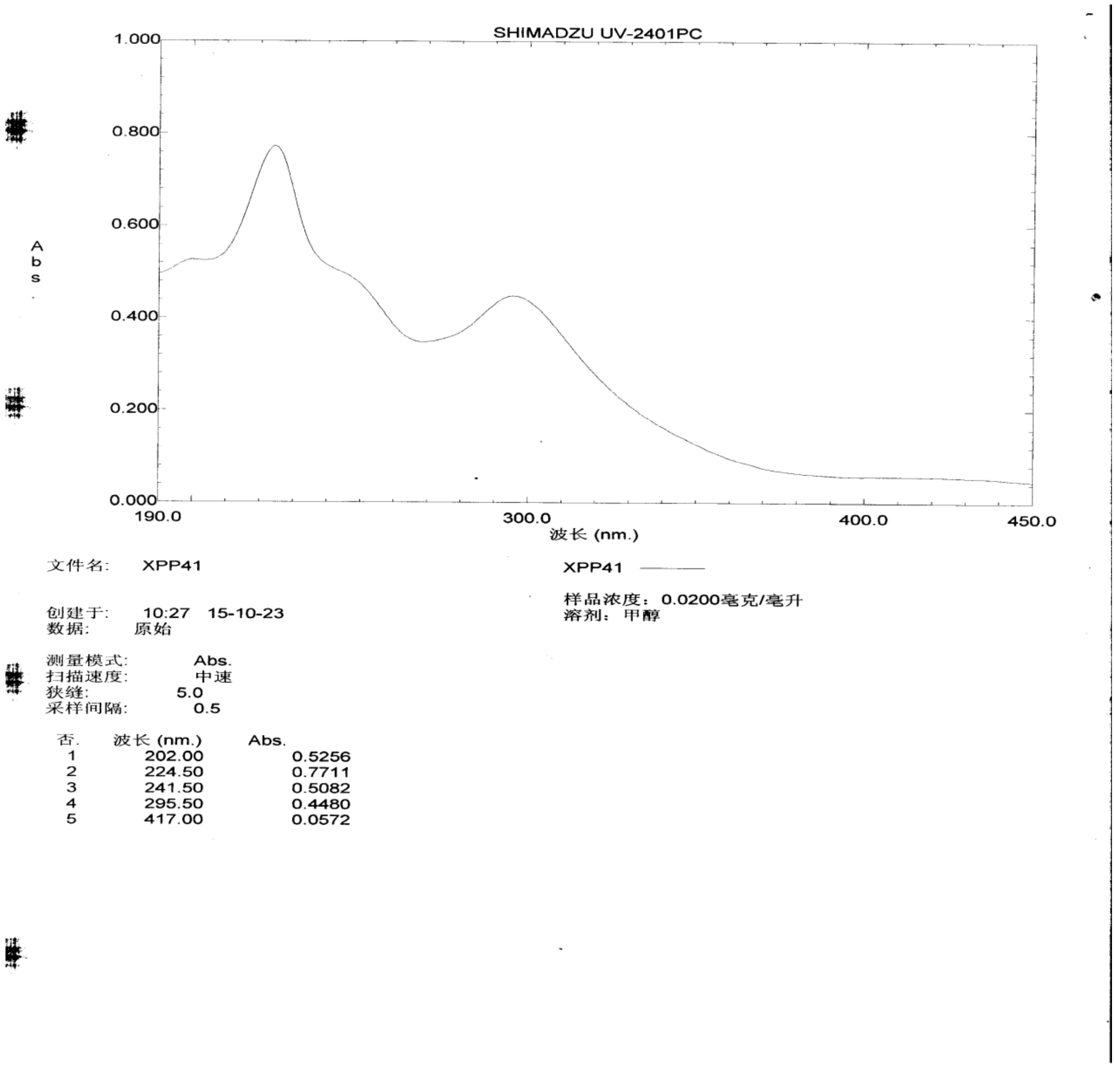

Figure 44S. UV spectrum of (**5**)

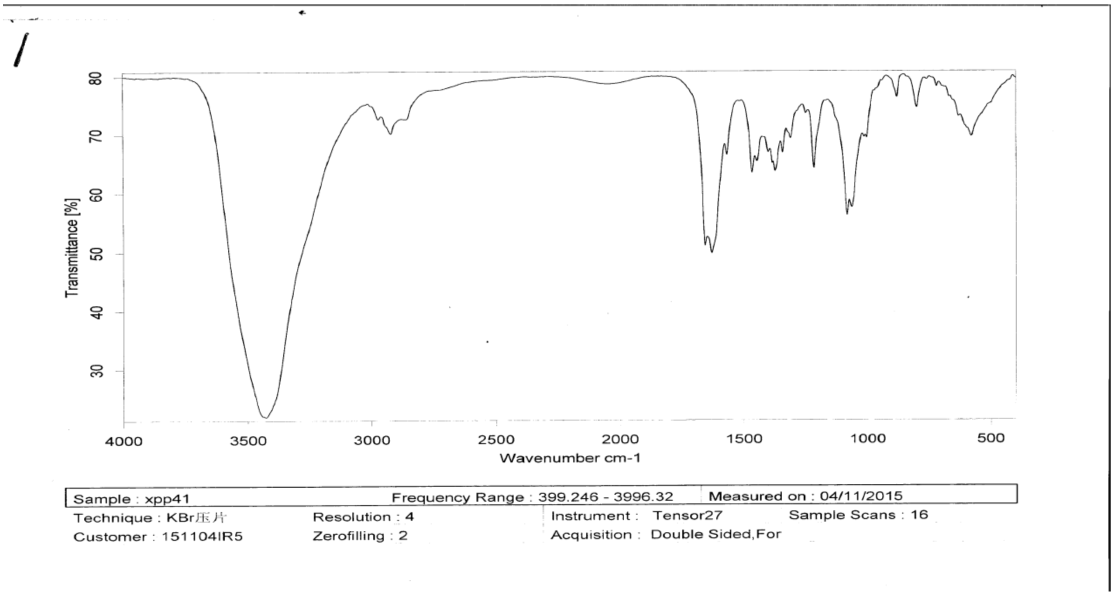

Figure 45S. IR spectrum of (**5**)

Figure 46S-54S. NMR, MS, UV, and IR spectra of compound 6

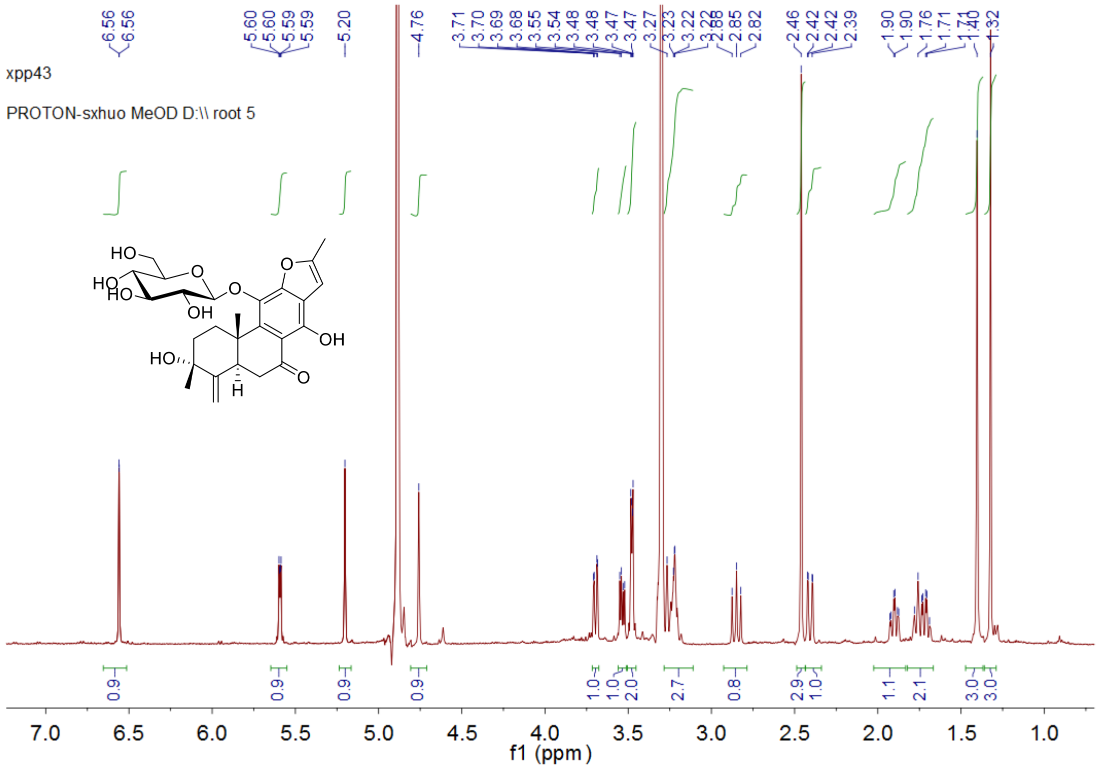

Figure 46S. ^{1}H NMR spectrum of (**6**) recorded in CD_3OD at 600 MHz

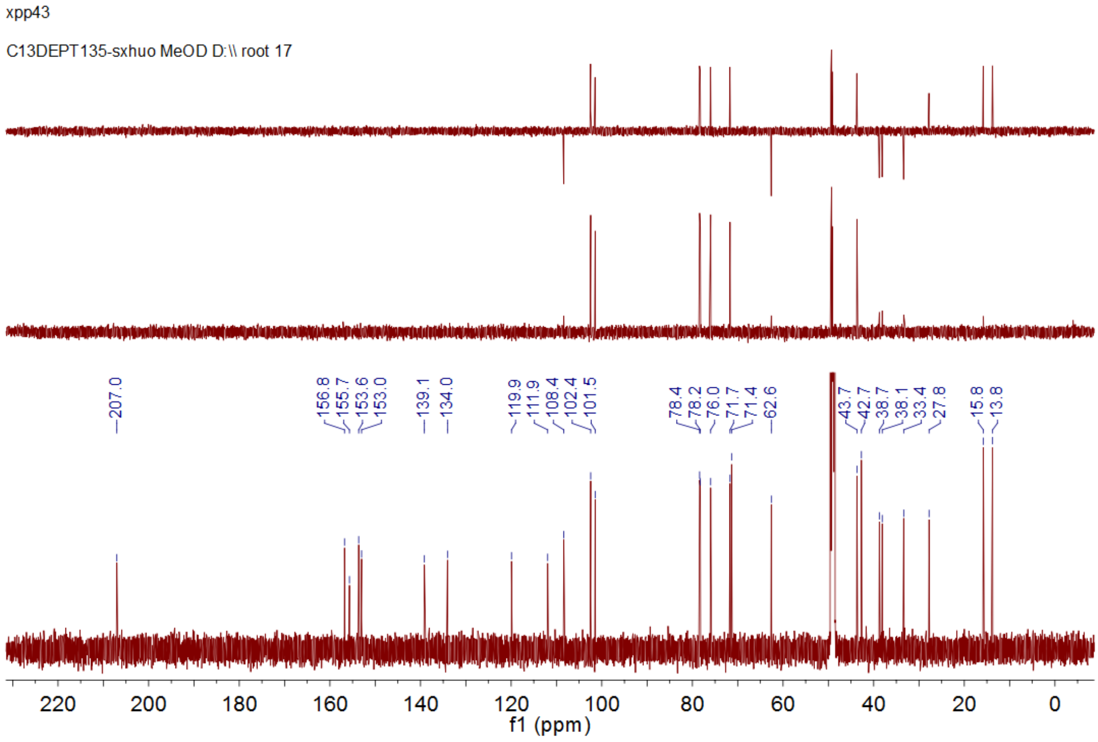

Figure 47S. ^{13}C NMR spectrum of (**6**) recorded in CD_3OD at 150 MHz

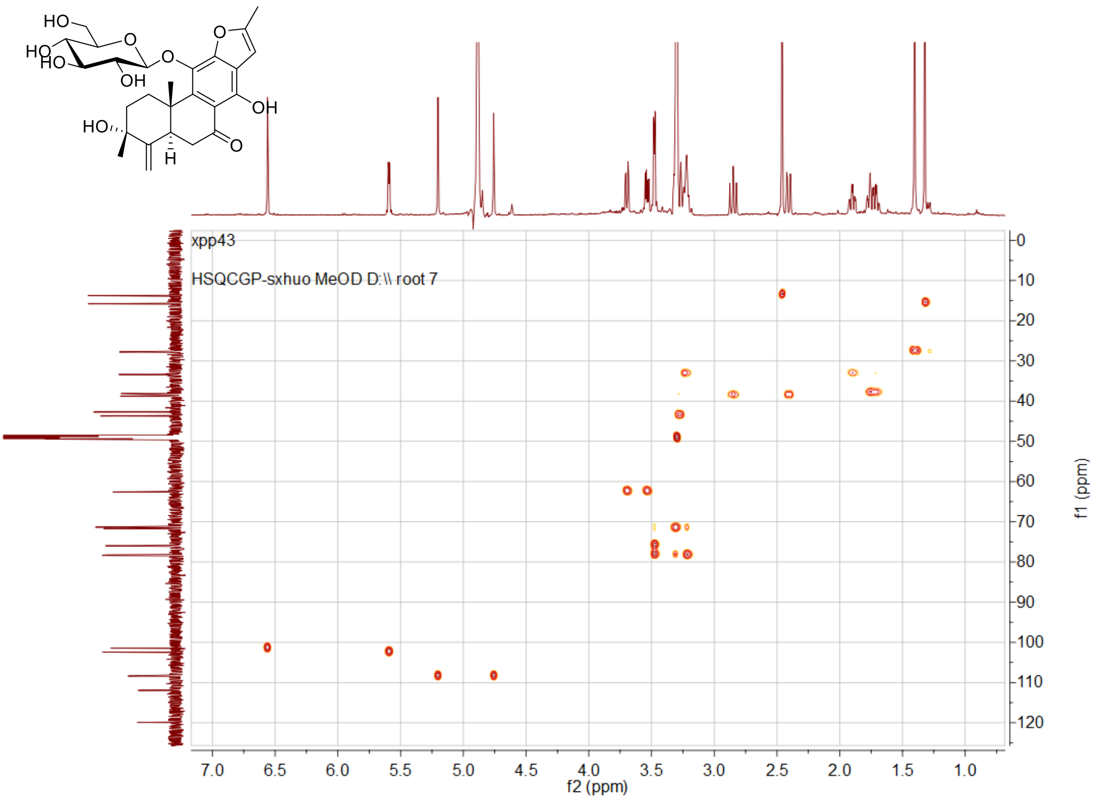

Figure 48S. HSQC spectrum of (**6**) recorded in CD_3OD

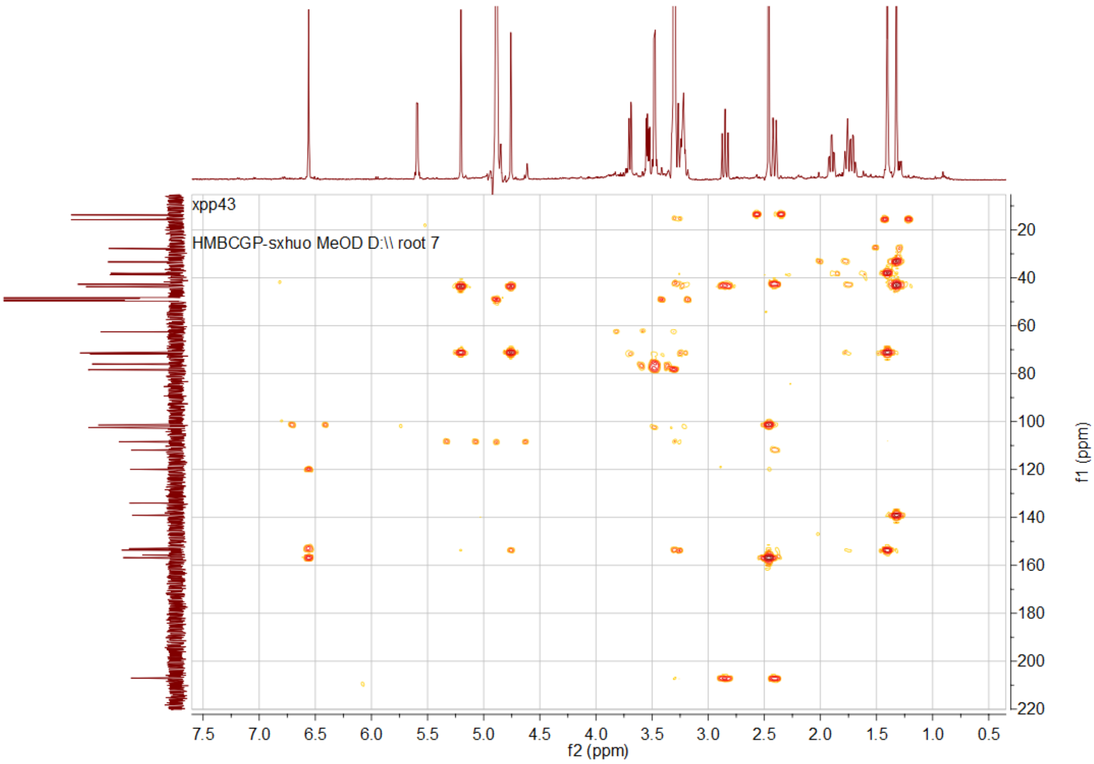

Figure 49S. HMBC spectrum of (**6**) recorded in CD_3OD

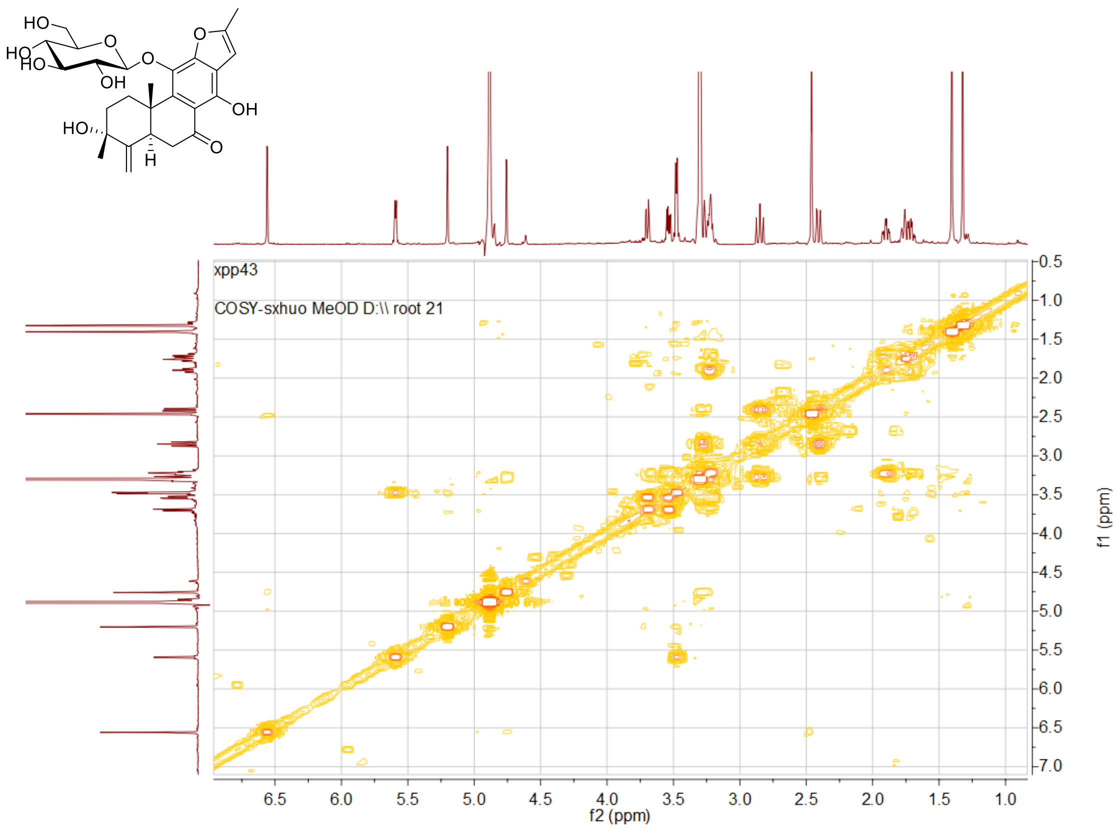

Figure 50S. ^{1}H-^{1}H COSY spectrum of (**6**) recorded in CD_3OD

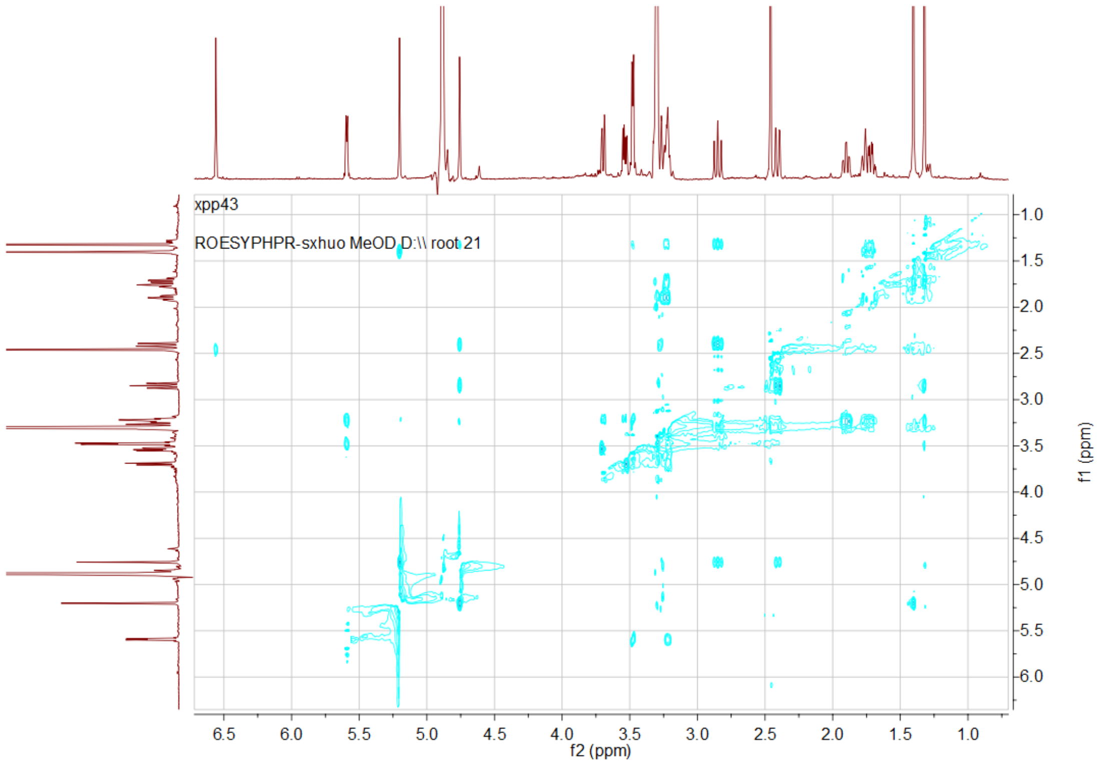

Figure 51S. ROESY spectrum of (**6**) recorded in CD_3OD

Qualitative Analysis Report

**Data Filename**	150630ESINA1.d	**Sample Name**	xpp43
**Sample Type**	Sample	**Position**	
**Instrument Name**	Agilent G6230 TOF MS	**User Name**	KIB
**Acq Method**	ESIN.m	**Acquired Time**	6/30/2015 2:50:08 PM
**IRM Calibration Status**	Success	**DA Method**	ESI.m
**Comment**			

Sample Group **Info.**
Acquisition SW Version 6200 series TOF/6500 series Q-TOF B.05.01 (B5125.2)

User Spectra

Fragmentor Voltage	Collision Energy	Ionization Mode
250	0	ESI

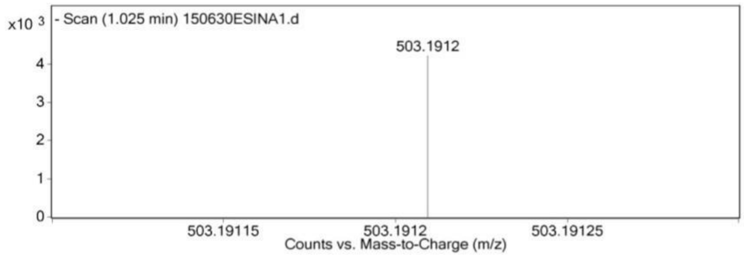

Peak List

m/z	z	Abund	Formula	Ion
112.9856		9606.88		
255.2329	1	10217.37		
283.2642	1	8031.05		
340.1316		1173.53		
341.1391	1	12020.9		
342.142	1	1135.02		
503.1912	1	4215.92	C26 H31 O10	M-
539.1685	1	4698.87		
541.1668	1	906.23		
759.4297	1	992.27		

Formula Calculator Element Limits

Element	Min	Max
C	0	200
H	0	400
O	6	14

Formula Calculator Results

Formula	CalculatedMass	CalculatedMz	Mz	Diff. (mDa)	Diff. (ppm)	DBE
C26 H31 O10	503.1917	503.1923	503.1912	1.2	2.3	11.5000

--- End Of Report ---

Agilent Technologies Page 1 of 1 Printed at: 3:08 PM on: 6/30/2015

Figure 52S. HRESIMS spectrum of (**6**)

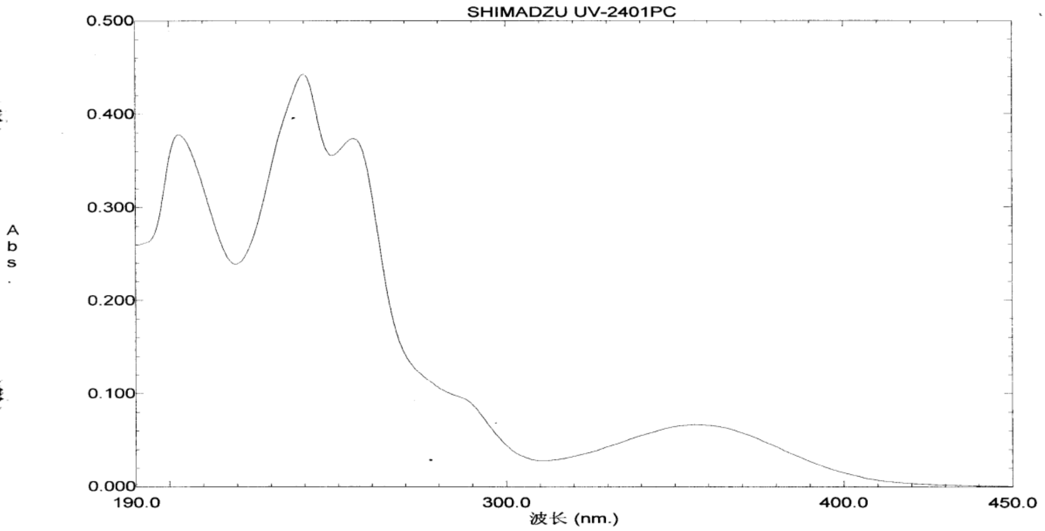

文件名:　XPP43　　XPP43

样品浓度：0.0102毫克/毫升
溶剂：甲醇

创建于:　10:55　15-10-23
数据:　原始

测量模式:　Abs.
扫描速度:　中速
狭缝:　5.0
采样间隔:　0.5

否.	波长 (nm.)	Abs.
1	202.00	0.3756
2	240.00	0.4423
3	254.50	0.3733
4	279.50	0.1076
5	356.00	0.0666

Figure 53S. UV spectrum of (**6**)

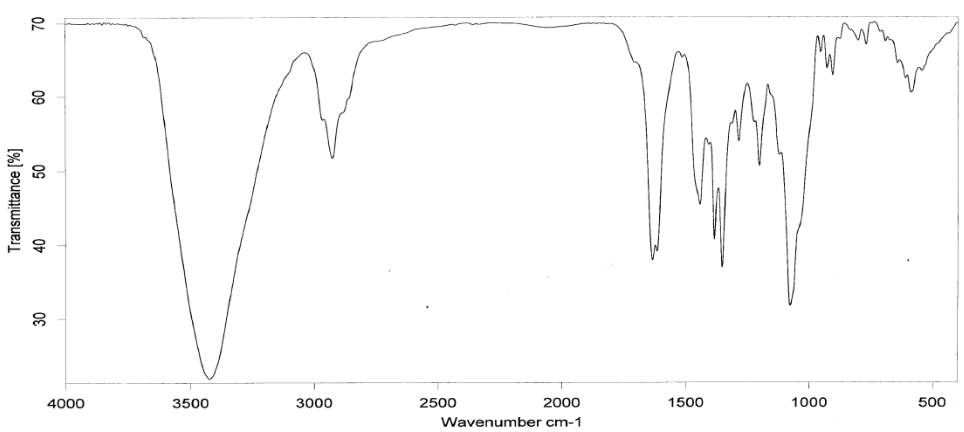

Sample : xpp43	Frequency Range : 399.246 - 3996.32	Measured on : 04/11/2015	
Technique : KBr压片	Resolution : 4	Instrument : Tensor27	Sample Scans : 16
Customer : 151104IR7	Zerofilling : 2	Acquisition : Double Sided,For	

Figure 54S. IR spectrum of (**6**)

Figure 55S-63S. NMR, MS, UV, and IR spectra of compound 7

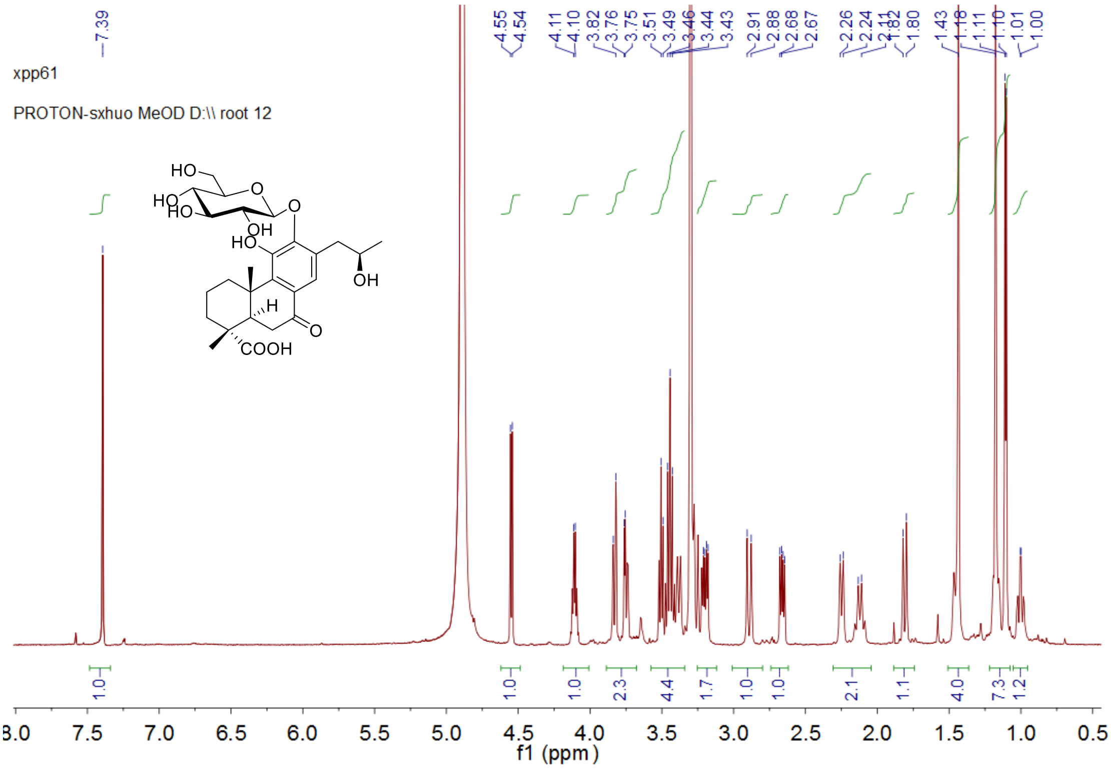

Figure 55S. ^{1}H NMR spectrum of (**7**) recorded in CD_3OD at 600 MHz

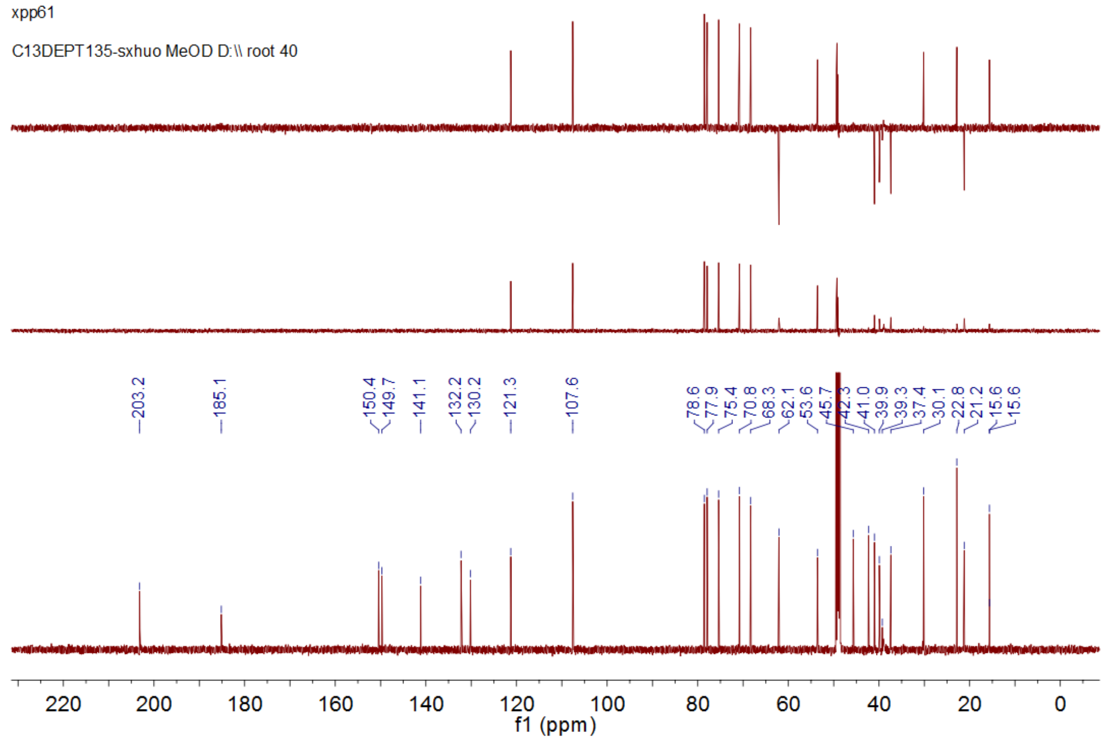

Figure 56S. ^{13}C NMR spectrum of (**7**) recorded in CD_3OD at 150 MHz

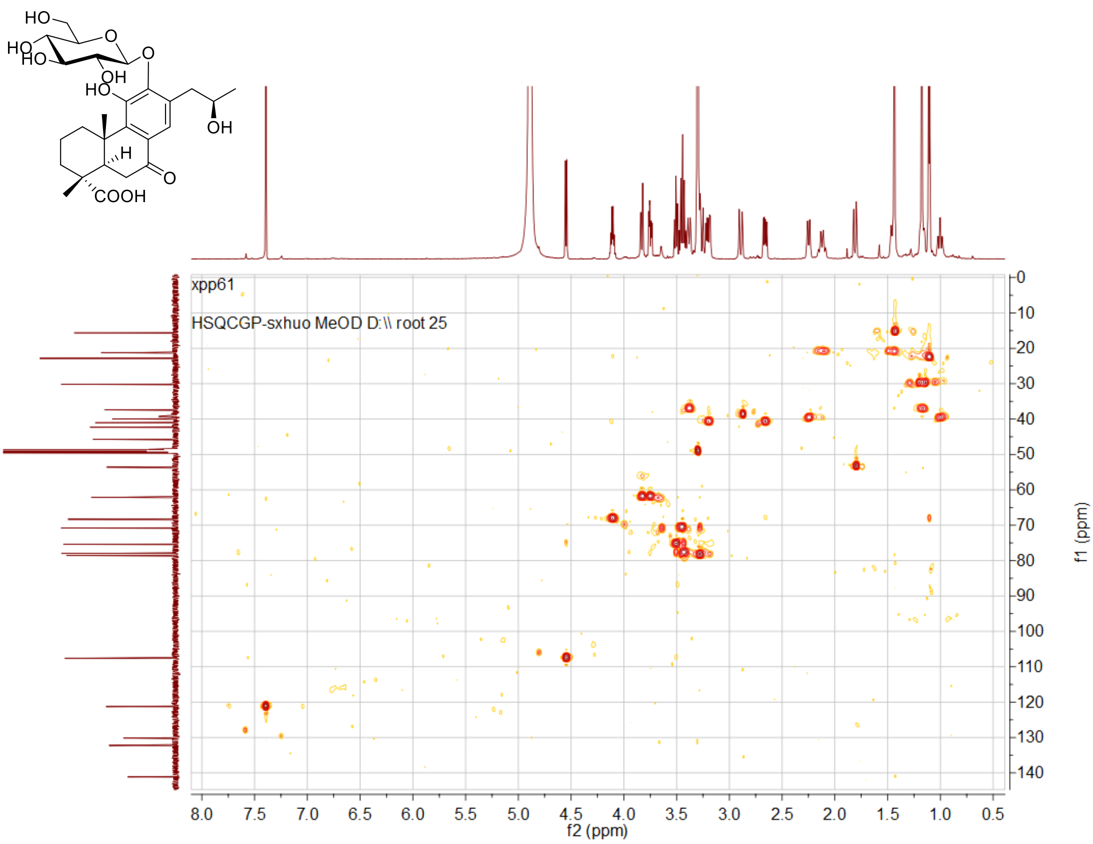

Figure 57S. HSQC spectrum of (**7**) recorded in CD_3OD

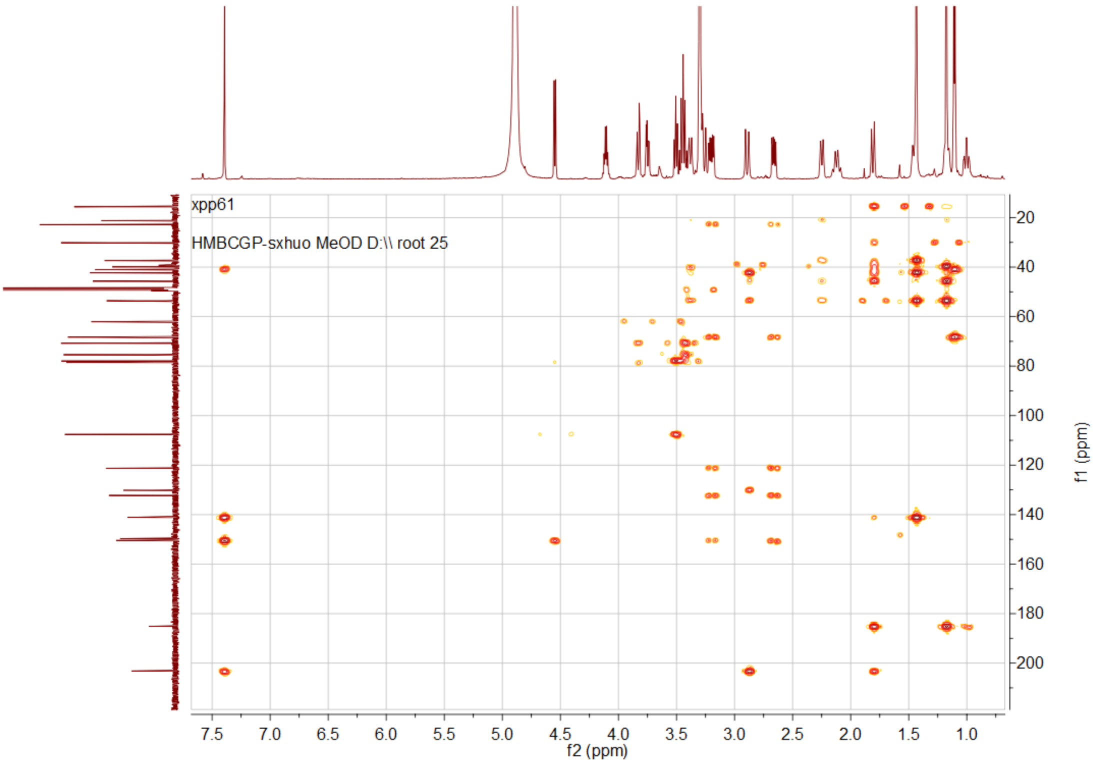

Figure 58S. HMBC spectrum of (**7**) recorded in CD_3OD

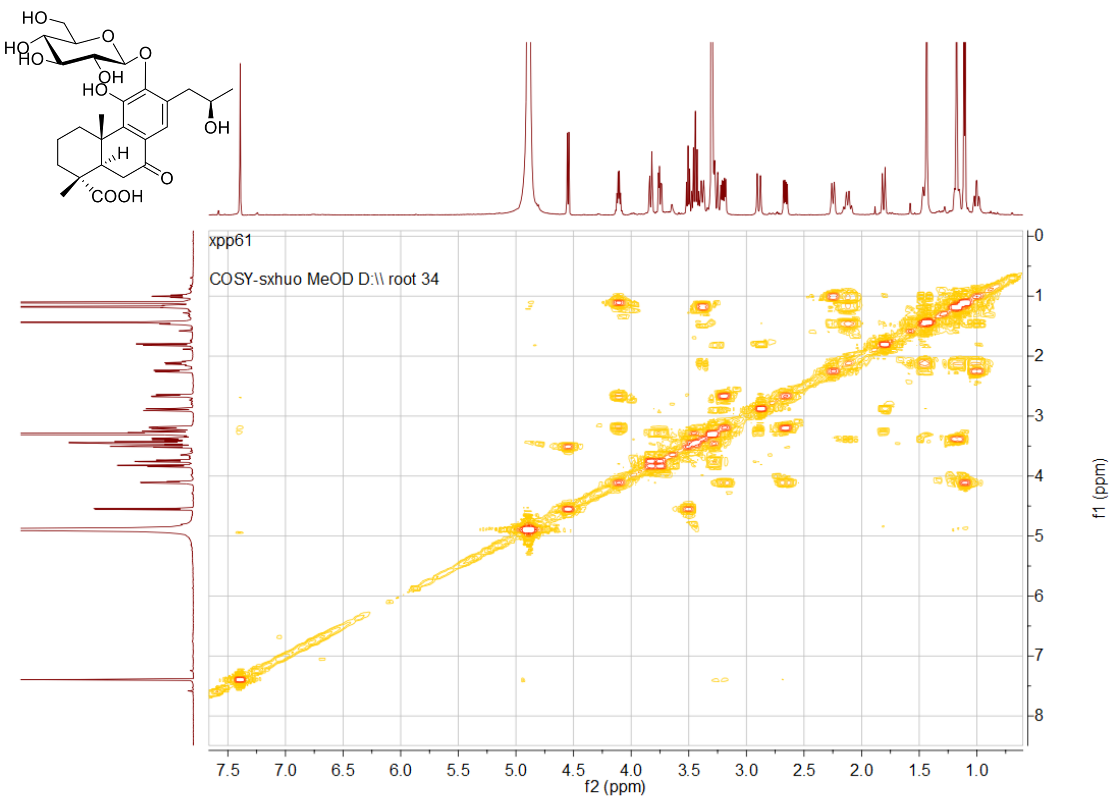

Figure 59S. ^{1}H-^{1}H COSY spectrum of (**7**) recorded in CD_3OD

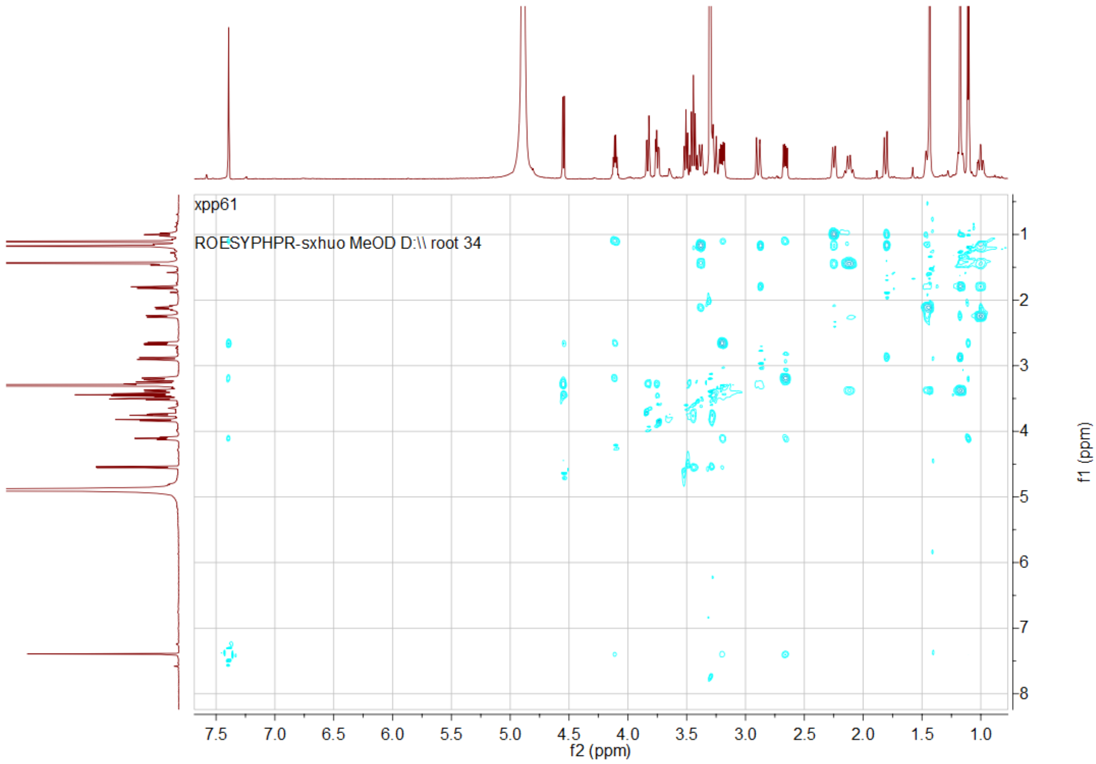

Figure 60S. ROESY spectrum of (**7**) recorded in CD_3OD

Qualitative Analysis Report

**Data Filename**	150630ESINA4.d	**Sample Name**	xpp61
**Sample Type**	Sample	**Position**	
**Instrument Name**	Agilent G6230 TOF MS	**User Name**	KIB
**Acq Method**	ESIN.m	**Acquired Time**	6/30/2015 2:55:24 PM
**IRM Calibration Status**	Success	**DA Method**	ESI.m
**Comment**			

**Sample Group**		**Info.**
**Acquisition SW Version**	6200 series TOF/6500 series Q-TOF B.05.01 (B5125.2)	

User Spectra

Fragmentor Voltage	Collision Energy	Ionization Mode
200	0	ESI

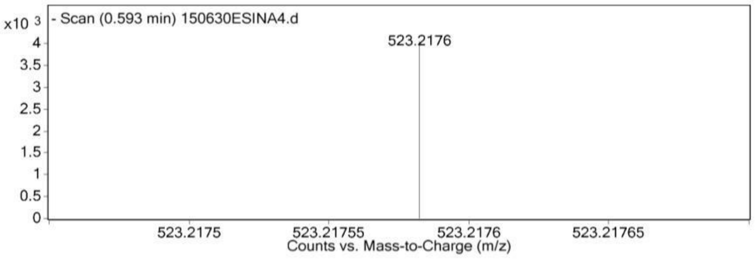

Peak List

m/z	z	Abund	Formula	Ion
112.9856		2944.5		
154.9731		729.01		
255.2327		1018.3		
523.2176	1	4042.03	C26 H35 O11	M-
524.2204	1	589.02	C26 H35 O11	M-
1033.9881	1	208275.13		
1034.9898	1	27024.49		
1035.9902	1	1256.29		
1933.9285	1	27597.17		
1934.9294	1	4732.58		

Formula Calculator Element Limits

Element	Min	Max
C	0	200
H	0	400
O	6	14

Formula Calculator Results

Formula	CalculatedMass	CalculatedMz	Mz	Diff. (mDa)	Diff. (ppm)	DBE
C26 H35 O11	523.2179	523.2185	523.2176	1.0	1.9	9.5000

--- End Of Report ---

Figure 61S. HRESIMS spectrum of (**7**)

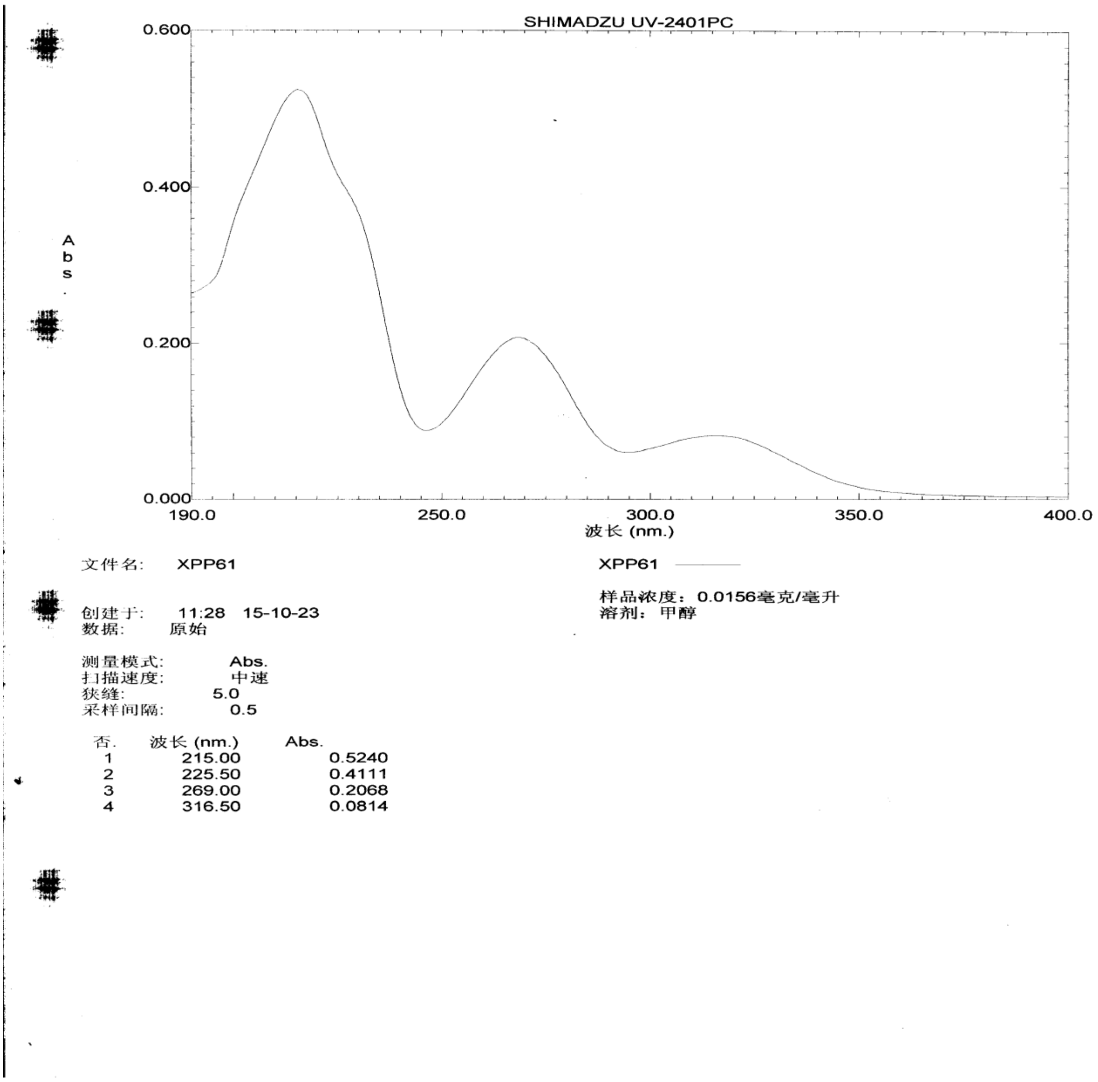

Figure 62S. UV spectrum of (**7**)

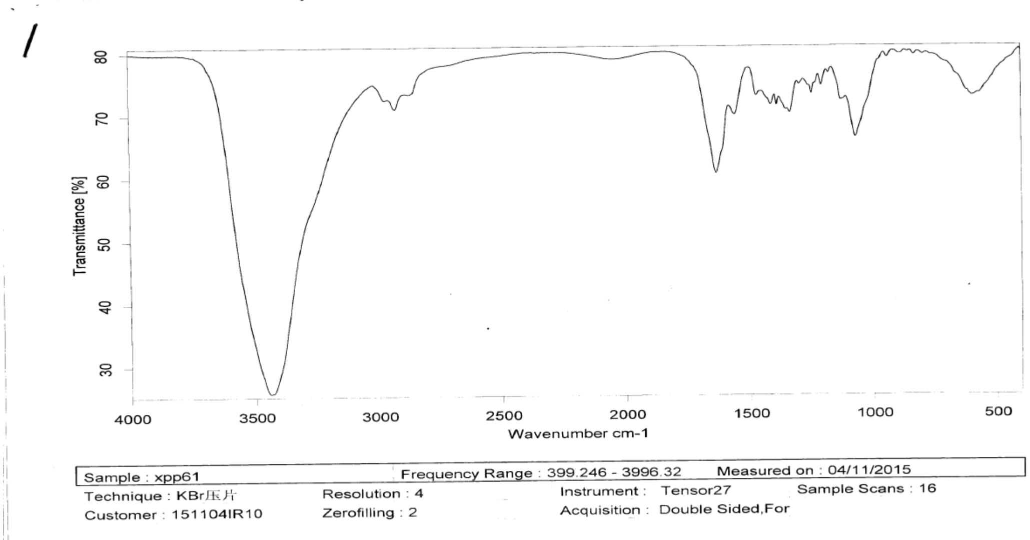

Figure 63S. IR spectrum of (**7**)

Figure 64S-72S. NMR, MS, UV, and IR spectra of compound 8

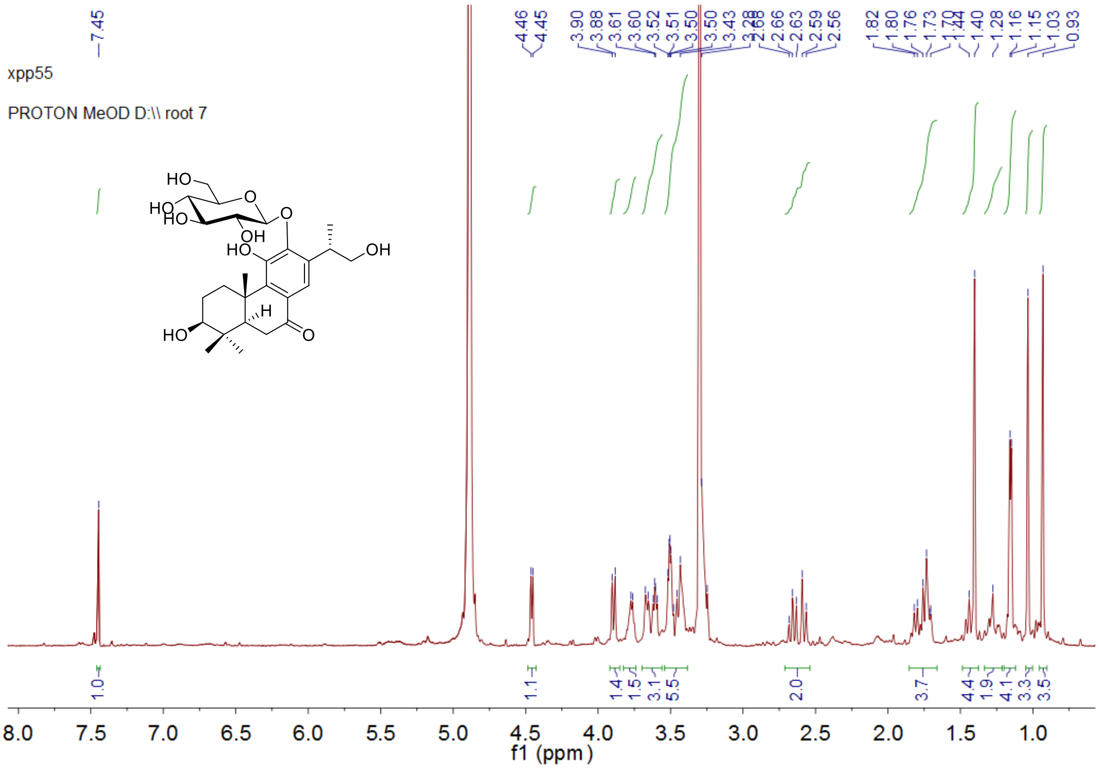

Figure 64S. ^{1}H NMR spectrum of (**8**) recorded in CD_3OD at 600 MHz

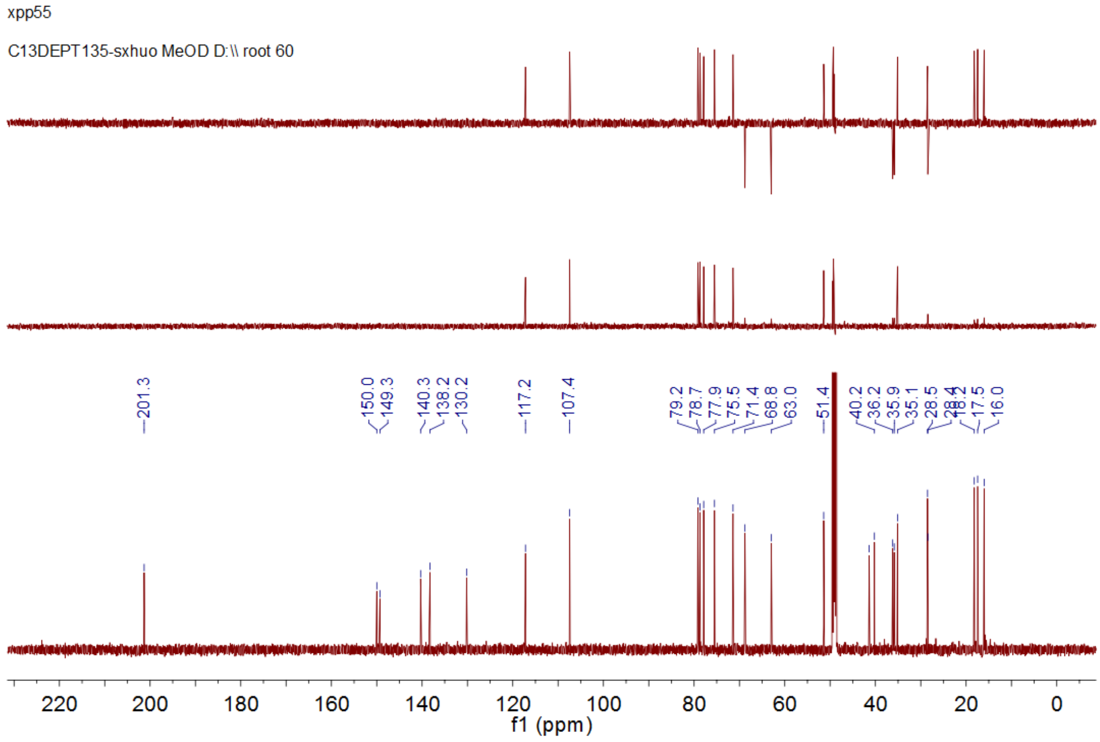

Figure 65S. ^{13}C NMR spectrum of (**8**) recorded in CD_3OD at 150 MHz

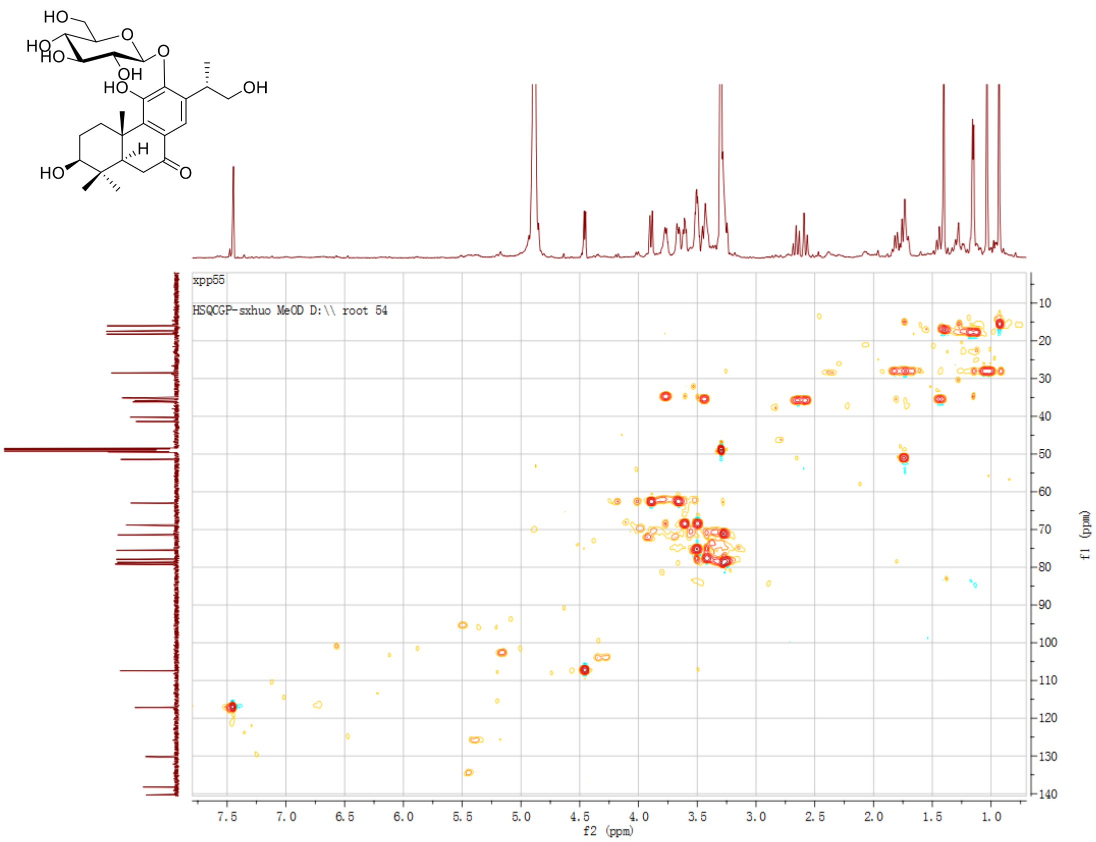

Figure 66S. HSQC spectrum of (**8**) recorded in CD_3OD

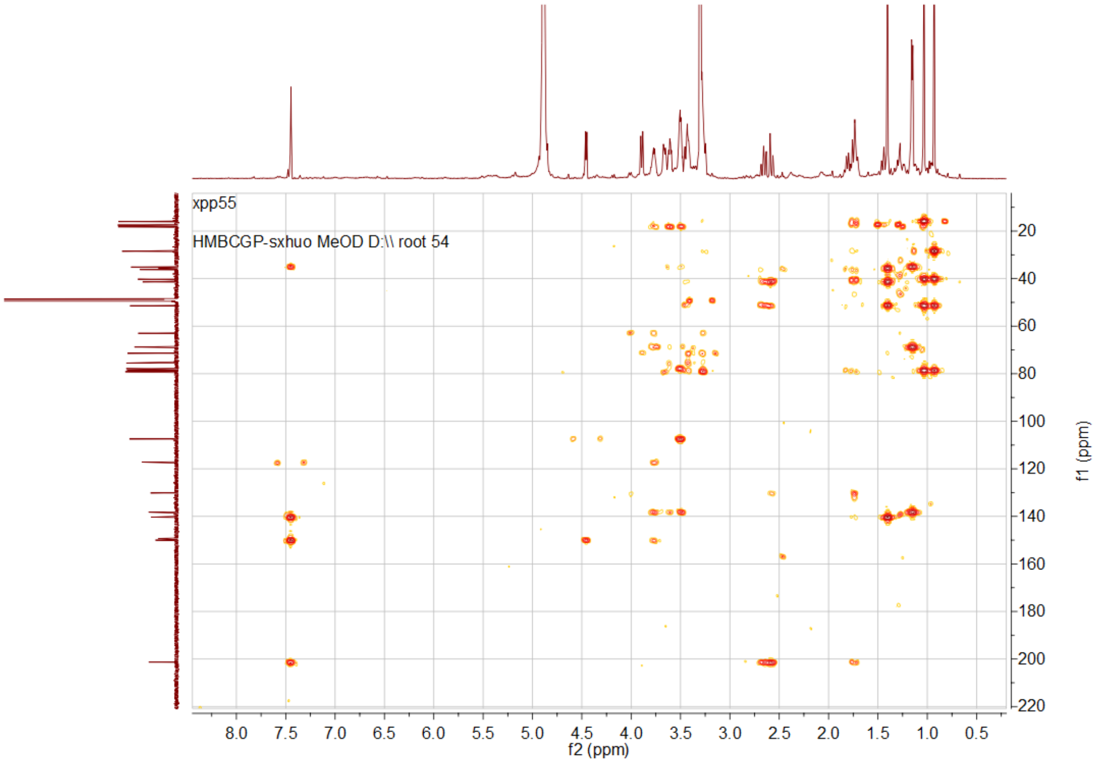

Figure 67S. HMBC spectrum of (**8**) recorded in CD_3OD

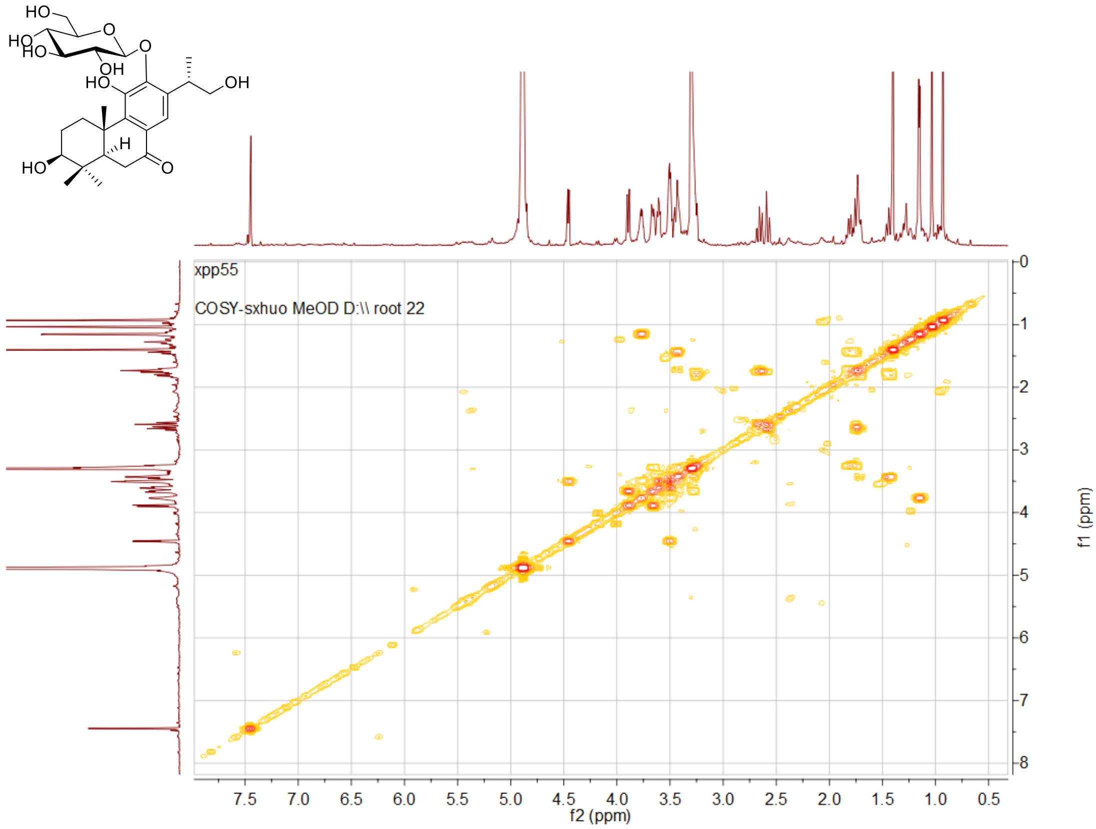

Figure 68S. ^{1}H-^{1}H COESY spectrum of (**8**) recorded in CD_3OD

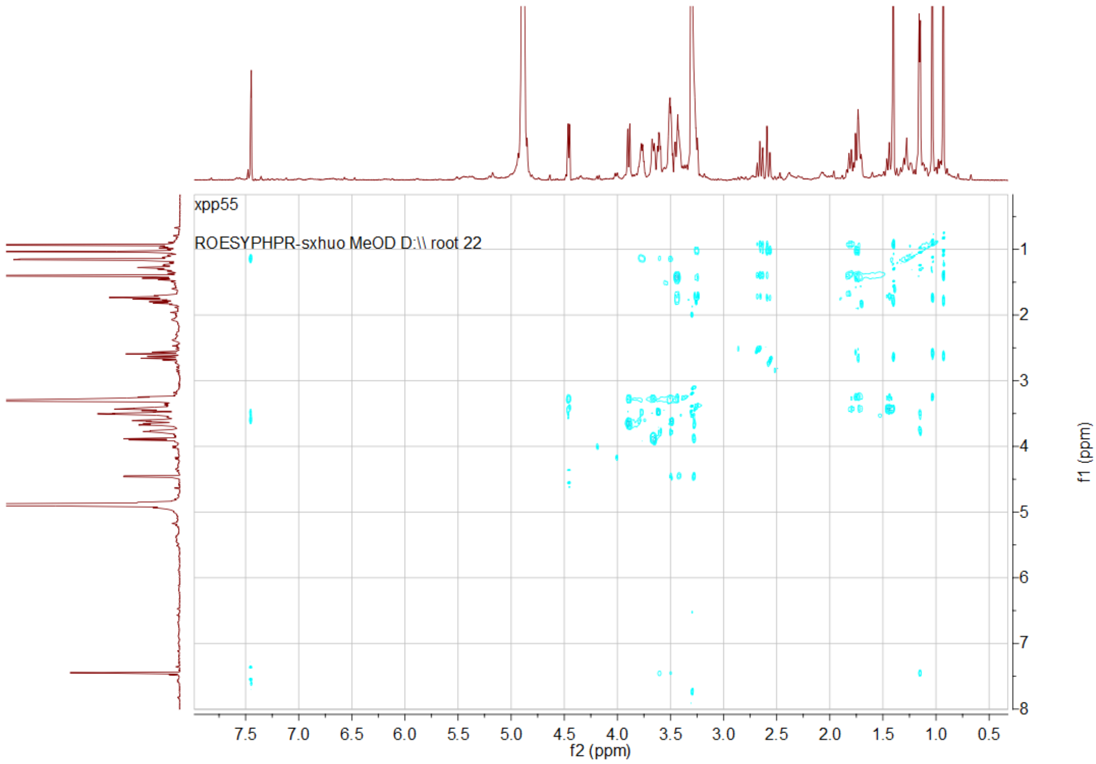

Figure 69S. ROESY spectrum of (**8**) recorded in CD_3OD

Qualitative Analysis Report

**Data Filename**	150630ESINA2.d	**Sample Name**	xpp55
**Sample Type**	Sample	**Position**	
**Instrument Name**	Agilent G6230 TOF MS	**User Name**	KIB
**Acq Method**	ESIN.m	**Acquired Time**	6/30/2015 2:52:26 PM
**IRM Calibration Status**	Success	**DA Method**	ESI.m
**Comment**			

**Sample Group**		**Info.**
**Acquisition SW Version**	6200 series TOF/6500 series Q-TOF B.05.01 (B5125.2)	

User Spectra

Fragmentor Voltage	Collision Energy	Ionization Mode
200	0	ESI

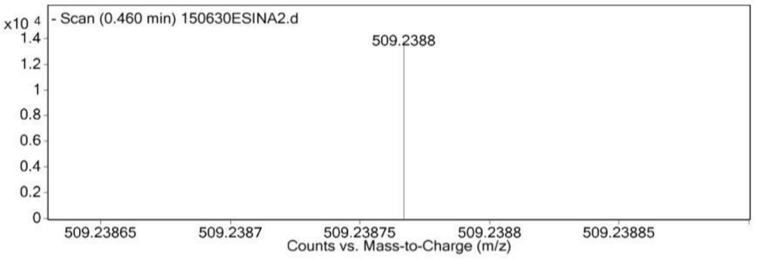

Peak List

m/z	z	Abund	Formula	Ion
112.9856		2889.49		
255.2324	1	4212.37		
283.2636	1	1945.7		
509.2388	1	13739.5	C26 H37 O10	M-
510.2422	1	3087.95	C26 H37 O10	M-
1033.9881	1	191541.73		
1034.9894	1	24188		
1035.9918	1	1152.53		
1933.9294	1	23312.62		
1934.9311	1	3646.12		

Formula Calculator Element Limits

Element	Min	Max
C	0	200
H	0	400
O	6	14

Formula Calculator Results

Formula	CalculatedMass	CalculatedMz	Mz	Diff. (mDa)	Diff. (ppm)	DBE
C26 H37 O10	509.2387	509.2392	509.2388	0.4	0.8	8.5000

--- End Of Report ---

Agilent Technologies Page 1 of 1 Printed at: 3:06 PM on: 6/30/2015

Figure 70S. HRESIMS spectrum of (**8**)

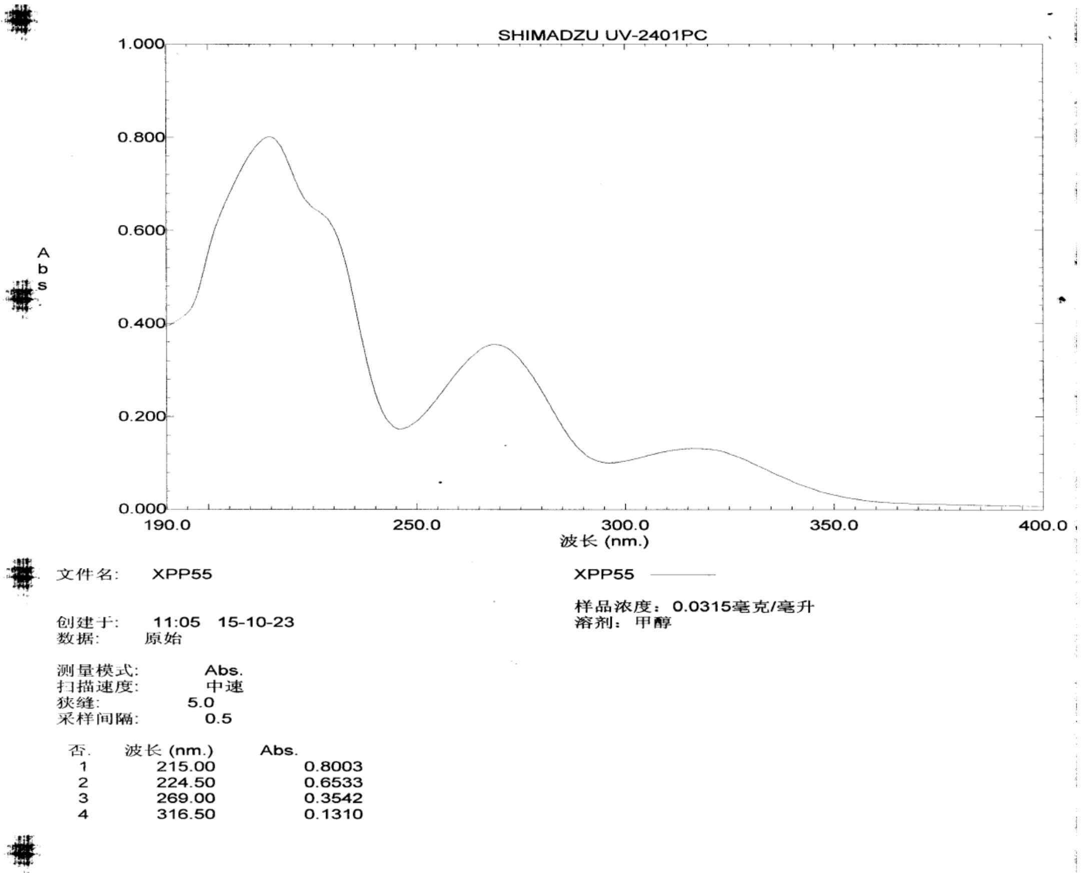

Figure 71S. UV spectrum of (**8**)

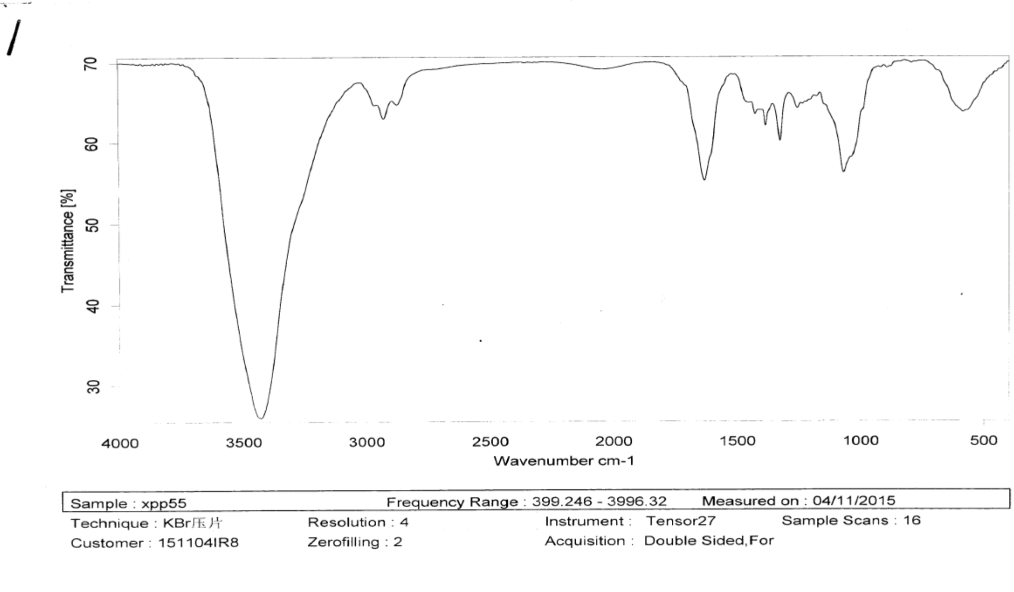

Figure 72S. IR spectrum of (**8**)

Figure 73S-81S. NMR, MS, UV, and IR spectra of compound 9

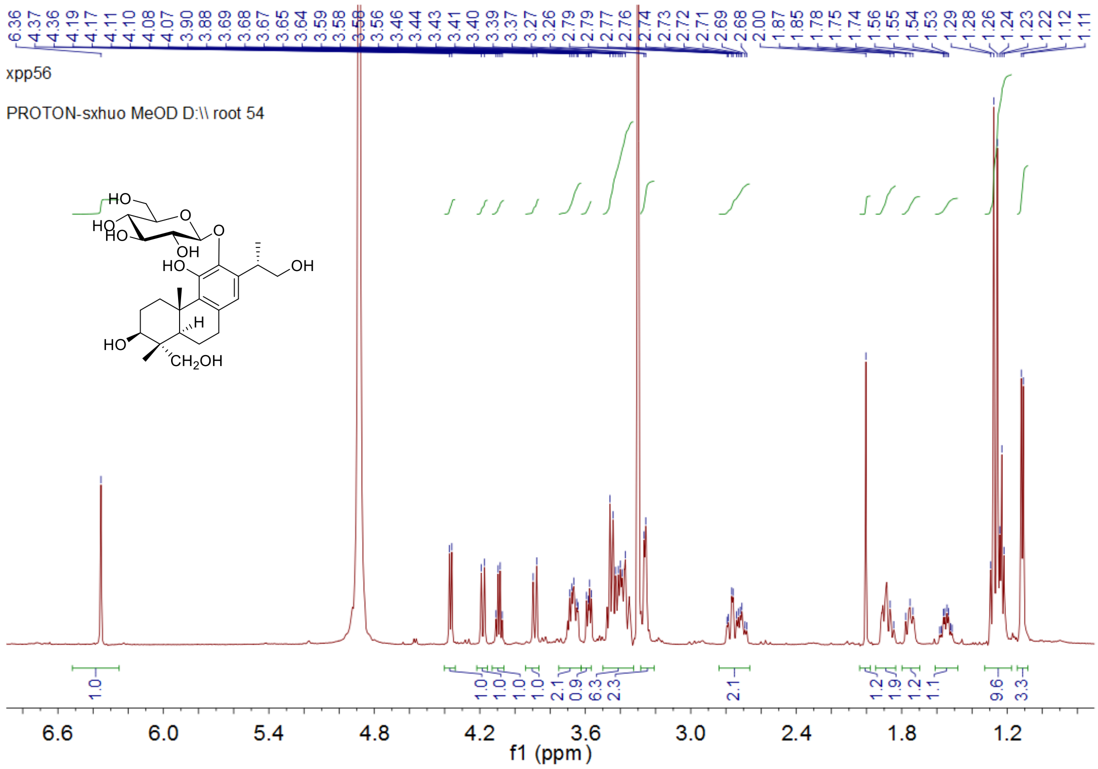

Figure 73S. ^{1}H NMR spectrum of (**9**) recorded in CD_3OD at 600 MHz

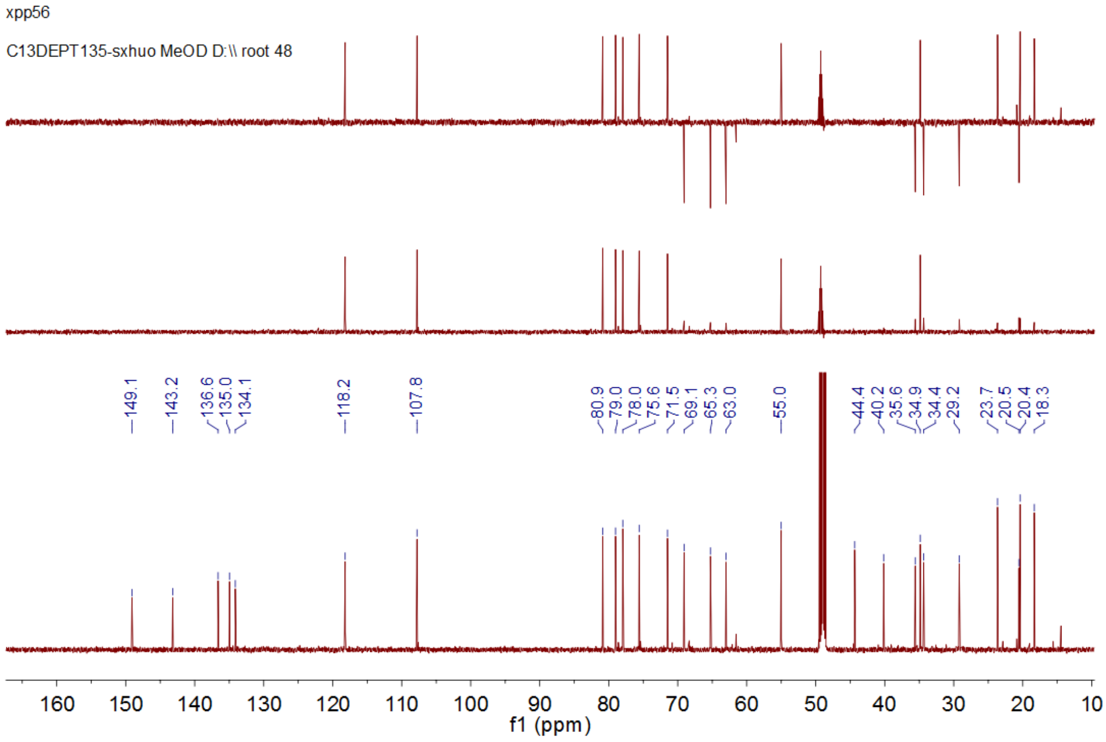

Figure 74S. ^{13}C NMR spectrum of (**9**) recorded in CD_3OD at 150 MHz

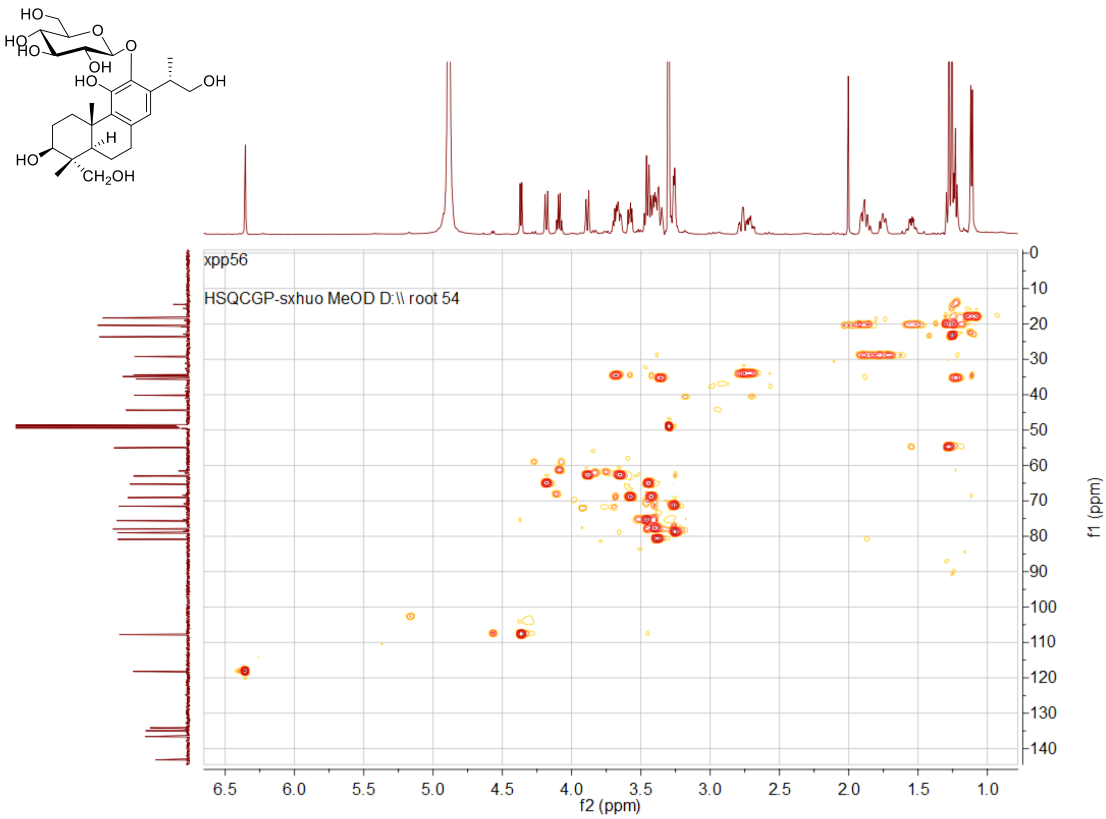

Figure 75S. HSQC spectrum of (**9**) recorded in CD_3OD

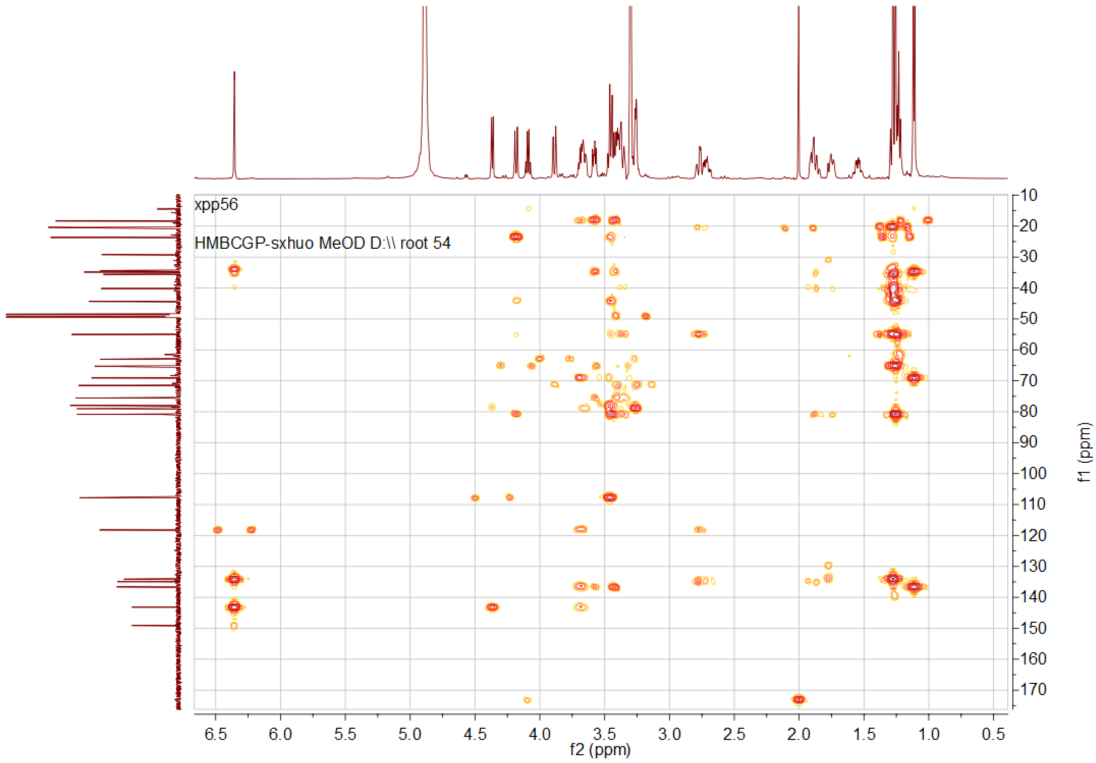

Figure 76S. HMBC spectrum of (**9**) recorded in CD_3OD

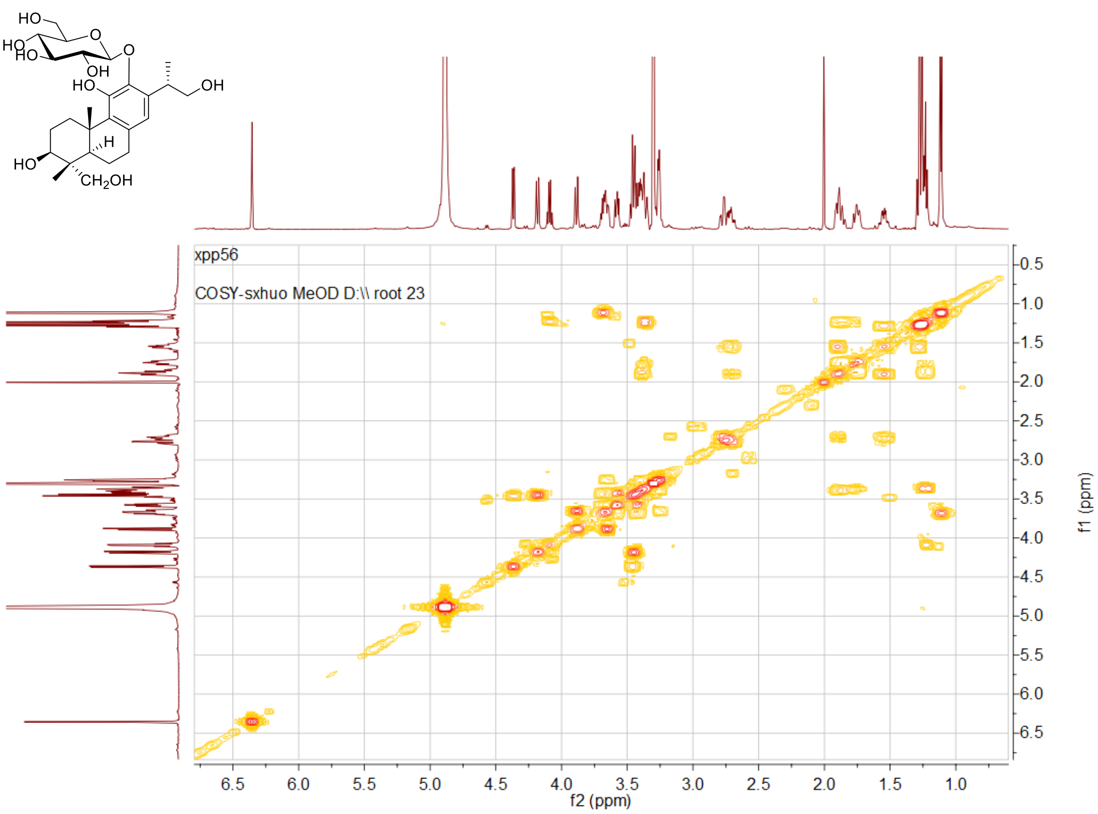

Figure 77S. ^{1}H-^{1}H COSY spectrum of (**9**) recorded in CD_3OD

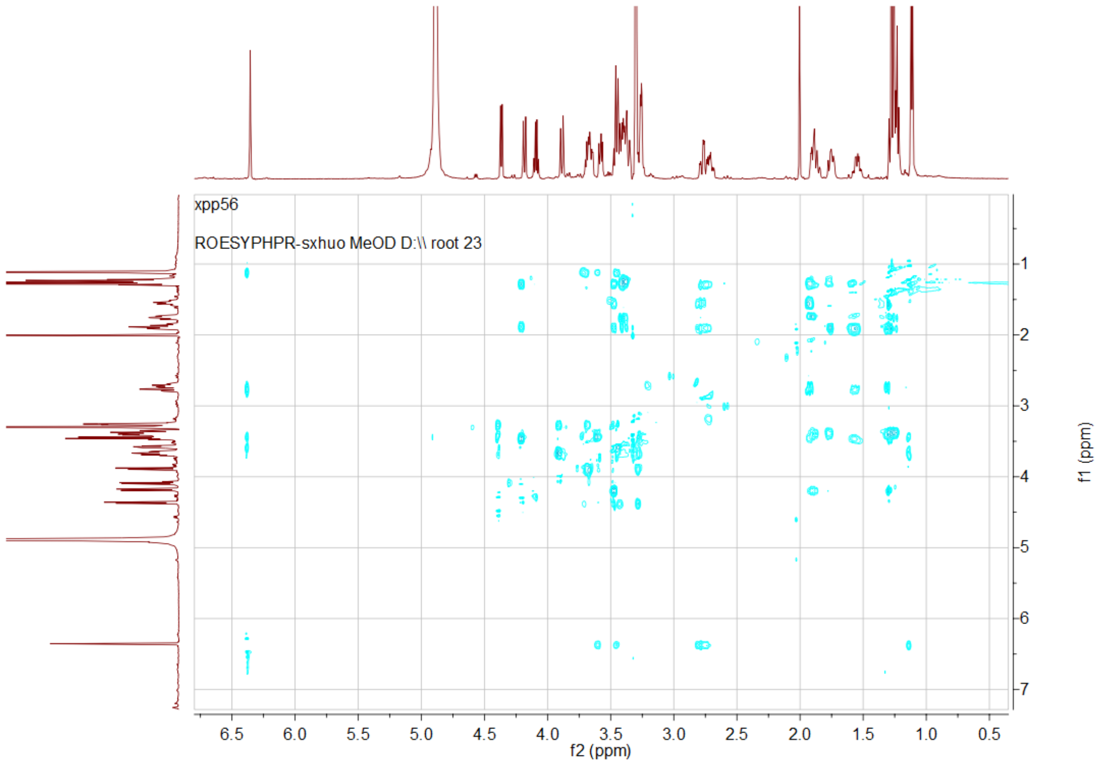

Figure 78S. ROESY spectrum of (**9**) recorded in CD_3OD

Qualitative Analysis Report

**Data Filename**	150615ESINA2.d	**Sample Name**	xpp56
**Sample Type**	Sample	**Position**	
**Instrument Name**	Agilent G6230 TOF MS	**User Name**	KIB
**Acq Method**	ESIN.m	**Acquired Time**	6/15/2015 9:57:17 AM
**IRM Calibration Status**	Success	**DA Method**	ESI.m
**Comment**			

**Sample Group**		**Info.**
**Acquisition SW Version**	6200 series TOF/6500 series Q-TOF B.05.01 (B5125.2)	

User Spectra

Fragmentor Voltage	Collision Energy	Ionization Mode
200	0	ESI

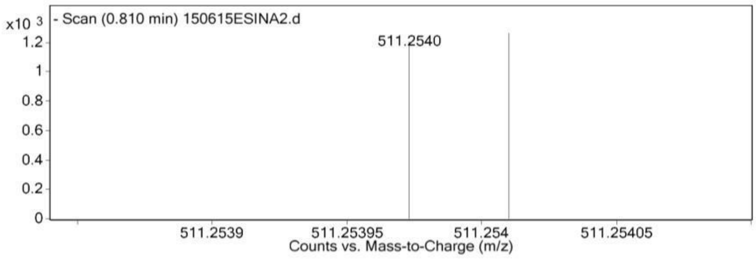

Peak List

m/z	z	Abund	Formula	Ion
112.9856		2878.78		
154.9737		759.05		
248.9606		589.55		
255.2326		401.66		
511.254		1202.14	C26 H39 O10	M-
1033.9881	1	168531.73		
1034.9899	1	20274.93		
1035.9911	1	942.03		
1933.9293	1	21412.95		
1934.9301	1	3323.8		

Formula Calculator Element Limits

Element	Min	Max
C	0	200
H	0	400
O	6	12

Formula Calculator Results

Formula	CalculatedMass	CalculatedMz	Mz	Diff. (mDa)	Diff. (ppm)	DBE
C26 H39 O10	511.2543	511.2549	511.2540	0.9	1.8	7.5000

--- End Of Report ---

Figure 79S. HRESIMS spectrum of (**9**)

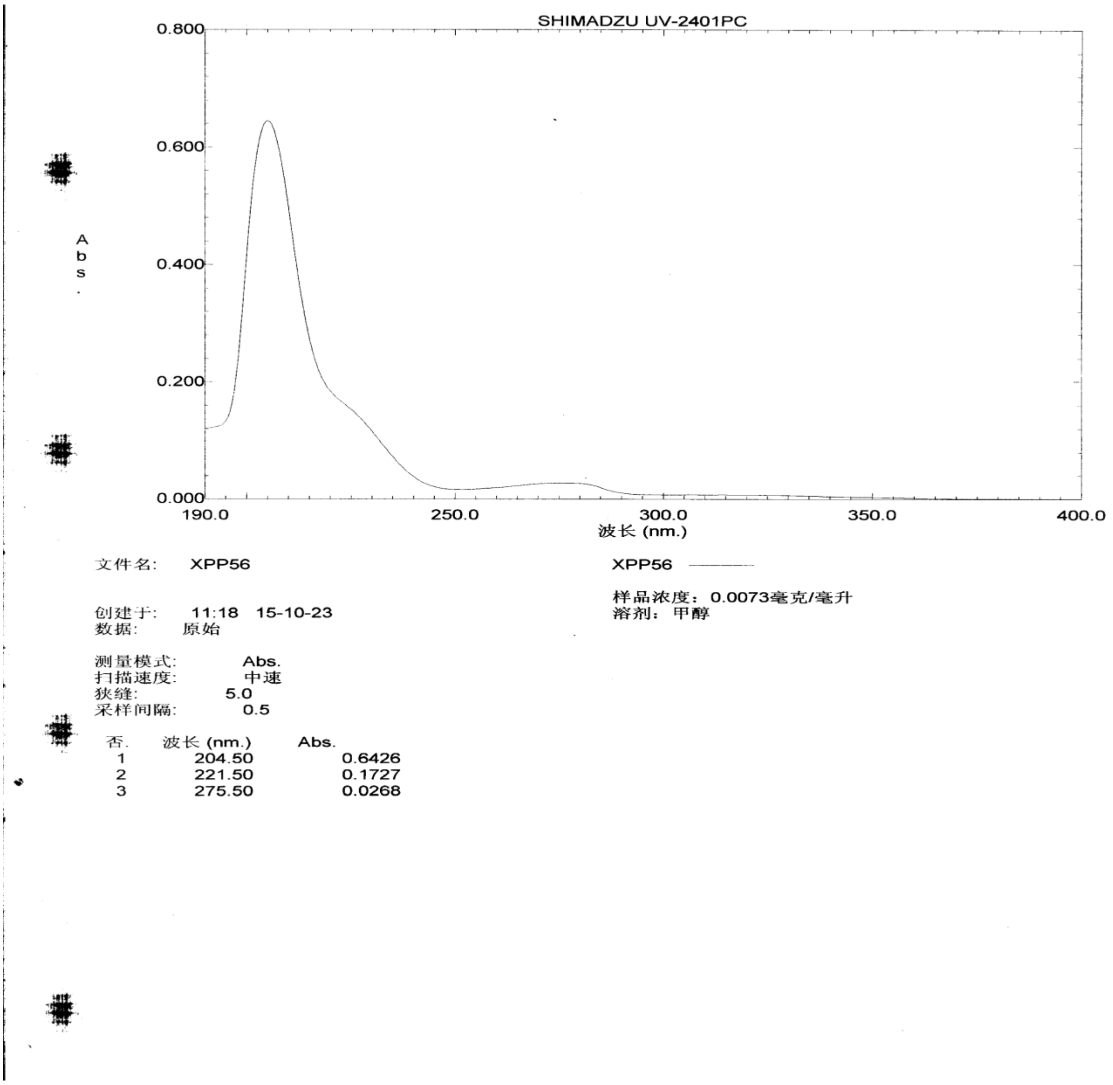

Figure 80S. UV spectrum of (**9**)

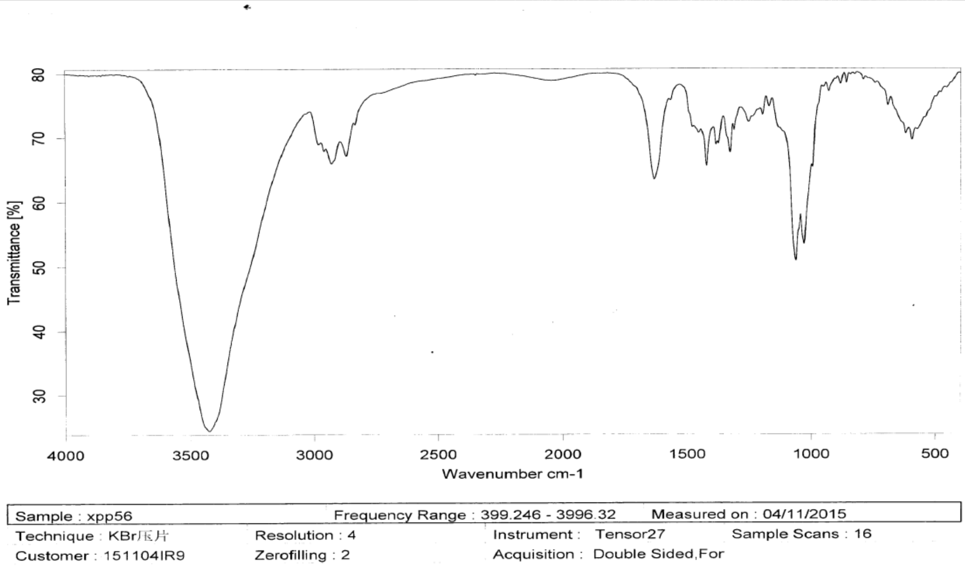

Figure 81S. IR spectrum of (**9**)

Figure 82S-90S. NMR, MS, UV, and IR spectra of compound 10

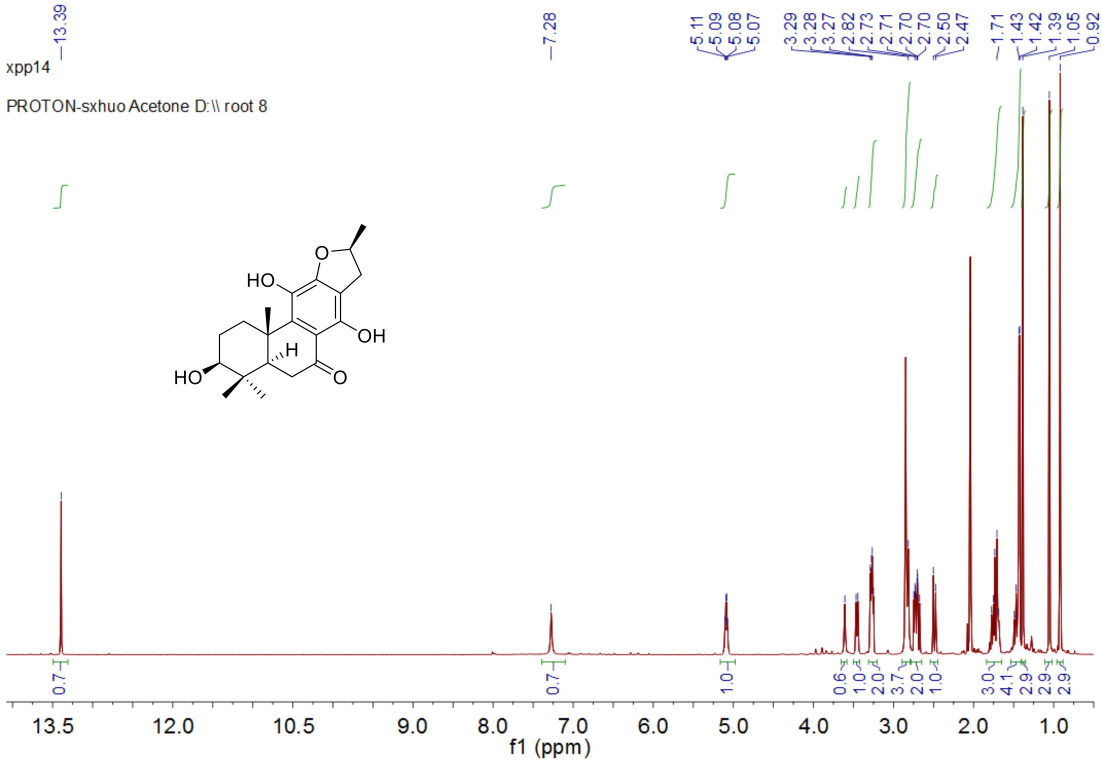

Figure 82S. ^{1}H NMR spectrum of (**10**) recorded in acetone-d_6 at 600 MHz

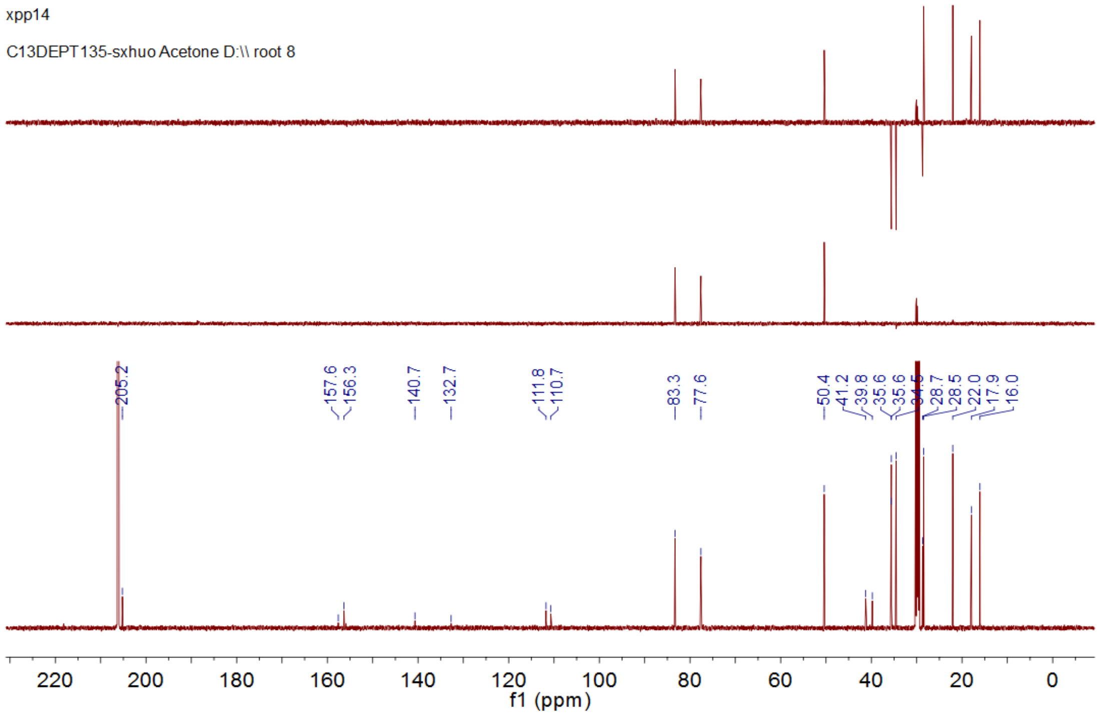

Figure 83S. ^{13}C NMR spectrum of (**10**) recorded in acetone-d_6 at 150 MHz

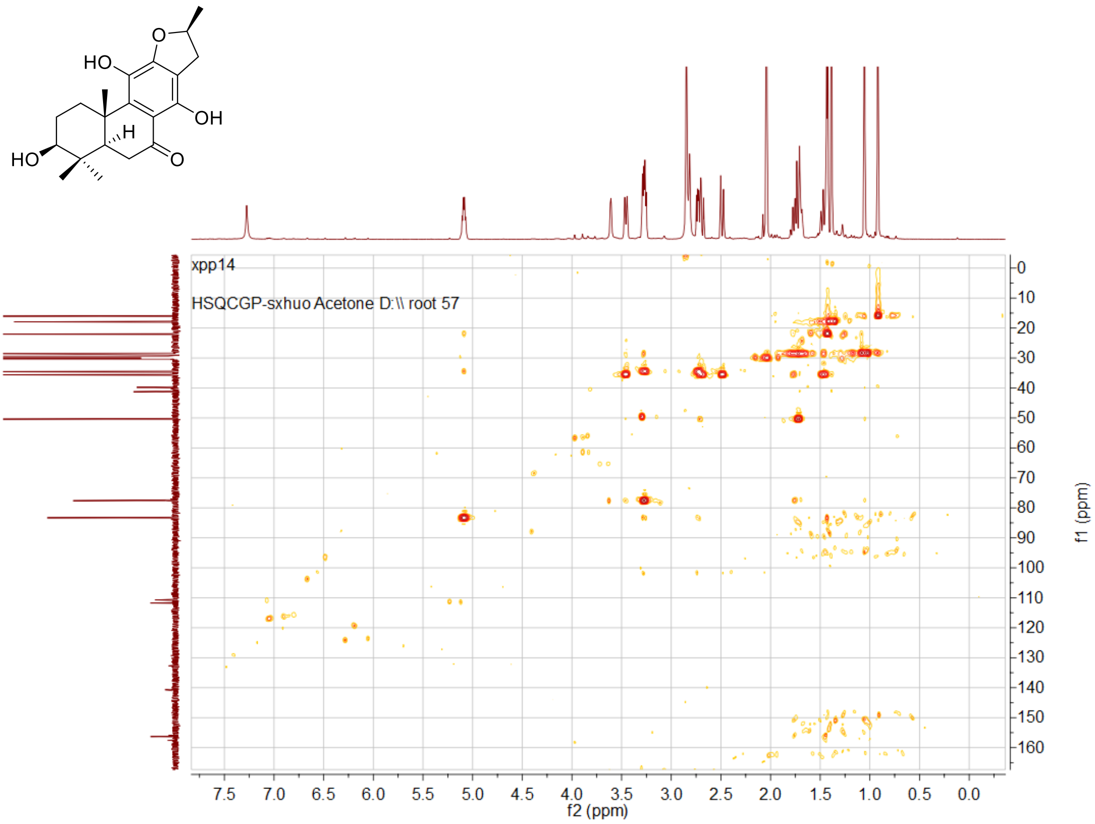

Figure 84S. HSQC spectrum of (**10**) recorded in acetone-d_6

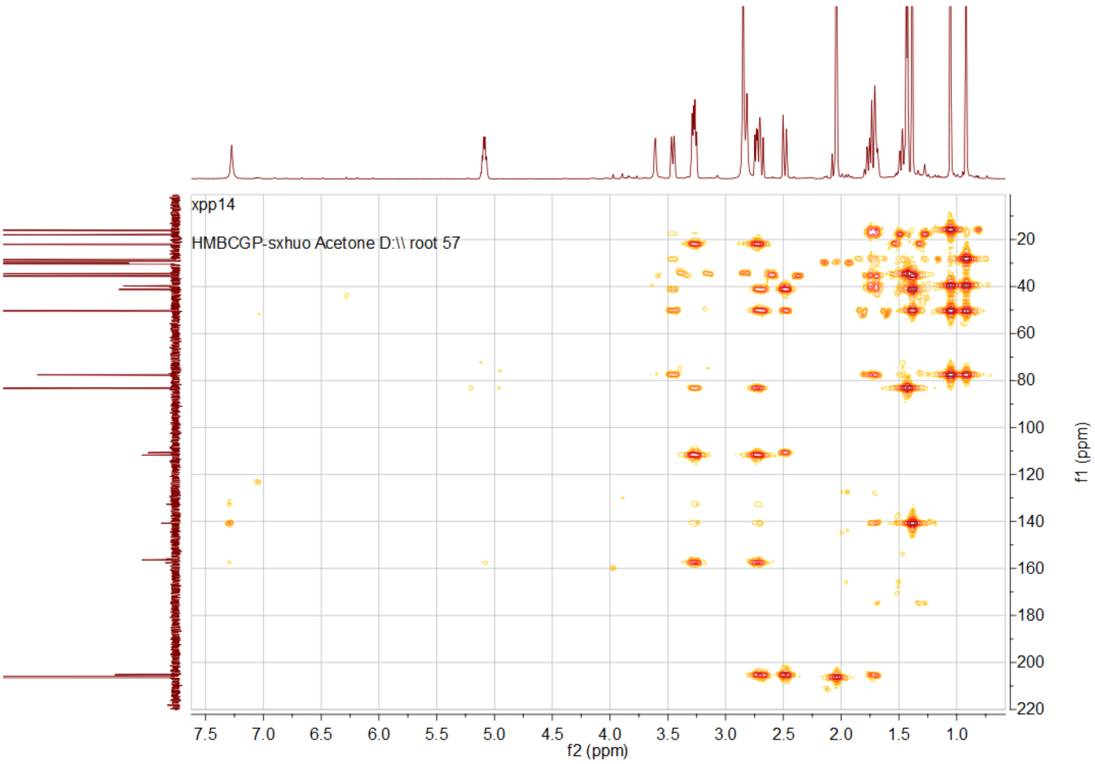

Figure 85S. HMBC spectrum of (**10**) recorded in acetone-d_6

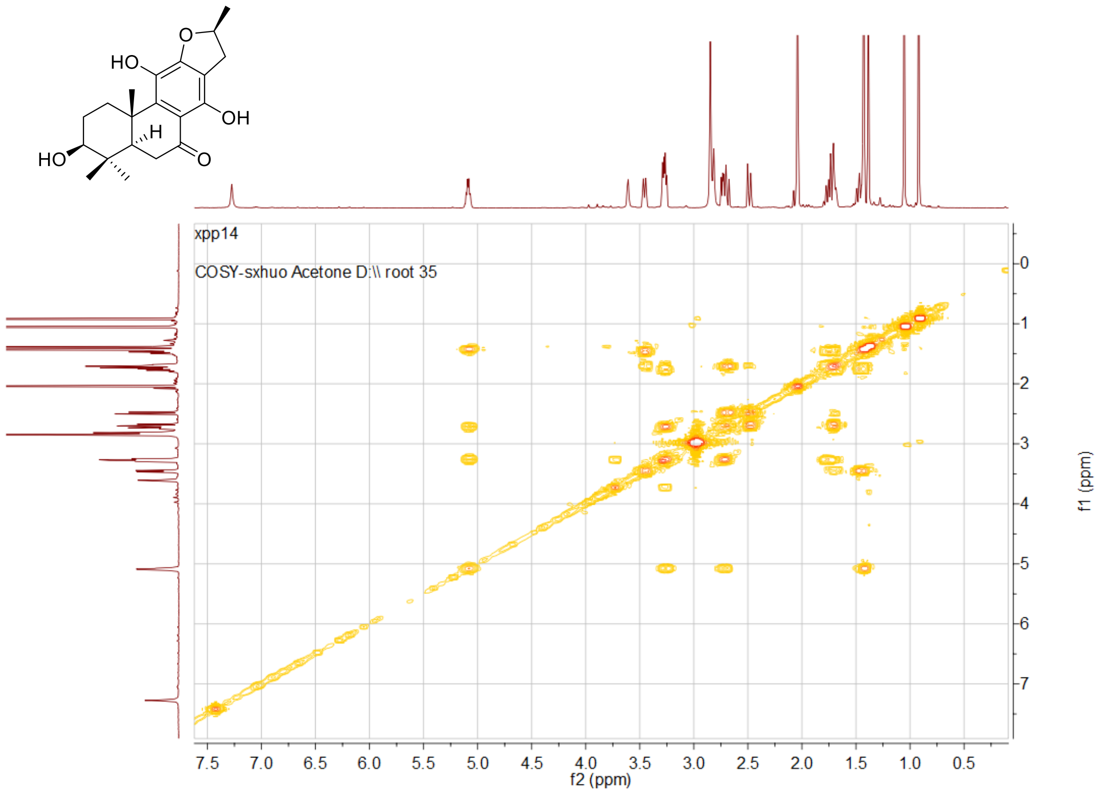

Figure 86S. ^{1}H-^{1}H COSY spectrum of (**10**) recorded in acetone-d_6

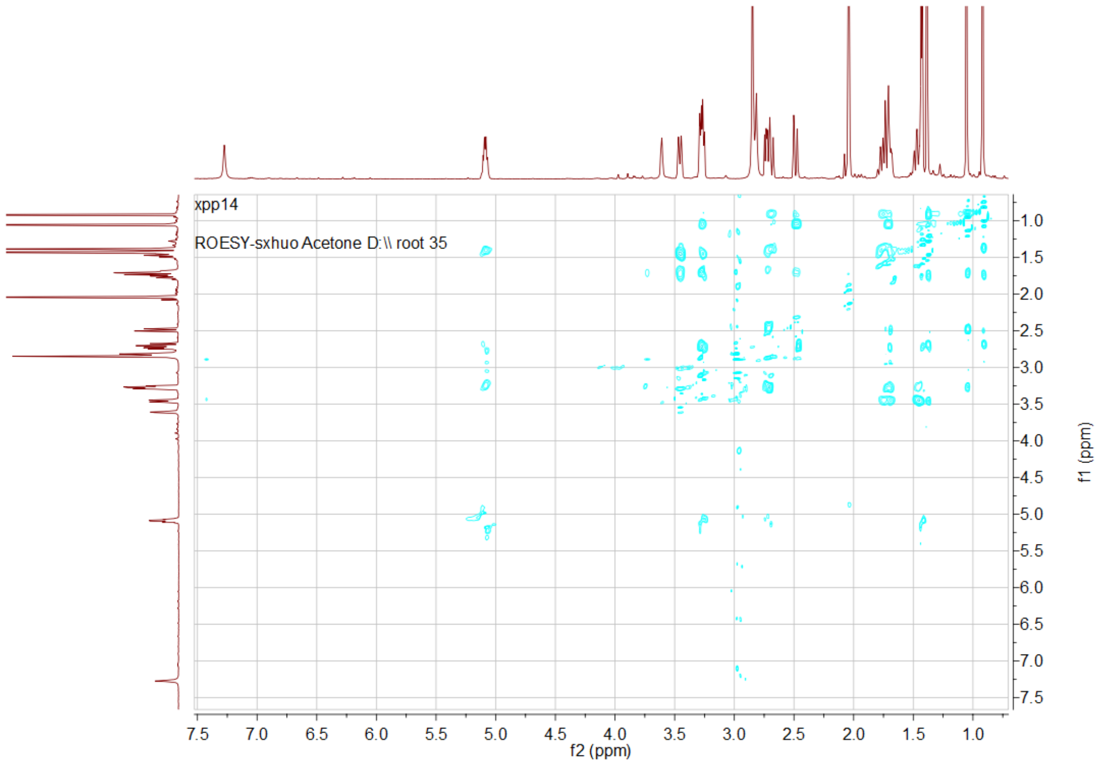

Figure 87S. ROESY spectrum of (**10**) recorded in acetone-d_6

Qualitative Analysis Report

**Data Filename**	150615ESINA1.d	**Sample Name**	xpp14
**Sample Type**	Sample	**Position**	
**Instrument Name**	Agilent G6230 TOF MS	**User Name**	KIB
**Acq Method**	ESIN.m	**Acquired Time**	6/15/2015 9:55:26 AM
**IRM Calibration Status**	Success	**DA Method**	ESI.m
**Comment**			

**Sample Group**		**Info.**
**Acquisition SW Version**	6200 series TOF/6500 series Q-TOF B.05.01 (B5125.2)	

User Spectra

Fragmentor Voltage	Collision Energy	Ionization Mode
200	0	ESI

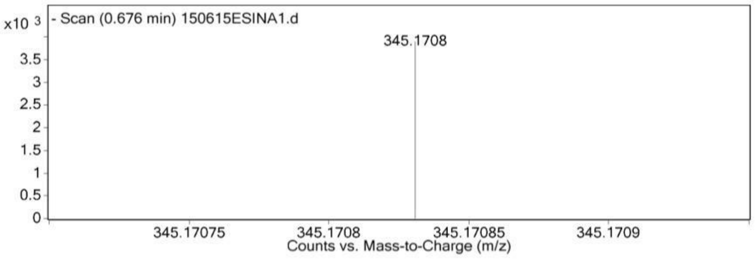

Peak List

m/z	z	Abund	Formula	Ion
112.9856		4183.03		
154.9737		1207.44		
248.9604		823.84		
255.2327		1334.95		
283.2642		813.4		
345.1708	1	3894.24	C20 H25 O5	M-
1033.9881	1	158790		
1034.9899	1	18040.02		
1933.9295	1	27216.98		
1934.9309	1	4623.55		

Formula Calculator Element Limits

Element	Min	Max
C	0	200
H	0	400
O	0	9

Formula Calculator Results

Formula	CalculatedMass	CalculatedMz	Mz	Diff. (mDa)	Diff. (ppm)	DBE
C20 H25 O5	345.1702	345.1707	345.1708	-0.1	-0.2	8.5000

--- End Of Report ---

Figure 88S. HRESIMS spectrum of (**10**)

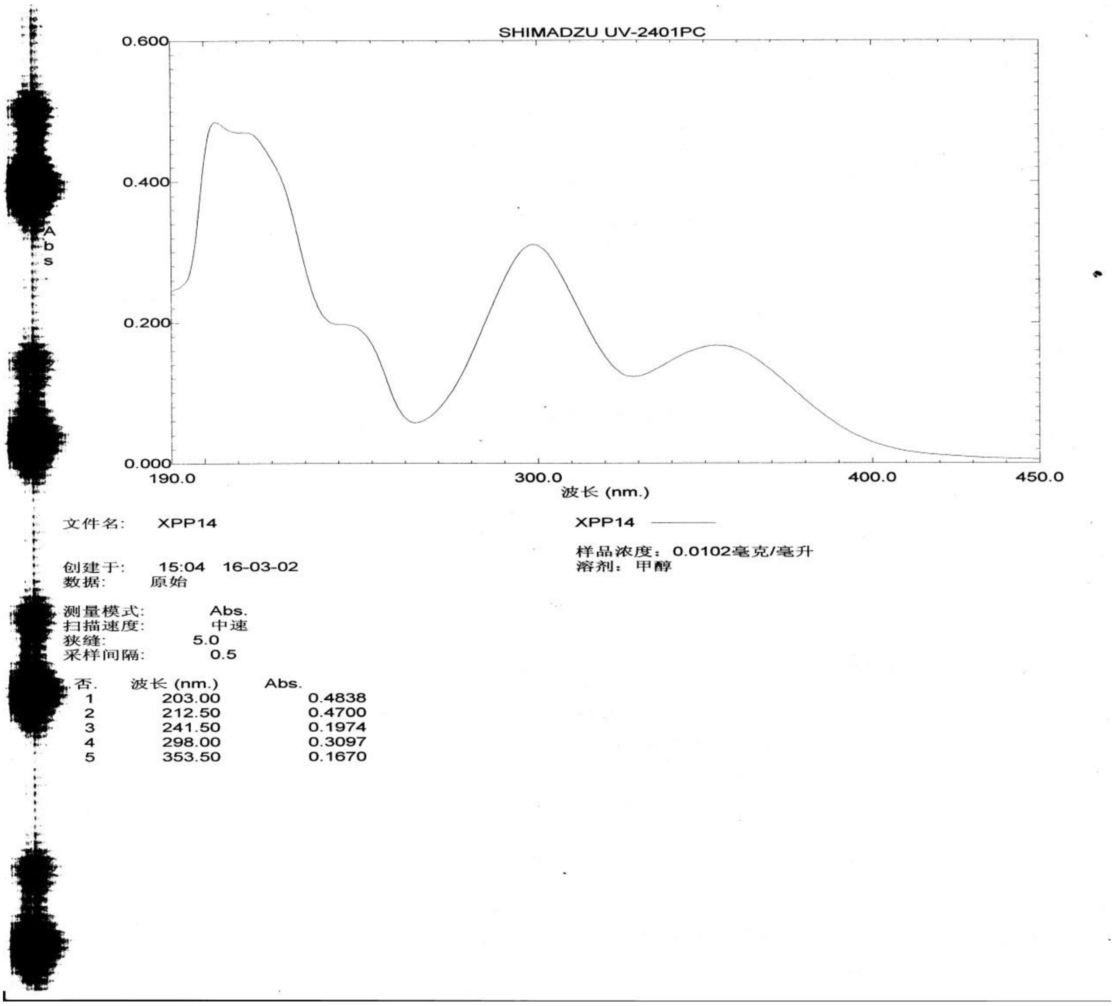

Figure 89S. UV spectrum of (**10**)

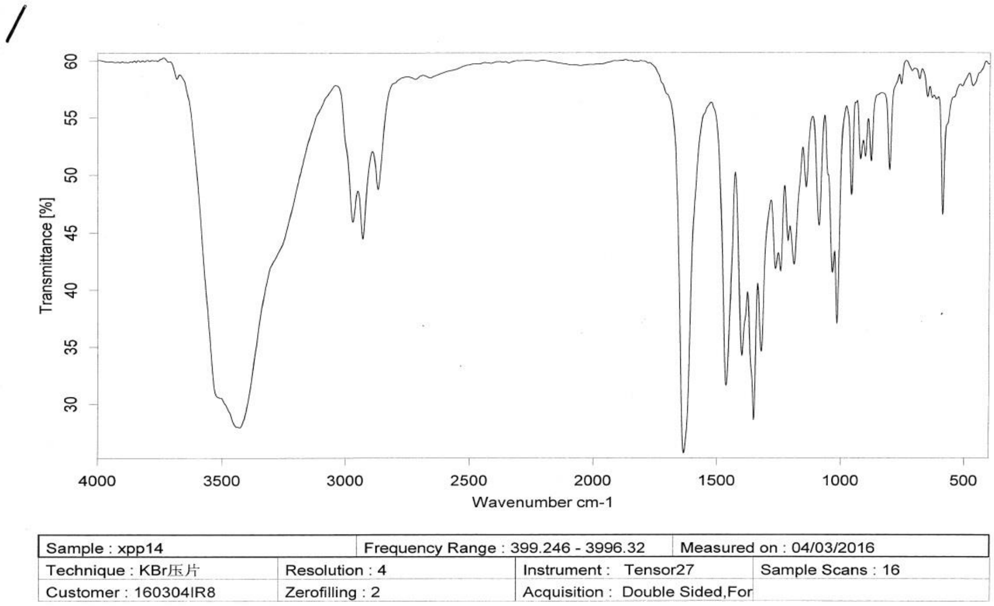

Figure 90S. IR spectrum of (**10**)

Figure 91S-99S. NMR, MS, UV, and IR spectra of compound 11

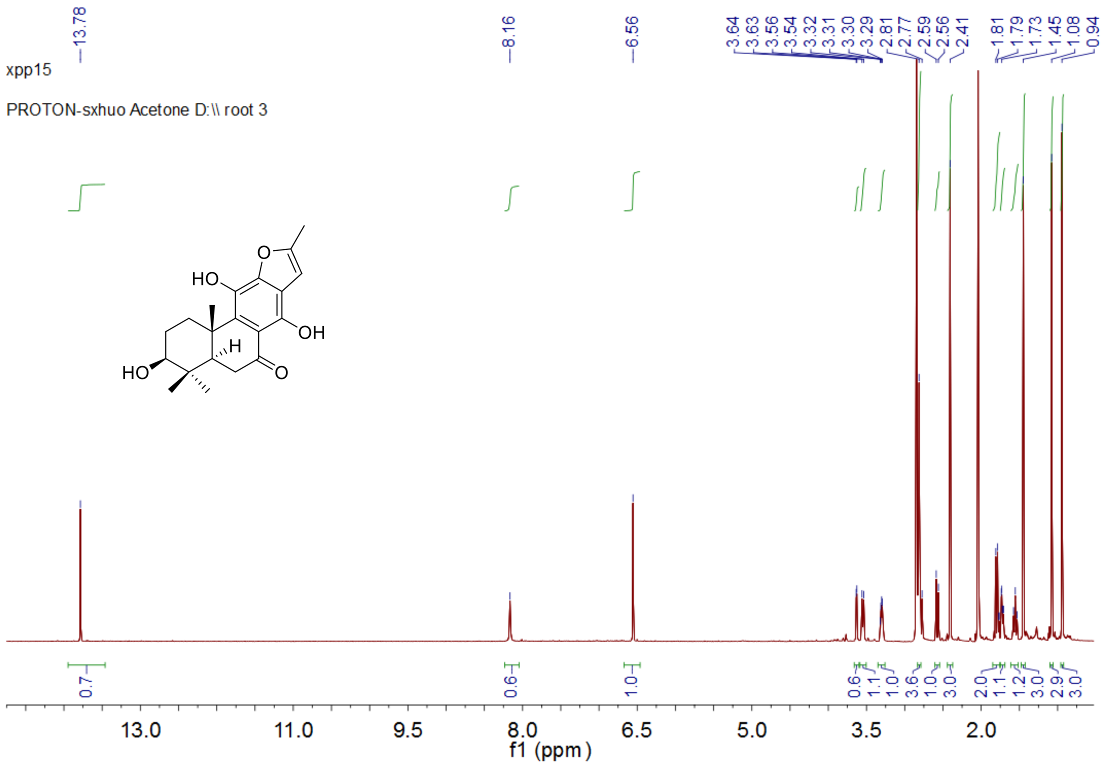

Figure 91S. ^{1}H NMR spectrum of (**11**) recorded in acetone-d_6 at 600 MHz

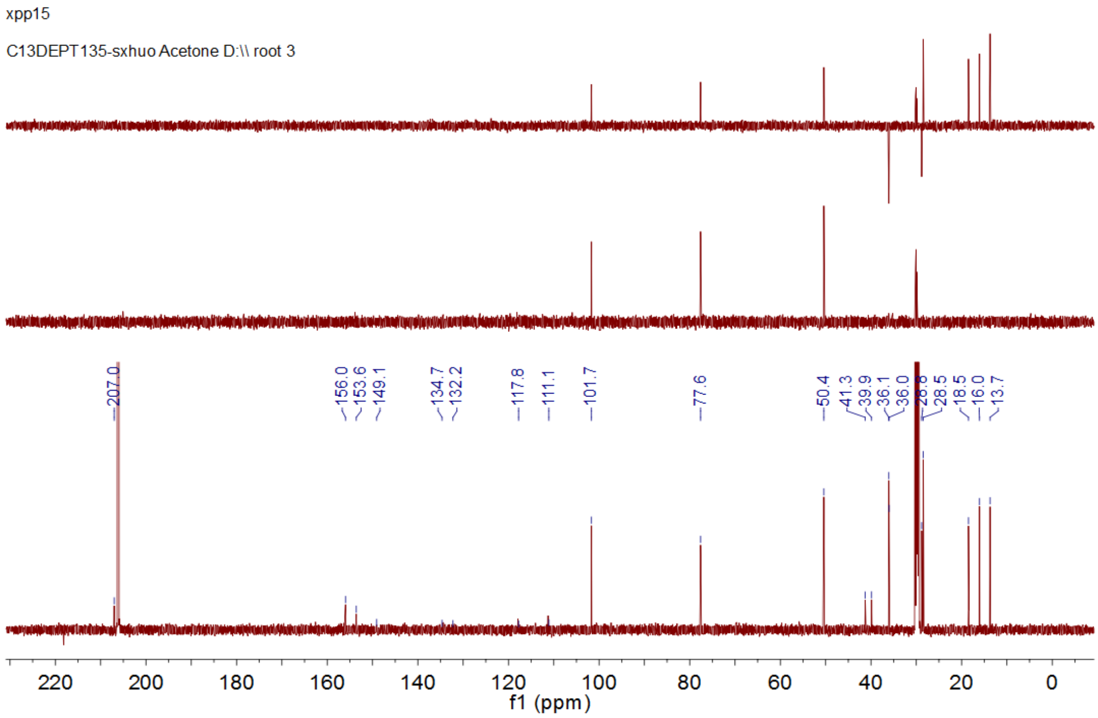

Figure 92S. ^{13}C NMR spectrum of (**11**) recorded in acetone-d_6 at 150 MHz

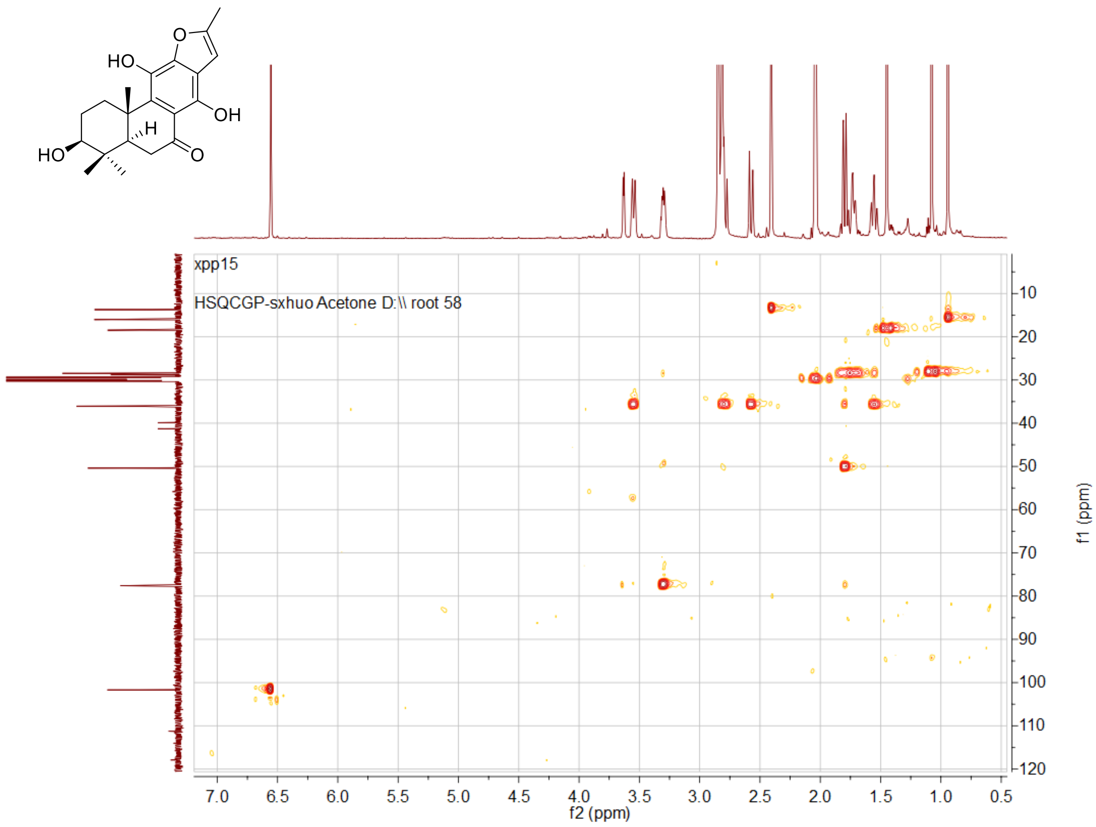

Figure 93S. HSQC spectrum of (**11**) recorded in acetone-d_6

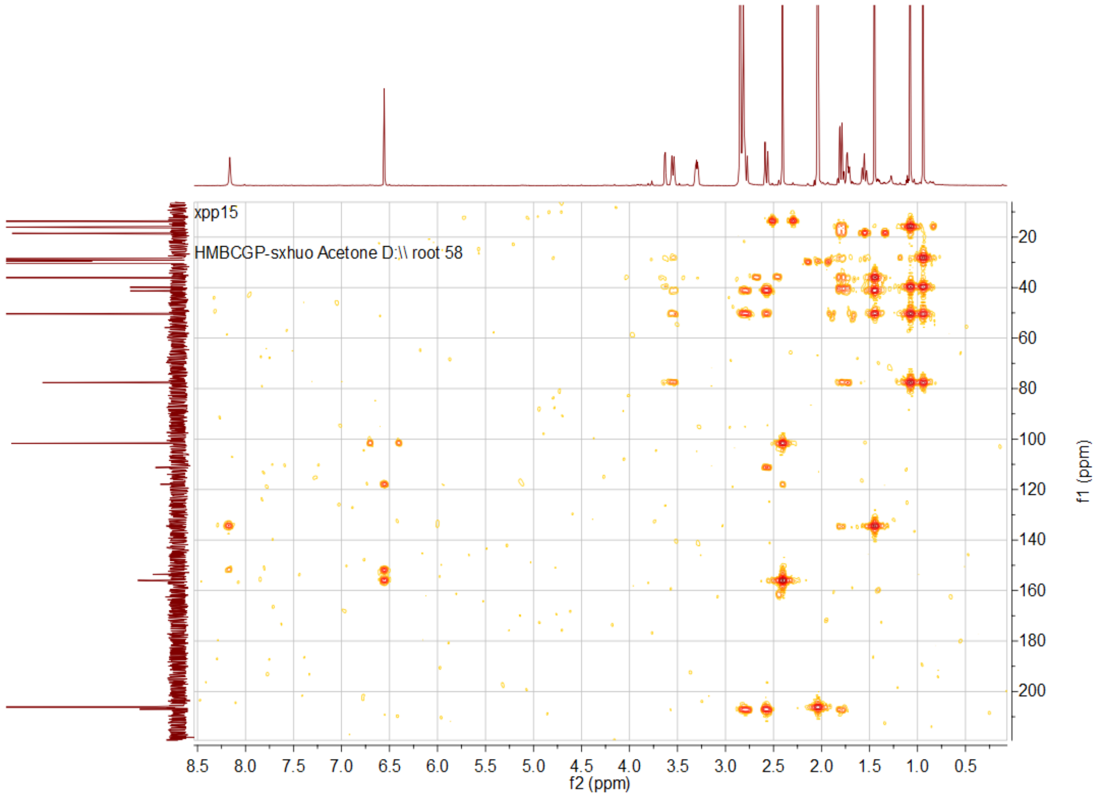

Figure 94S. HMBC spectrum of (**11**) recorded in acetone-d_6

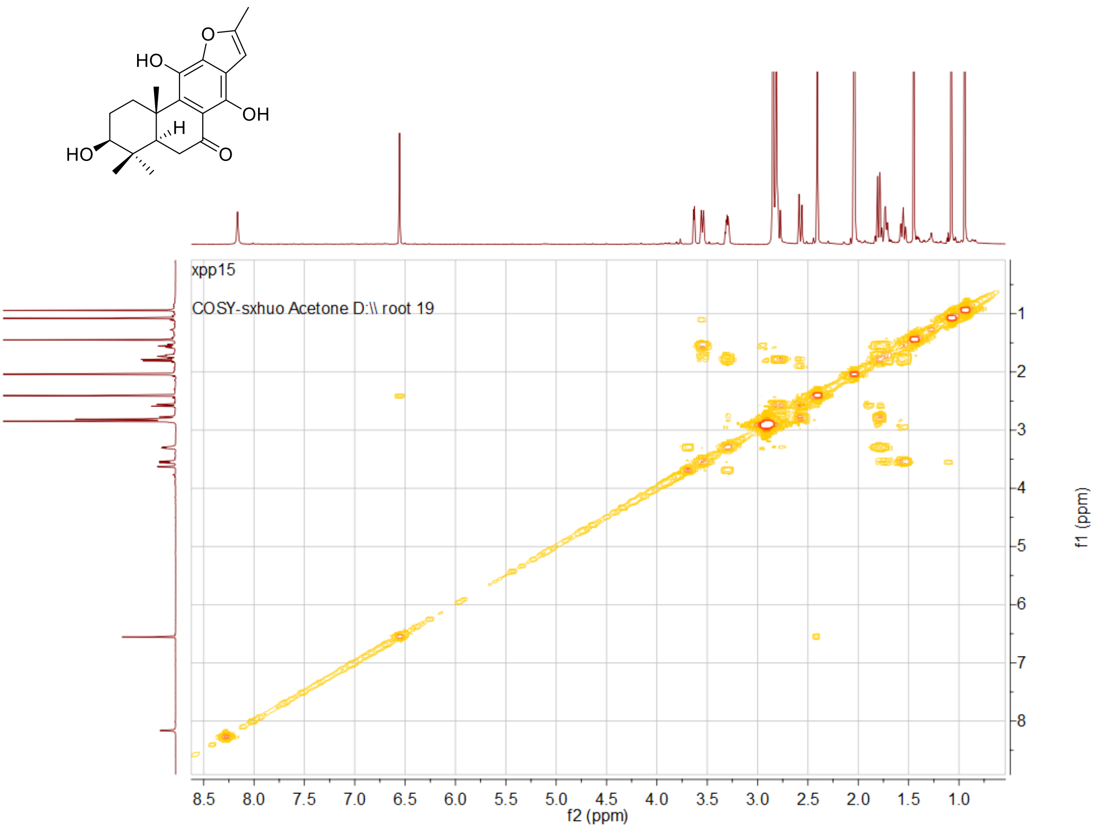

Figure 95S. ^1H-^1H COSYspectrum of (**11**) recorded in acetone-d_6

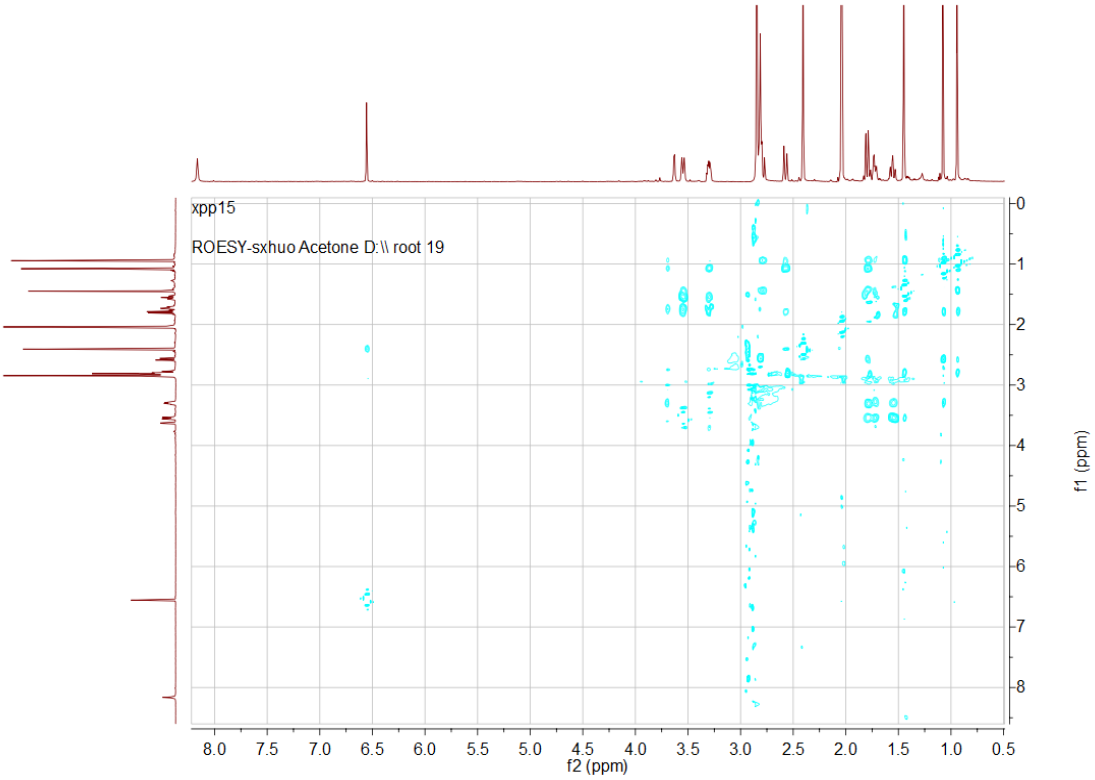

Figure 96S. ROESY spectrum of (**11**) recorded in acetone-d_6

Qualitative Analysis Report

**Data Filename**	150615ESINA4.d	**Sample Name**	xpp15
**Sample Type**	Sample	**Position**	
**Instrument Name**	Agilent G6230 TOF MS	**User Name**	KIB
**Acq Method**	ESIN.m	**Acquired Time**	6/15/2015 10:00:27 AM
**IRM Calibration Status**	Success	**DA Method**	ESI.m
**Comment**			

Sample Group **Info.**
Acquisition SW Version 6200 series TOF/6500 series Q-TOF B.05.01 (B5125.2)

User Spectra

Fragmentor Voltage	Collision Energy	Ionization Mode
200	0	ESI

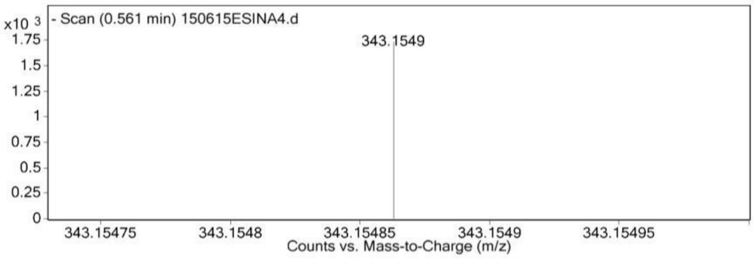

Peak List

m/z	z	Abund	Formula	Ion
112.9856		2430.78		
154.9739		692.11		
248.9613		442.03		
255.233		560.7		
343.1549	1	1729.98	C20 H23 O5	M-
1033.9881	1	168041.09		
1034.9893	1	19800.66		
1035.9908	1	938.98		
1933.9289	1	20614.34		
1934.9296	1	3247.07		

Formula Calculator Element Limits

Element	Min	Max
C	0	200
H	0	400
O	0	9

Formula Calculator Results

Formula	CalculatedMass	CalculatedMz	Mz	Diff. (mDa)	Diff. (ppm)	DBE
C20 H23 O5	343.1546	343.1551	343.1549	0.1	0.3	9.5000

--- End Of Report ---

Figure 97S. HRESIMS spectrum of (**11**)

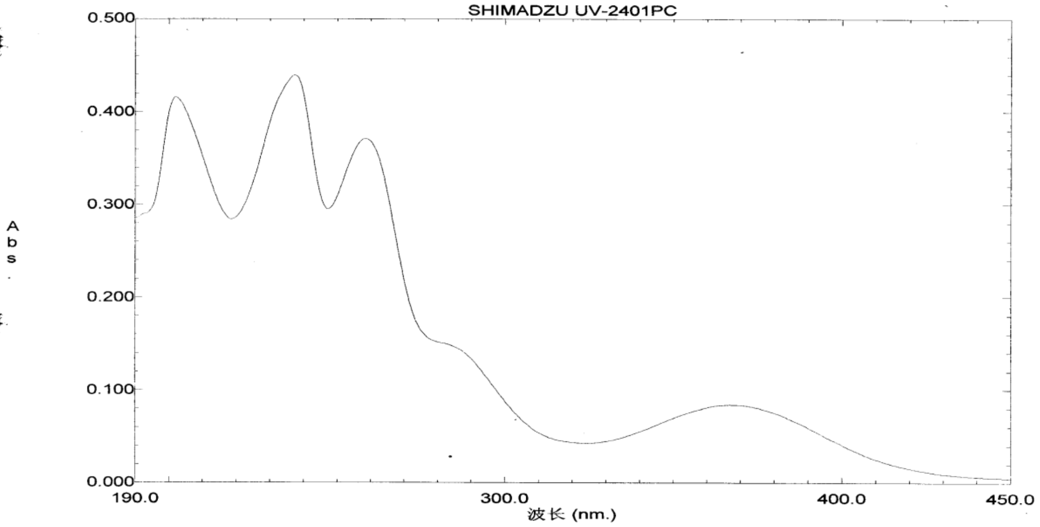

文件名: XPP15

XPP15 ———

创建于: 09:31 15-10-23
数据: 原始

样品浓度：0.0085毫克/毫升
溶剂：甲醇

测量模式: Abs.
扫描速度: 中速
狭缝: 5.0
采样间隔: 0.5

否.	波长 (nm.)	Abs.
1	202.00	0.4158
2	237.50	0.4391
3	258.50	0.3708
4	278.50	0.1537
5	369.50	0.0834

Figure 98S. UV spectrum of (**11**)

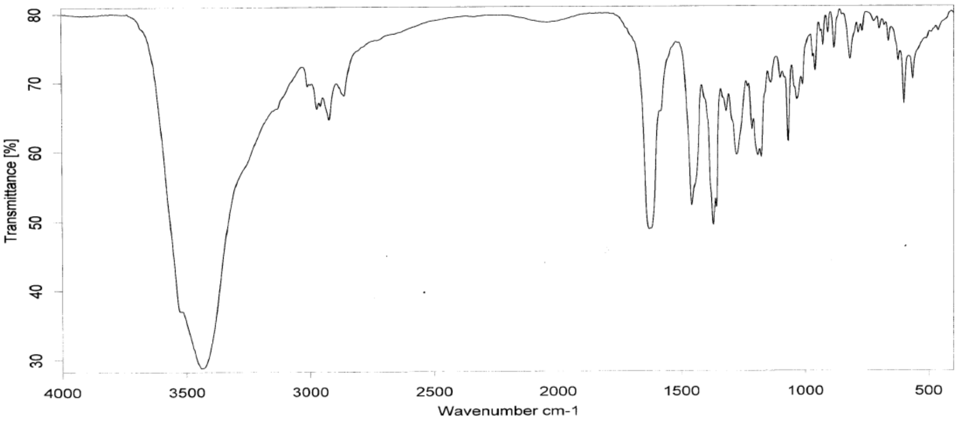

Sample : xpp15 Frequency Range : 399.246 - 3996.32 Measured on : 04/11/2015
Technique : KBr压片 Resolution : 4 Instrument : Tensor27 Sample Scans : 16
Customer : 151104IR02 Zerofilling : 2 Acquisition : Double Sided,For

Figure 99S. IR spectrum of (**11**)

Figure 100S-108S. NMR, MS, UV, and IR spectra of compound 12

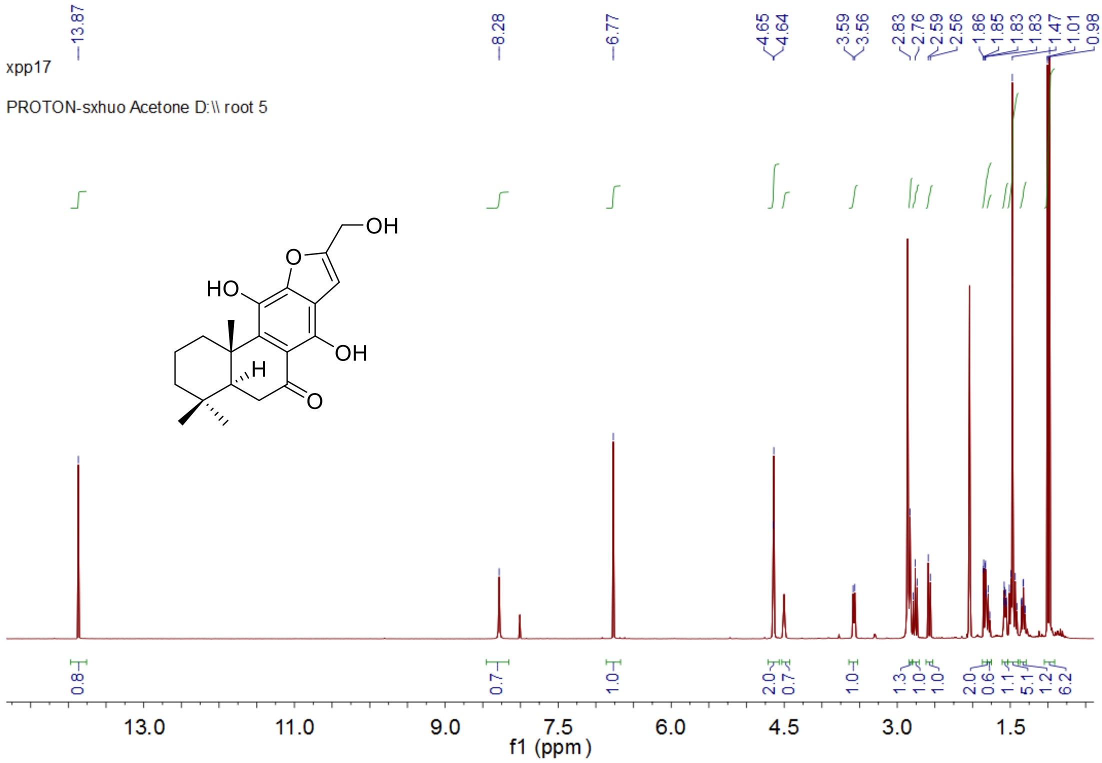

Figure 100S. ^{1}H NMR spectrum of (**12**) recorded in acetone-d_6 at 600 MHz

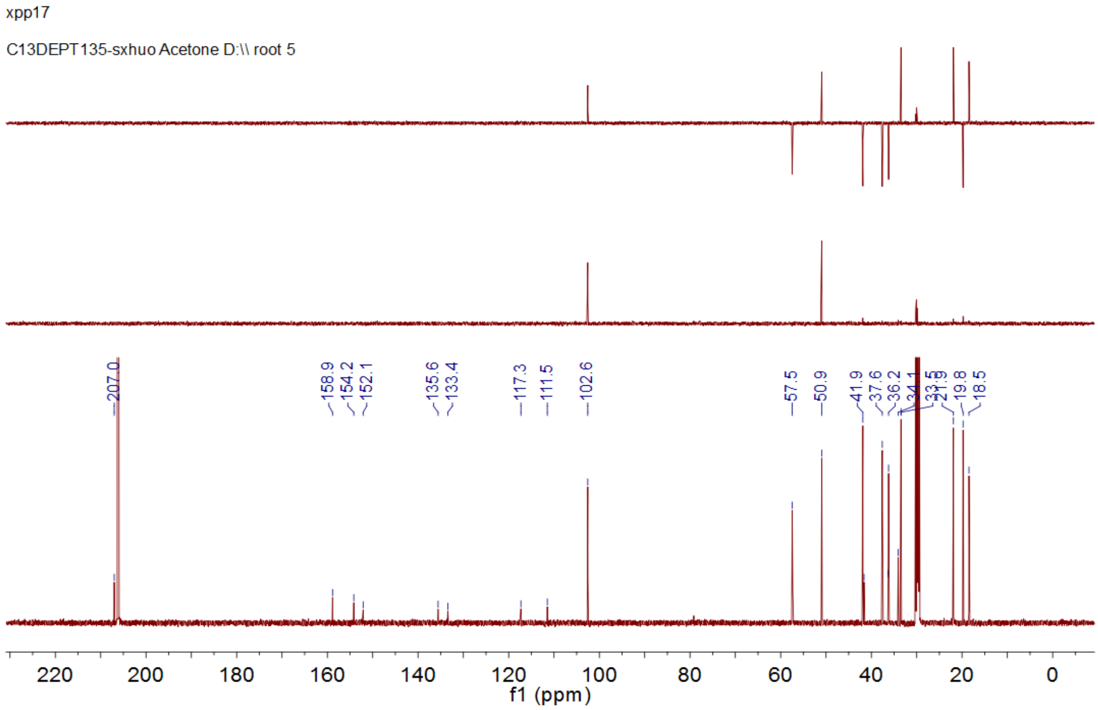

Figure 101S. ^{13}C NMR spectrum of (**12**) recorded in acetone-d_6 at 150MHz

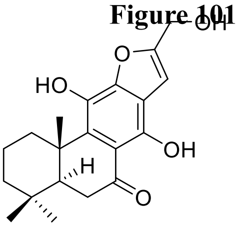

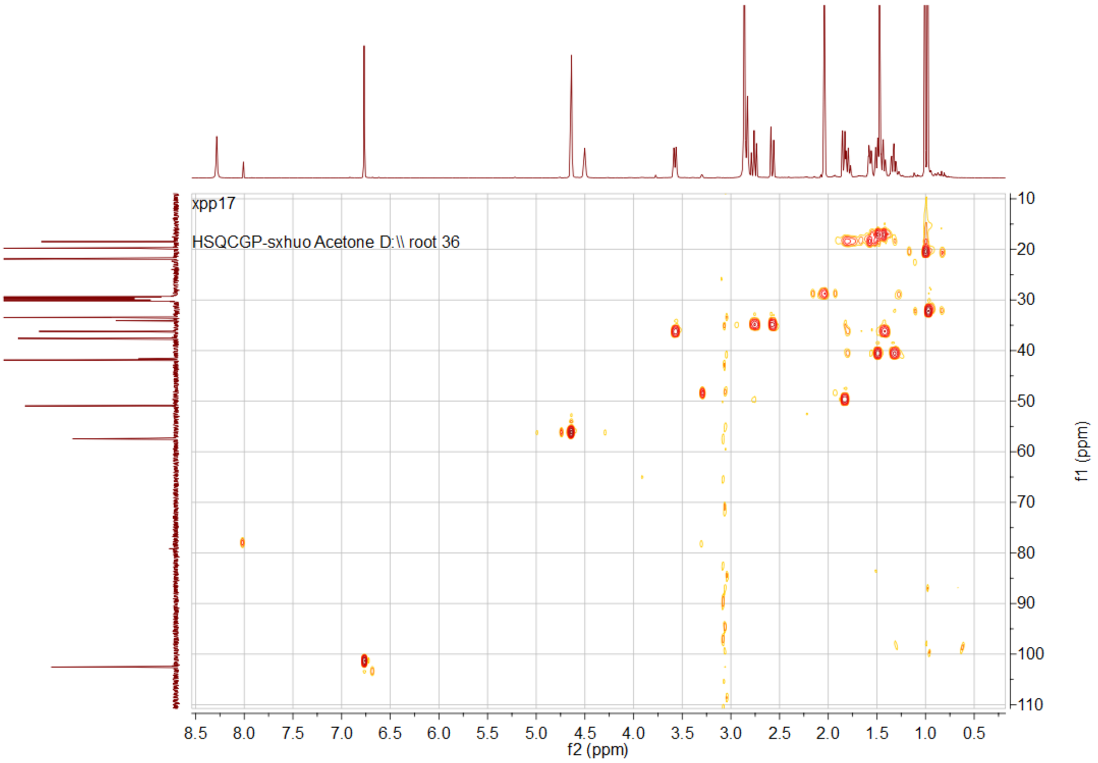

Figure 102S. HSQC spectrum of (**12**) recorded in acetone-d_6

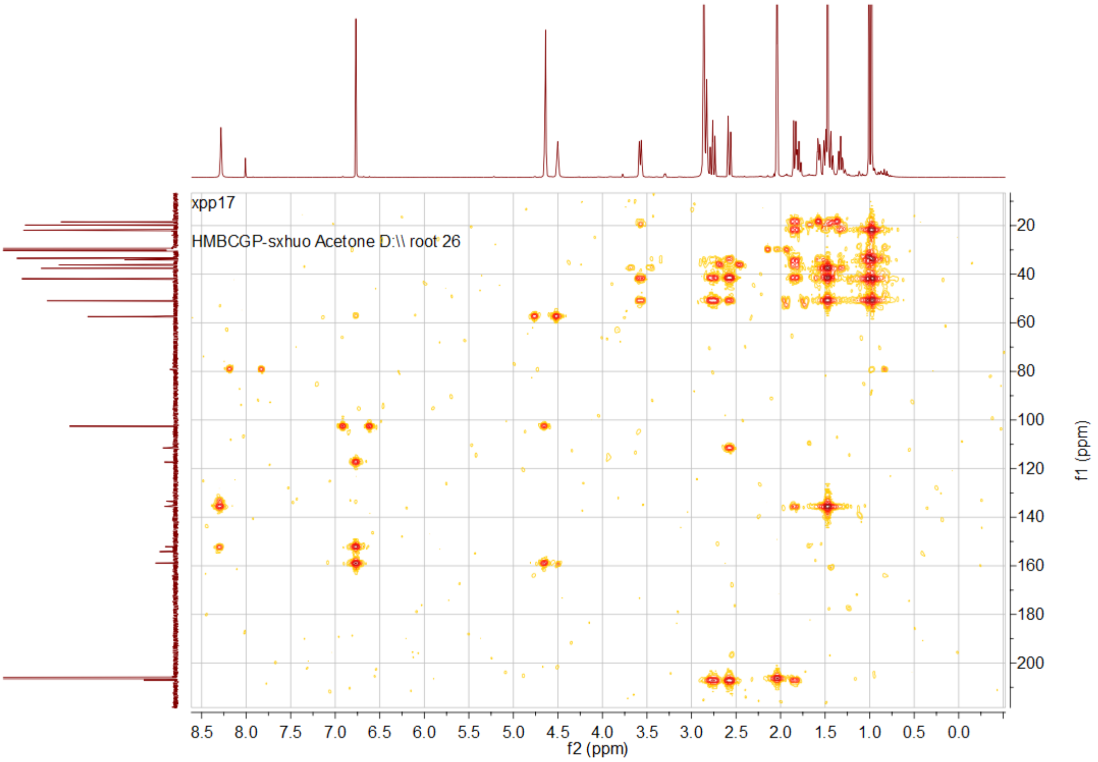

Figure 103S. HMBC spectrum of (**12**) recorded in acetone-d_6

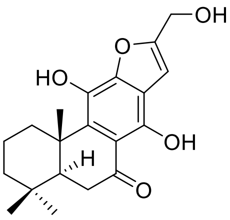

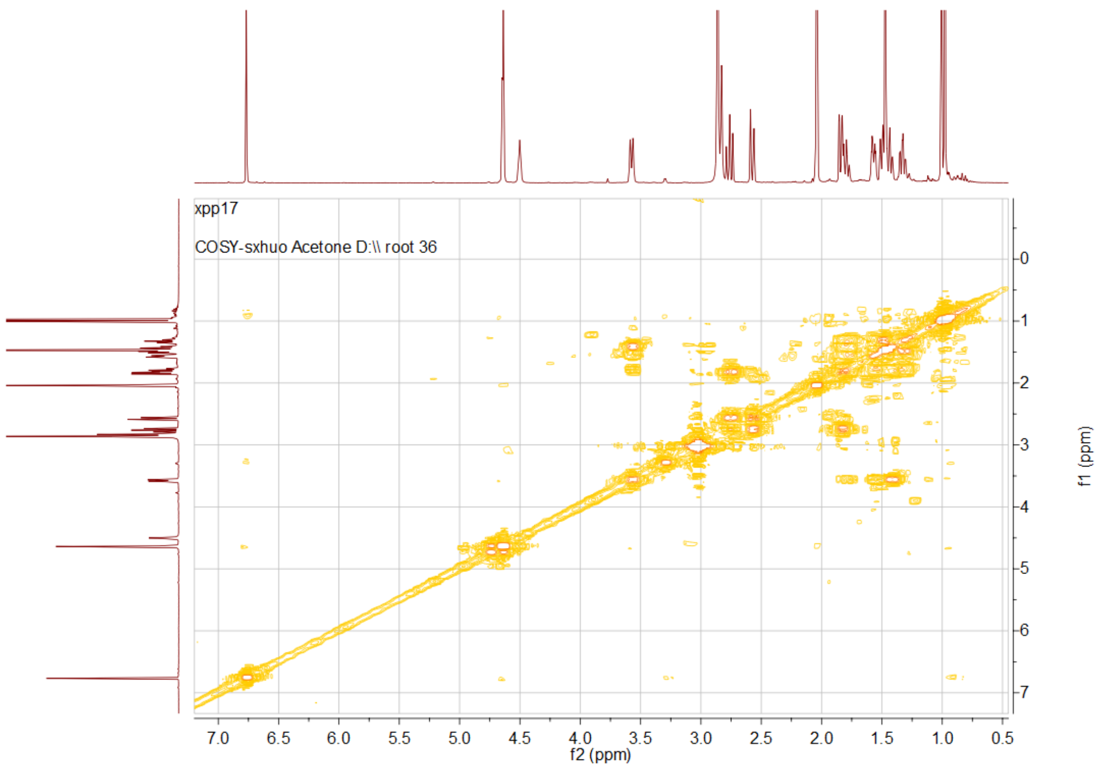

Figure 104S. ^{1}H-^{1}H COSY spectrum of (**12**) recorded in acetone-d_6

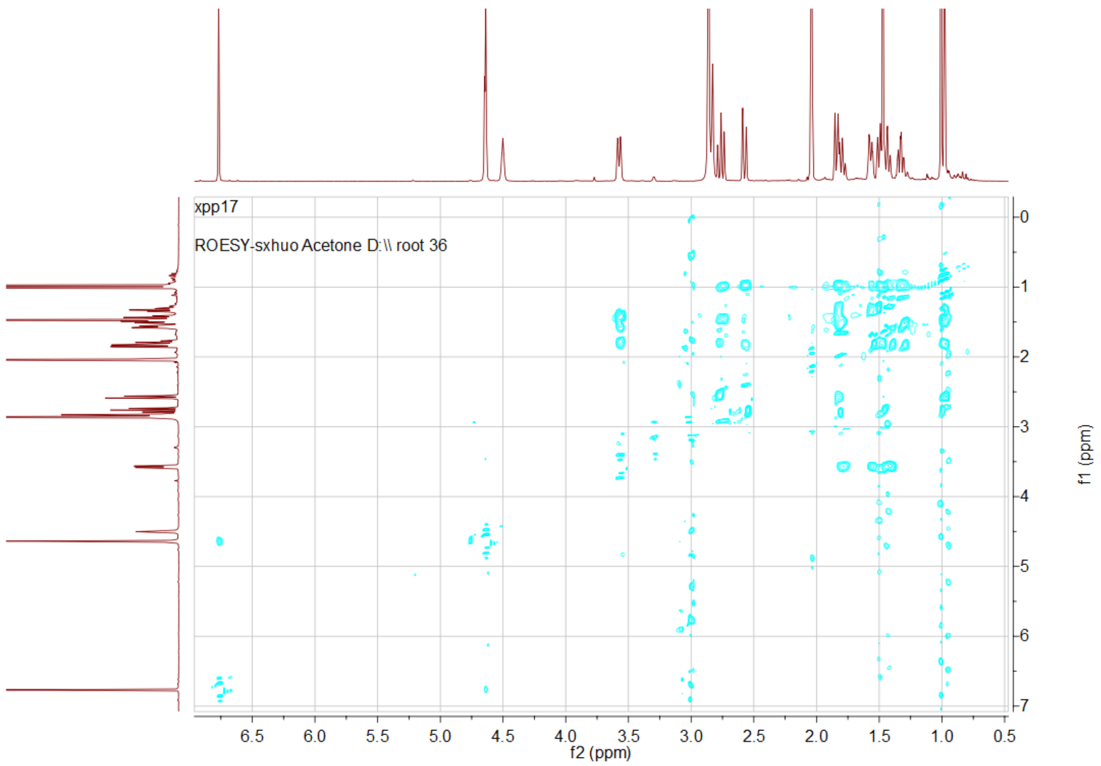

Figure 105S. ROESY spectrum of (**12**) recorded in acetone-d_6

Qualitative Analysis Report

**Data Filename**	150615ESINA3.d	**Sample Name**	xpp17
**Sample Type**	Sample	**Position**	
**Instrument Name**	Agilent G6230 TOF MS	**User Name**	KIB
**Acq Method**	ESIN.m	**Acquired Time**	6/15/2015 9:59:01 AM
**IRM Calibration Status**	Success	**DA Method**	ESI.m
**Comment**			

Sample Group **Info.**
Acquisition SW 6200 series TOF/6500 series
Version Q-TOF B.05.01 (B5125.2)

User Spectra

Fragmentor Voltage	Collision Energy	Ionization Mode
200	0	ESI

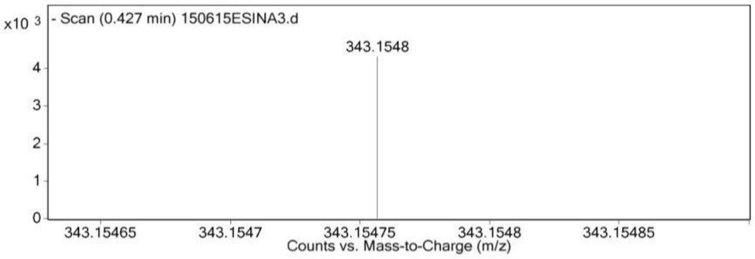

Peak List

m/z	z	Abund	Formula	Ion
112.9856		2794.83		
154.9735		716.01		
248.9605		431.28		
343.1548	1	4323.4	C20 H23 O5	M-
687.3153	1	707.84		
1033.9881	1	166463.09		
1034.9893	1	20252.85		
1035.9911	1	839.13		
1933.9291	1	20656.56		
1934.9311	1	3411.13		

Formula Calculator Element Limits

Element	Min	Max
C	0	200
H	0	400
O	0	9

Formula Calculator Results

Formula	CalculatedMass	CalculatedMz	Mz	Diff. (mDa)	Diff. (ppm)	DBE
C20 H23 O5	343.1546	343.1551	343.1548	0.4	1.2	9.5000

--- End Of Report ---

Figure 106S. HRESIMS spectrum of (**12**)

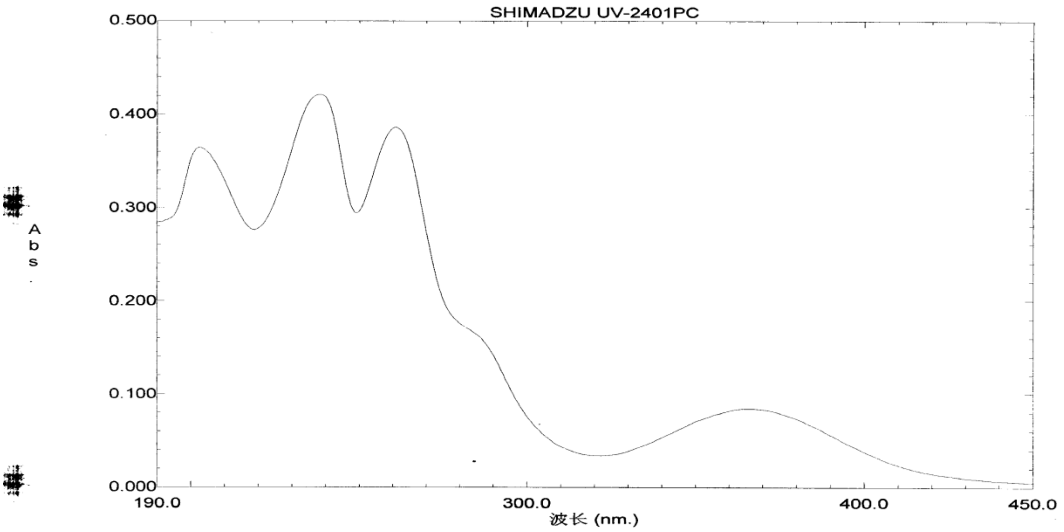

文件名: XPP17

XPP17

样品浓度：0.0127毫克/毫升
溶剂：甲醇

创建于: 09:55 15-10-23
数据: 原始

测量模式: Abs.
扫描速度: 中速
狭缝: 5.0
采样间隔: 0.5

否.	波长 (nm.)	Abs.
1	202.00	0.3638
2	237.50	0.4206
3	260.00	0.3847
4	279.50	0.1773
5	366.50	0.0841

Figure 107S. UV spectrum of (**12**)

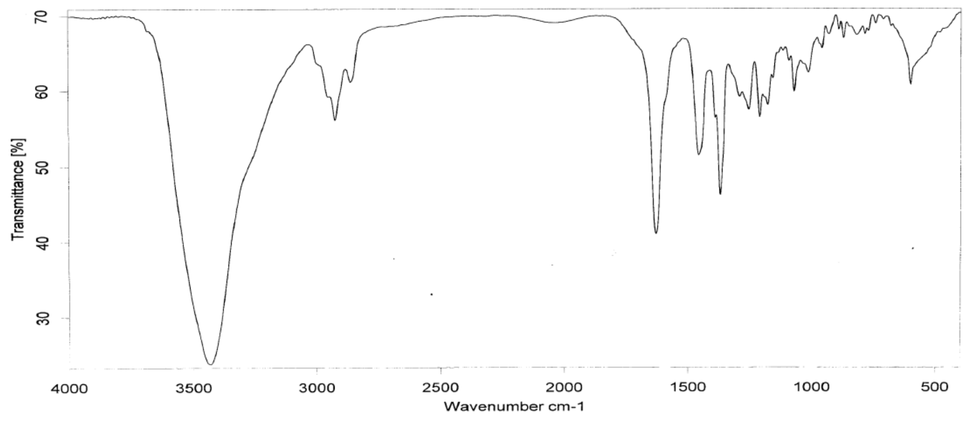

Sample : xpp17 Frequency Range : 399.246 - 3996.32 Measured on : 04/11/2015

Technique : KBr压片 Resolution : 4 Instrument : Tensor27 Sample Scans : 16

Customer : 151104IR01 Zerofilling : 2 Acquisition : Double Sided,For

Figure 108S. IR spectrum of (**12**)

Figure 109S. The pack drawing of compound 1

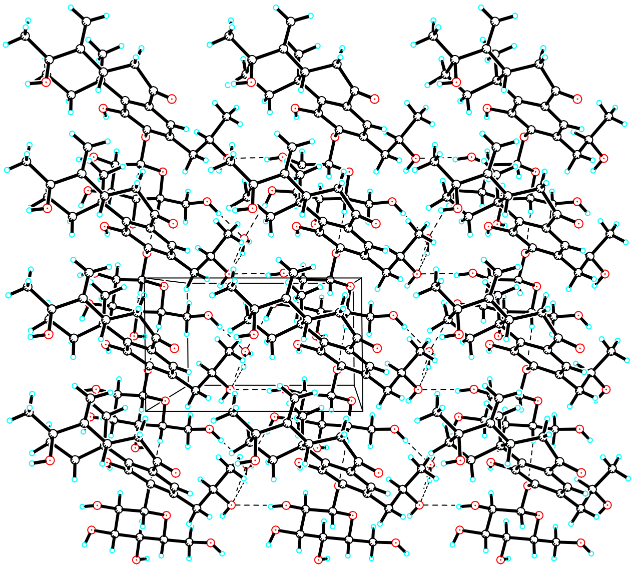

Figure 109S. View of the Pack drawing motif of **1**

(Hydrogen-bonds are shown as dashed lines)

Figure 110S. The pack drawing of compound 3

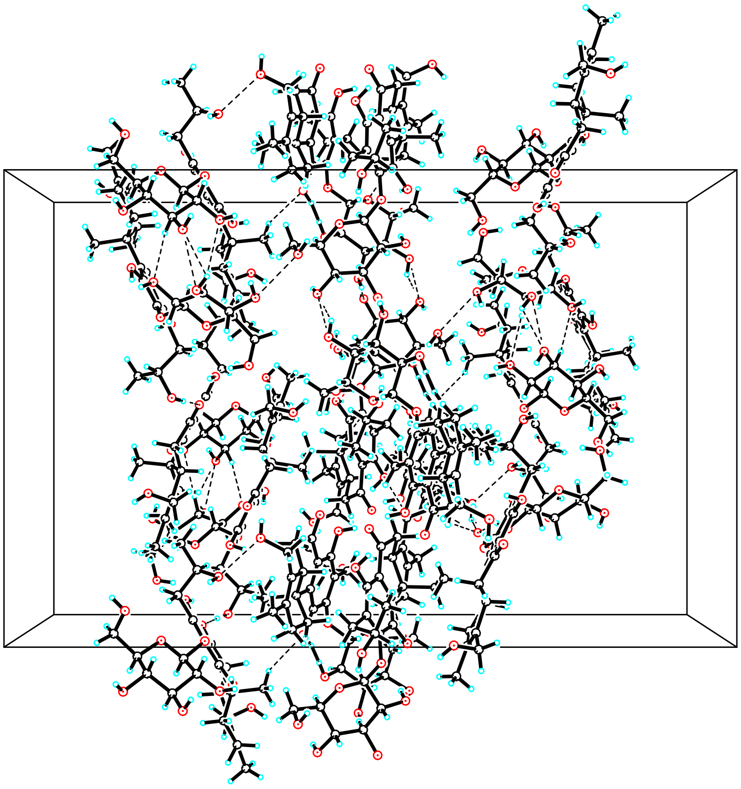

Figure 110S. View of the pack drawing of **3**.

(Hydrogen-bonds are shown as dashed lines)

Figure 111S. The pack drawing compound 10

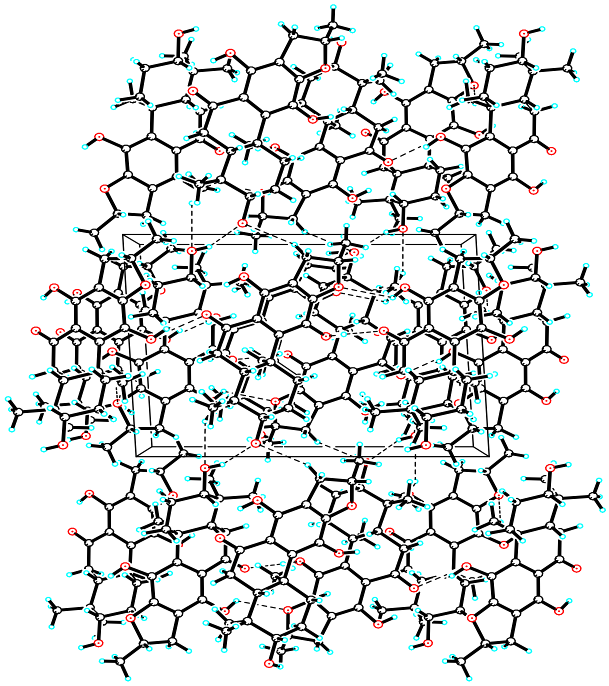

Figure 111S. View of the pack drawing of **10**

(Hydrogen-bonds are shown as dashed lines)

Table 1S. Crystal data and structure refinement for 1

Identification code	cu_xpp40_0m
Empirical formula	C26 H38 O11
Formula weight	526.56
Temperature	100(2) K
Wavelength	1.54178 A
Crystal system, space group	Monoclinic, P 21
Unit cell dimensions	a = 5.70480(10) A alpha = 90 deg.
	b = 23.8602(5) A beta = 90.6040(10) deg.
	c = 9.3419(2) A gamma = 90 deg.
Volume	1271.53(4) A^3
Z, Calculated density	2, 1.375 Mg/m^3
Absorption coefficient	0.898 mm^-1
F(000)	564
Crystal size	0.67 x 0.62 x 0.38 mm
Theta range for data collection	3.70 to 69.31 deg.
Limiting indices	-6<=h<=6, -26<=k<=24, -11<=l<=11
Reflections collected / unique	10232 / 3499 [R(int) = 0.0328]
Completeness to theta = 69.31	93.3 %
Absorption correction	Semi-empirical from equivalents
Max. and min. transmission	0.7267 and 0.5846
Refinement method	Full-matrix least-squares on F^2
Data / restraints / parameters	3499 / 1 / 344
Goodness-of-fit on F^2	1.113
Final R indices [I>2sigma(I)]	R1 = 0.0300, wR2 = 0.0884
R indices (all data)	R1 = 0.0300, wR2 = 0.0885
Absolute structure parameter	0.17(14)

Extinction coefficient	0.0128(8)
Largest diff. peak and hole	0.222 and -0.238 e.A^-3

Table 2S. Crystal data and structure refinement for 3

Identification code	cu_xpp57_0m-sr	
Empirical formula	C104 H146 O45	
Formula weight	2116.20	
Temperature	100(2) K	
Wavelength	1.54178 Å	
Crystal system	Orthorhombic	
Space group	$P2_12_12_1$	
Unit cell dimensions	a = 17.5477(6) Å	α= 90°.
	b = 21.7199(7) Å	β= 90°.
	c = 33.3592(12) Å	γ = 90°.
Volume	12714.3(8) $Å^3$	
Z	4	
Density (calculated)	1.106 Mg/m^3	
Absorption coefficient	0.728 mm^{-1}	
F(000)	4520	
Crystal size	0.980 x 0.660 x 0.470 mm^3	
Theta range for data collection	2.427 to 69.708°.	
Index ranges	-21<=h<=21, -26<=k<=25, -37<=l<=40	
Reflections collected	112641	
Independent reflections	23429 [R(int) = 0.0484]	
Completeness to theta = 67.679°	99.7 %	
Absorption correction	Semi-empirical from equivalents	
Refinement method	Full-matrix least-squares on F^2	

Data / restraints / parameters	23429 / 9 / 1386
Goodness-of-fit on F^2	1.044
Final R indices [I>2sigma(I)]	R1 = 0.0744, wR2 = 0.2065
R indices (all data)	R1 = 0.0759, wR2 = 0.2083
Absolute structure parameter	0.11(3)
Extinction coefficient	n/a
Largest diff. peak and hole	1.030 and -0.370 e.Å^{-3}

Table 3S. Crystal data and structure refinement for 10

Identification code	cu_xpp14_0m	
Empirical formula	C20 H26 O5	
Formula weight	346.41	
Temperature	100(2) K	
Wavelength	1.54178 A	
Crystal system, space group	Monoclinic, P 21	
Unit cell dimensions	a = 11.5843(7) A	alpha = 90 deg.
	b = 9.5501(6) A	beta = 92.859(4) deg.
	c = 15.2093(10) A	gamma = 90 deg.
Volume	1680.53(18) A^3	
Z, Calculated density	4, 1.369 Mg/m^3	
Absorption coefficient	0.794 mm^-1	
F(000)	744	
Crystal size	0.40 x 0.28 x 0.02 mm	
Theta range for data collection	2.91 to 69.25 deg.	
Limiting indices	-14<=h<=13, -11<=k<=11, -18<=l<=17	
Reflections collected / unique	10384 / 4801 [R(int) = 0.0536]	

Completeness to theta = 69.25	92.0 %
Absorption correction	Semi-empirical from equivalents
Max. and min. transmission	0.9843 and 0.7419
Refinement method	Full-matrix least-squares on F^2
Data / restraints / parameters	4801 / 1 / 464
Goodness-of-fit on F^2	1.058
Final R indices [I>2sigma(I)]	R1 = 0.0627, wR2 = 0.1682
R indices (all data)	R1 = 0.0701, wR2 = 0.1743
Absolute structure parameter	0.0(2)
Largest diff. peak and hole	0.444 and -0.561 e.A^-3
